# Fine Tuning of Catalyst Active Sites to Access Advanced Ultrahigh‐Melting Polyolefins

**DOI:** 10.1002/anie.9519061

**Published:** 2026-09-21

**Authors:** Fabio De Stefano, Olga D'Anania, Giovanni Talarico, Claudio De Rosa

**Affiliations:** ^1^ Dipartimento Di Scienze Chimiche Università Di Napoli Federico II Napoli Italy; ^2^ Scuola Superiore Meridionale Napoli Italy

**Keywords:** allyltrimethylsilane, bulky monomers, high‐melting polyolefins, monomer‐induced activation, pyridylamido‐Hf catalyst

## Abstract

Polyolefins with melting temperatures above 200°C are rare, as their synthesis typically relies on the polymerization of highly sterically congested α‐olefins, which is challenging for both industrial Ziegler–Natta and homogeneous catalysts. Pyridylamido‐Hf complexes are molecular catalysts known for their high activity and ability to incorporate sterically demanding monomers, for example, allyltrimethylsilane (ATMS) giving isotactic poly(allyltrimethylsilane) with a melting temperature of 310°C and high turnover frequency. However, they are unable to polymerize efficiently monomers such as vinylcyclohexane (VCH) or 4,4‐dimethyl‐1‐pentene (44DMP). In this study, we report a novel synthetic strategy that enables the polymerization of such sterically challenging monomers, based on a pre‐activation of the Hf catalyst by using ATMS as ligand modifier in situ. We show that ATMS acts as an effective pre‐activator of the Hf complex, enabling controlled formation of highly active species and unlocking the polymerization of sterically demanding α‐olefins. This afforded the first synthesis of isotactic poly(4,4‐dimethyl‐1‐pentene) with an unprecedented melting temperature of 420°C, and efficient polymerization of VCH and 3‐methyl‐1‐butene. These findings demonstrate how the tuning of the pyridylamido‐Hf active species through rational choice of the activating monomer can be leveraged to expand the access to ultrahigh melting temperature polyolefins.

## Graphical abstract

A redesign of the active site of the pyridylamido hafnium (Hf) catalysts can enhance the performance of this catalyst family and unlock previously inaccessible polymer structures. When the catalyst is precontacted with allyltrimethylsilane the active species is modified and the catalyst experiences a new life becoming able to polymerize sterically challenging bulky α‐olefins to ultrahigh‐melting polyolefins never produced before.

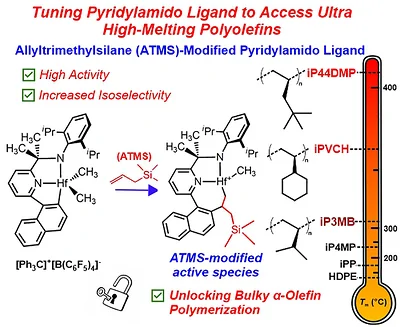

## 1 Introduction

Polyolefins (POs) with exceptionally high melting temperatures (*T*_m_) represent a long‐standing challenge in polymer science. Increasing the melting point over 200°C is highly desirable, as it would extend the application window of POs into thermal regimes typically associated with high‐performance polymers such as poly(ether ether ketone) (PEEK) [1], poly(phenylene sulfide) (PPS) [2], or fluoropolymers including poly(tetrafluoroethylene) (PTFE) [3, 4], while preserving their low density and chemically simple hydrocarbon backbone. Furthermore, POs with extremely high melting temperatures allow for applications that are limited to thermosetting resins, but with the great advantage of retaining their thermoplastic nature and, therefore, potential mechanical recyclability. These properties are currently inaccessible to biodegradable or chemically recyclable polymers, such as polyesters.

POs with high *T*_m_ are generally accessed through the polymerization of sterically demanding α‐olefins with Ziegler–Natta (ZN) catalysis [5]. Isotactic poly(4‐methyl‐1‐pentene) (iP4MP) stands out as the only example that has achieved large‐scale industrial relevance [6]. It combines a high melting temperature (*T*_m_ ≈ 240°C) with extremely low density, excellent optical transparency, and chemical inertness [7, 8, 9]. These features have enabled its use in high‐performance applications such as optical components, gas‐permeable membranes, energy storage applications and medical devices, where thermal stability and optical clarity are simultaneously required [8, 10]. Other isotactic POs derived from bulky monomers, including isotactic poly(3‐methyl‐1‐butene) (iP3MB) (*T*_m_ ≈ 300°C) or isotactic poly(vinylcyclohexane) (iPVCH) (*T*_m_ ≈ 370°C), have been reported but remain niche materials [11, 12]. iP3MB exhibits high crystallinity and thermal resistance, yet its limited processability has largely confined its relevance to academic studies. In contrast, iPVCH has found application as an efficient nucleating agent for the crystallization of the α form of isotactic polypropylene (iPP), rather than for direct use as a standalone polymer material [13, 14]. Very recently, an ethylene‐based high‐melting polyolefin, isotactic poly(4‐ethylhex‐1‐ene) (P4EH), was reported to melt above 200°C [15], demonstrating that appropriately designed bulky α‐olefins can push polyolefin melting temperatures well beyond those of conventional materials [16].

Owing to the pronounced steric congestion associated with these monomers, their polymerization has been dominated by heterogeneous ZN catalysts, which combine high robustness, productivity, stereoselectivity and excellent control over polymer particle morphology [14, 17, 18]. Despite their outstanding performances, however, the multisite nature of active species restricts the fine control of polymer architectures and the molecular‐level insight [19]. By contrast, homogeneous single‐site catalysts provide a well‐defined coordination environment, enabling precise control over polymer microstructure, comonomer distribution and stereoregularity, and development of rational structure‐property relationships [20, 21, 22]. Pyridylamido‐Hf complexes (Scheme 1) constitute an industrially relevant class of post‐metallocene catalysts used in Dow solution process at high temperature [23, 24] that are known for their high activity and ability to incorporate sterically demanding monomers [25, 26, 27, 28]. Their performance, however, strongly depends on monomer structure: while extended, linearly bulky α‐olefins are efficiently polymerized, monomers bearing substituents near the C═C bond, such as 3‐methyl‐1‐butene (3MB) and vinylcyclohexane (VCH), that is, those giving high melting temperature POs, typically exhibit poor reactivity [29].

**SCHEME 1:**
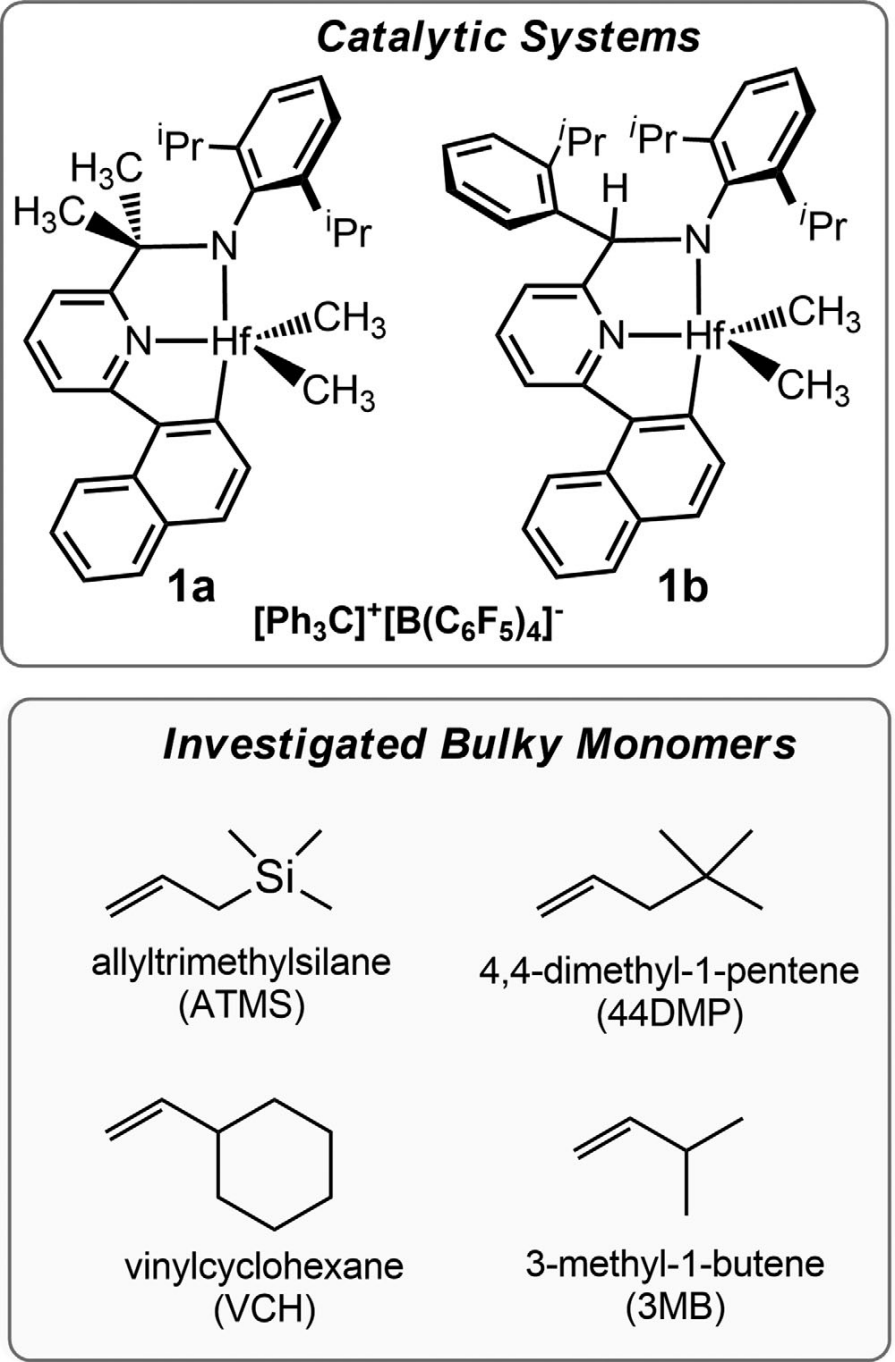
Pyridylamido‐Hf catalysts and investigated bulky monomers.

In this study, we report a novel synthetic strategy that enables the homogeneous polymerization of sterically challenging monomers, such as 4,4‐dimethyl‐1‐pentene (44DMP), 3MB and VCH (Scheme 1), which all give high melting temperature POs, through an ad hoc methodology designed to overcome the limitation of pyridylamido‐Hf catalysts that is associated with their mechanism of active species generation. The strategy is based on a modification of the active species of the pyridylamido‐Hf catalyst by using allyltrimethylsilane (ATMS) as ligand modifier in situ. We show that ATMS acts as an effective ligand‐modifier of the Hf complex, enabling controlled formation of highly active species and unlocking the homogeneous polymerization of sterically demanding α‐olefins. The remarkably high *T*_m_ of the resulting polymers combined with the catalytic activities achieved in the homopolymerization of bulky monomers well exceed the data reported in literature, thereby extending POs into the realm of ultrahigh‐melting materials.

## 2 Results and Discussion

To assess the suitability of pyridylamido‐Hf catalysts for the polymerization of highly bulky α‐olefins, we selected ATMS as a benchmark monomer (Scheme 1). To date, the polymerization of ATMS has been largely restricted to heterogeneous ZN systems [30] and few specific metallocene catalysts (Scheme S1 and Table S1) [31, 32]. Isotactic poly(allyltrimethylsilane) (iPATMS) is of interest both for high‐temperature applications (*T*_m_ ≈ 300°C) [30, 31] and, as reported more recently, as efficient nucleating agent for isotactic polypropylene (iPP) inducing crystallization of the tough β form [14].

The pyridylamido‐Hf complex employed in this study (**1a** in Scheme 1) [33, 34] differs from the industrially implemented analogue (**1b** in Scheme 1) that is known to exhibit higher isoselectivity in propene polymerization [23]. Specifically, the selected catalyst **1a** of Scheme 1 features a dimethyl‐substituted Me_2_C bridge [33, 34], resulting in a less sterically encumbered catalytic pocket, whereas **1b** bears a more cumbersome unsymmetrically substituted ^*i*^PrPhHC bridge. As a result, the industrial catalyst **1b** exhibits higher isoselectivity in propene polymerization than the catalyst **1a**, dictated by the direct ligand‐monomer interactions [35], as reflected by the higher melting temperature of the corresponding produced iPP (*T*_m_ ≈ 160°C for catalyst **1b** and ≈133°C for the catalyst **1a**) [33, 36].

Catalyst **1a** was selected as the reference system because its non‐stereogenic dimethyl‐substituted bridge provides a structurally simpler and molecularly better‐defined platform. After the first insertion into the Hf─C_Aryl_ bond, the resulting ligand‐modified species is more straightforwardly defined from a stereochemical standpoint, facilitating mechanistic interpretation and computational analysis. By contrast, the stereogenic, unsymmetrically substituted bridge of catalyst **1b** may allow multiple diastereomeric insertion pathways and generate distinct ligand‐modified species [37, 38].

Although the catalyst **1a** shows lower isoselectivity in propene polymerization than **1b**, we hypothesized that, due to the large steric hindrance in the selected bulky α‐olefins, no relevant decrease in isoselectivity should be observed in the polymerizations of these highly bulky α‐olefin monomers by using **1a**.

When polymerized with the catalyst **1a** activated with [Ph_3_C]^+^[B(C_6_F_5_)_4_]^−^, according to the standard polymerization procedure (procedure 1 in Scheme 2), ATMS unexpectedly exhibited outstanding reactivity with high catalytic activity, and iPATMS with high yield has been obtained (Table 1, entry 1). Moreover, polymerization proceeds with a turnover frequency (TOF) that exceeds those reported for heterogeneous ZN or homogeneous metallocene catalysts [30, 31, 32] (Tables 1 and S1). The resulting polymer iPATMS displays a melting temperature of 308°C (curve b of Figure 1a and Figure S1), exceeding the *T*_m_ values (≈ 305°C) reported for iPATMS samples obtained via either conventional heterogeneous ZN catalysts or homogeneous metallocene catalysts (Tables 1 and S1) [30, 31, 32]. Due to its insolubility in common organic solvents, solution NMR analysis was precluded. However, wide‐angle X‐ray diffraction (WAXS) (profile b of Figure 1b) and Fourier‐transform infrared spectroscopy (FT‐IR) (Figure S2) analyses are consistent with an isotactic microstructure, in agreement with previous literature reports for iPATMS [31, 32].

**SCHEME 2:**
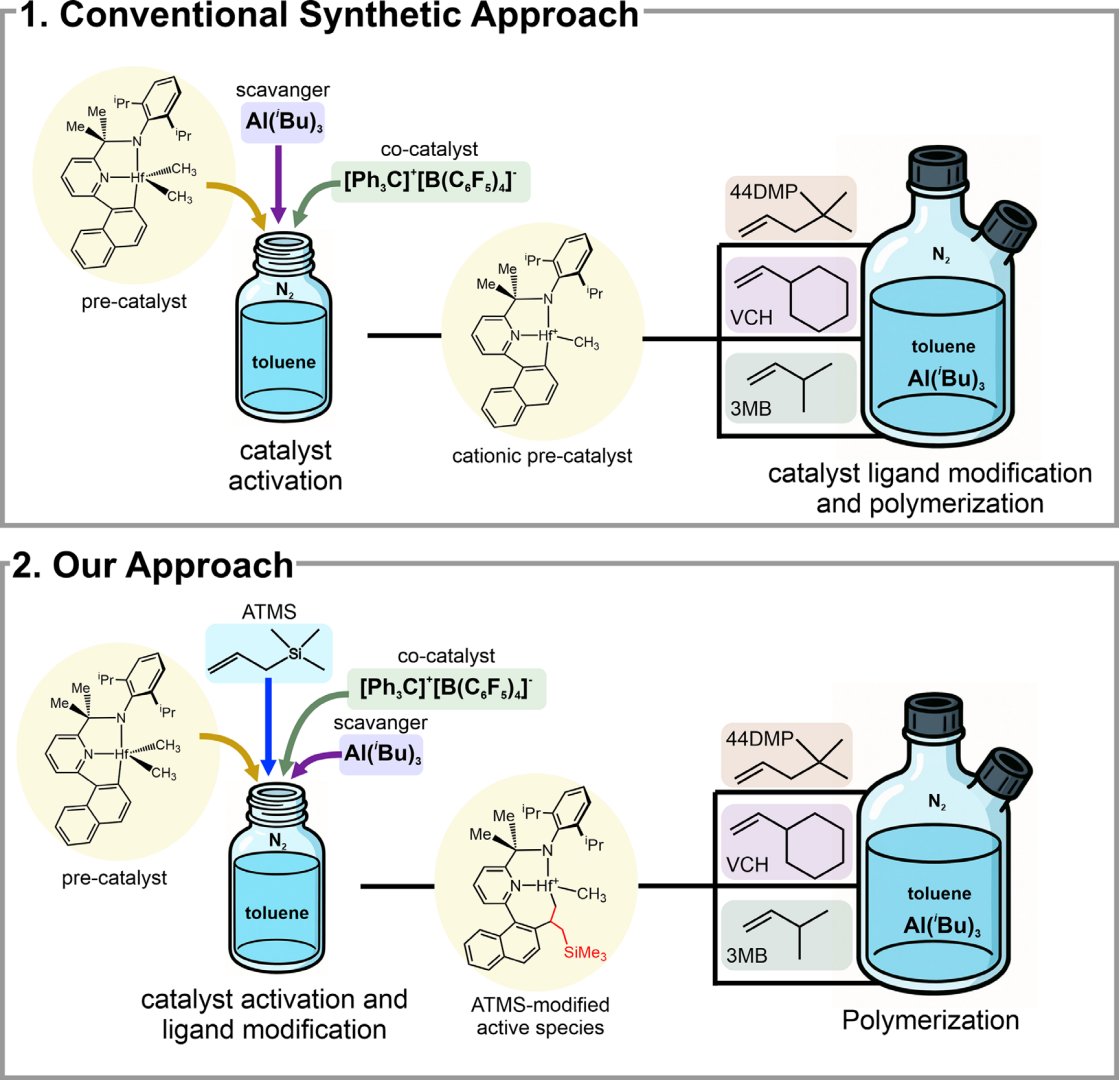
Schematic representation of the two polymerization protocols employed in this study: (1) standard polymerization procedure, in which the pyridylamido‐Hf precatalyst is directly activated by the monomer to be polymerized. (2) ATMS pre‐activation polymerization procedure, in which the precatalyst is first pre‐activated with ATMS prior to polymerization.

**TABLE 1:** Results of polymerizations of ATMS, 44DMP, VCH, and 3MB (Scheme 1) catalyzed by **1a** of Scheme 1 in standard activation procedure and by **1a** with ATMS‐pre‐activation. Monomer concentration, polymerization time (*t*_pol_), polymer yield, average turnover frequency (TOF), melting temperatures (*T*_m_) of the resulting isotactic polymers, and melting temperatures of the same polymers prepared with conventional heterogeneous ZN catalysts taken from literature (*T*_m_^Ref^).^a^

Entries	Monomers	Polymers^b^	Monomer (mol·L^−1^)	*t*_pol_ (min)	Yield (mg)	TOF (h^−1^)^c^	*T*_m_ (°C)^d^	*T*_m_ (°C)^e^ Refs.
1	ATMS	iPATMS	0.36	5	450	3425	308 (This work)	305 [30, 31]
2	44DMP	iP44DMP	0.39	60	—	—	—	>400 [39]
3	44DMP	iP44DMP*	0.39	60	230	170	422 (This work)	
4	VCH	iPVCH	0.58	90	—	—	—	370 [11, 40]
5	VCH	iPVCH*	0.58	90	480	210	393 (This work)	
6	3MB	iP3MB	0.71	90	160	110	295 (This work)	305 [12, 41]
7	3MB	iP3MB*	0.71	90	310	213	300 (This work)	

^a^ 13.8 µmol precatalyst, 18.5 µmol co‐catalyst [Ph_3_C]^+^[B(C_6_F_5_)_4_]^−^, 0.50 mmol Al(*^i^* Bu)_3_, 800 rpm, 60 min of solvent (toulene)/monomer/scavenger contact, [B]/[Hf] = 1.3, *T*_pol_ = 25°C.

^b^ Samples marked with * have been obtained with ATMS‐pre‐activation of the pyridylamido complex (2 in Scheme 2). ATMS (45 µL, 20 eq with respect to the catalyst) was precontacted with the catalyst solution for 5 min prior to injection (prepolymerization step).

^c^ Average turnover frequency (TOF): mol monomer/mol Hf × h.

^d^ Highest melting temperature values of the obtained polymers in the two polymerization conditions (1 and 2 in Scheme 2).

^e^ Highest melting temperature values reported in literature for the same polymers obtained with heterogeneous ZN catalysts [11, 12, 30, 31, 39, 40, 41].

**FIGURE 1:**
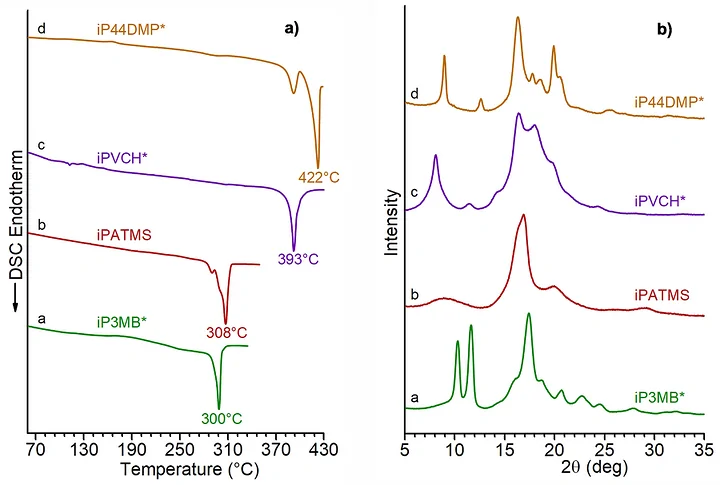
DSC heating curves (a) and X‐ray diffraction profiles (b) of the polymer samples iP3MB* (a), iPATMS (b), iPVCH* (c), and iP44DMP* (d) (Table 1) synthesized with the catalyst **1a** of Scheme 1 via ATMS‐induced pre‐activation (path b in Figure 2).

ATMS polymerization was also examined using the more isoselective (for propene polymerization) industrial catalyst **1b** of Scheme 1 (Scheme S2 and Table S2). The obtained polymer (sample iPATMS‐2) shows melting temperature and X‐ray diffraction profile similar to those of the sample prepared with **1a** (Figures S3–S5 and Table S3). Therefore, in contrast to propene polymerization, both catalyst **1a** and **1b** afforded indistinguishable iPATMS materials in terms of thermal properties and stereoregularity (Figures S1–S5 and Table S3). This indicates that, consistent with our hypothesis, catalyst **1a** did not show lower isoselectivity than **1b** in the polymerization of ATMS. This result suggests that, for such a highly sterically congested monomer, stereochemical control is predominantly dictated by monomer architecture rather than by intrinsic differences in catalyst isoselectivity.

Encouraged by this result, we checked the polymerization of 44DMP, a closely related hydrocarbon analogue of ATMS differing only by the substitution of the silicon with a carbon atom (Scheme 1). Notably, the homopolymerization of this monomer has remained essentially unexplored with homogeneous catalysts and, to the best of our knowledge, only briefly mentioned in a single study employing heterogeneous ZN systems [39]. Despite the minimal structural difference between ATMS and 44DMP, polymerization attempts of 44DMP with catalyst **1a** under the conventional activation protocol (procedure 1 in Scheme 2) were entirely unsuccessful. In particular, no polymer formation was detected when 44DMP was employed at a monomer concentration comparable to that used for ATMS polymerization (0.39 mol L^−1^ for 44DMP vs. 0.36 mol L^−1^ for ATMS) and with a substantially longer reaction time (60 min for 44DMP vs. 5 min for ATMS) (Table 1, entry 2).

Critical re‐examination of this failure by using DFT calculations (see Supporting Information for computational details) and considering the unique activation mechanism of pyridylamido‐Hf catalysts, proved decisive. As shown in Scheme S3, in these systems the catalytic active species is generated through an initial monomer insertion into the Hf─aryl bond, resulting in a modification of the ligand framework [37, 38]. We hypothesized that the lack of reactivity toward 44DMP may originate from inefficient catalyst activation, involving either inhibition of the initial insertion step or formation of a poorly propagating active species (path a in Figure 2 and Scheme S4). DFT calculations of the ligand‐modification step induced by ATMS and 44DMP in the presence of the counteranion (CA) (Figure 3) revealed that insertion of ATMS into the Hf─C_Aryl_ bond proceeds with a significantly lower activation energy than that of 44DMP (5.6 vs. 8.8 kcal/mol, Figure 3a vs. 3b, respectively).

**FIGURE 2:**
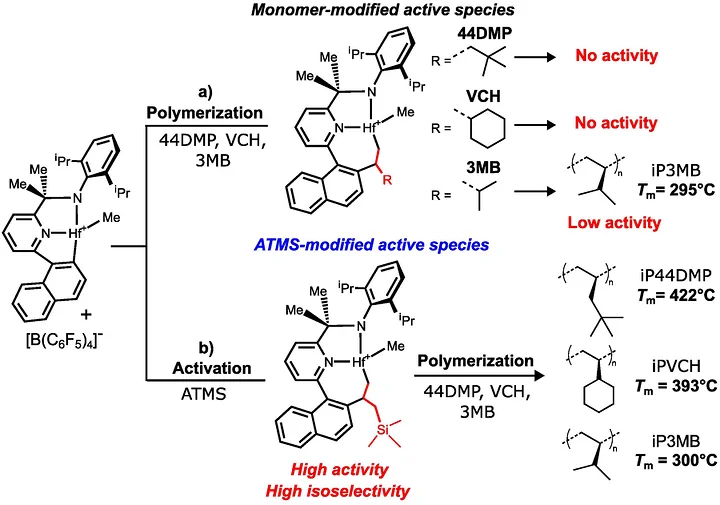
Schematic representation of the polymerization pathways examined in this work. (a) Conventional monomer‐induced activation of the pyridylamido‐Hf precatalyst by sterically demanding α‐olefins (44DMP, VCH or 3MB), resulting in no or low polymerization activity. (b) ATMS‐enabled pre‐activation strategy, in which ATMS would generate an ATMS‐modified active species exhibiting high activity and isoselectivity toward the same monomers, enabling access to high‐melting isotactic polyolefins. The proposed activation pathway b is based primarily on DFT calculationsand direct experimental evidence of the formation of modified active species is currently unavailable.

**FIGURE 3:**
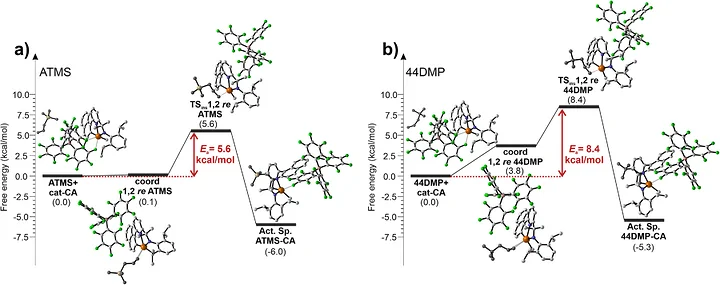
DFT free energy profiles of ligand modification of **1a** by (a) ATMS and (b) 44DMP in the presence of B(C_6_F_5_)_4_^−^ counteranion.

We, therefore, argued that ATMS, which undergoes efficient activation and polymerization (Table 1, entry 1), could be exploited not merely as a monomer, but as a “chemical trigger” to pre‐activate the Hf catalyst enabling controlled formation of highly active species capable of polymerizing otherwise inaccessible bulky α‐olefin monomers (path b in Figure 2).

On this basis, we devised an in situ monomer‐induced catalyst pre‐activation strategy (procedure 2 in Scheme 2), in which the catalyst ligand is first modified through controlled reaction with ATMS (20 equivalents relative to Hf), followed by exposure to 44DMP (path b in Figure 2) (see Supporting Information for experimental details). Under these conditions, efficient polymerization was achieved, enabling the first synthesis of isotactic poly(4,4‐dimethyl‐1‐pentene) (iP44DMP) by homogeneous catalysis (sample iP44DMP*; Table 1, entry 3). The resulting polymer sample iP44DMP* is highly crystalline (curves d of Figure 1a,b) and exhibits an extraordinarily high melting temperature of approximately 420°C (curve d of Figure 1a and Figure S6), representing the highest *T*_m_ reported for any polyolefin to date (Tables 1 and S4).

Based on the accepted activation mechanism of pyridylamido‐Hf catalysts shown in Scheme S3 and generation of active species by first monomer insertion, this intriguing result can be explained assuming that ATMS modified the ligand framework through its first insertion into the Hf─aryl bond (path b in Figure 2), generating extremely active species capable of polymerizing otherwise inaccessible bulky α‐olefin monomers, such as 44DMP. This pre‐activation mechanism is mainly based on our DFT calculations of Figure 3, even though direct experimental evidence of the generation of the modified active species is still unavailable.

As already mentioned, iP44DMP has been only briefly mentioned in an early study employing heterogeneous ZN catalysis [39], and no details of the synthesis and thermal data were reported, leaving its physical properties largely unexplored. In this context, our approach provides the first well‐defined access to this polymer using homogeneous catalysis, enabling detailed structural and thermal characterization.

Similar to iPATMS, iP44DMP is insoluble in common organic solvents, precluding solution NMR analysis. Structural characterization was, therefore, carried out by X‐ray diffraction and FT‐IR (Figures 1b and S7). X‐ray diffraction analysis revealed that the obtained sample iP44DMP* is a highly crystalline material and the diffraction profile of the as‐prepared and melt‐crystallized sample (profile d of Figure 1b and Figure S8) closely resembles that reported for the form I of iP4MP (Figure S8) [42, 43]. This suggests that iP44DMP crystallizes in a crystal structure similar to that of form I of iP4MP, confirming the isotactic microstructure of the polymer. Notably, all Bragg reflections in the diffraction profile of iP44DMP are systematically shifted to lower 2*θ* values, indicative of an expanded crystalline lattice, in agreement with the increased steric demand of the 4,4‐dimethyl‐substituted repeat unit relative to iP4MP (Figure S8).

We next extended the ATMS‐induced catalyst modification strategy to other sterically congested α‐olefins that are known to challenge homogeneous polymerization catalysis, including pyridylamido‐Hf systems [29], such as VCH and 3MB (Scheme 1). VCH and 3MB are classically polymerized using heterogeneous ZN catalysts giving interesting high melting polyolefins (Table S1) [11, 12, 41, 44, 45]. However, even in this context their polymerization remains demanding, particularly for the less reactive VCH [11, 44], for which very low TOFs are typically reported (Table S1). In homogeneous catalysis, the polymerization of VCH and 3MB has been explored with selected metallocene and post‐metallocene catalysts [29], yet the performances reported in the literature are often modest and strongly dependent on activation conditions (Table S1). Using **1a** of Scheme 1 under standard conditions (25°C, 1.5 h) (procedure 1 in Scheme 2 and path a in Figure 2), VCH proved essentially unreactive (Table 1, entry 4), whereas 3MB polymerized only sluggishly, affording iP3MB with a TOF of 160 h^−1^ (Table 1, entry 6). Strikingly, application of the ATMS‐ligand modification protocol (procedure 2 in Scheme 2 and path b in Figure 2) fundamentally altered this reactivity profile. Under identical conditions, efficient polymerization of both monomers was achieved, yielding iPVCH with a TOF of 210 h^−1^ (Table 1, entry 5) and iP3MB with an increased TOF of 213 h^−1^ (Table 1, entry 7). Notably, for both monomers these turnover frequencies exceed those reported for heterogeneous ZN systems [11, 44], with the enhancement being particularly pronounced in the case of VCH (210 h^−1^ vs. 1.33 h^−1^, Table S1).

Using VCH polymerization as a representative system, the effects of pre‐activation time, ATMS loading, and monomer concentration were also investigated (Table S5 and Figure S9). A pre‐activation time of 5 min was sufficient to reach maximum catalytic performance, while increasing the ATMS loading up to 20 equivalents progressively enhanced polymer yield. Lowering the VCH concentration to 0.30 mol L^−1^ decreased both the TOF and the melting temperature of the resulting polymer, whereas increasing it to 1.17 mol L^−1^ enhanced the TOF without significantly affecting the thermal properties. Variations in these parameters produced only limited changes in the melting temperature of iPVCH, except under the shortest pre‐activation time and lowest monomer concentration.

The successful polymerization of VCH and 3MB mediated by the ATMS‐pre‐activation of pyridylamido‐Hf catalyst has been rationalized by DFT calculations, in analogy with the case of 44DMP. The computed activation energies for ligand modification by VCH and 3MB are significantly higher than that required for ATMS (Figure S10). Consequently, the formation of the active species via VCH or 3MB insertion into the Hf─C_Aryl_ bond is thermodynamically and kinetically disfavored, resulting in a sluggish or inhibited process. This limitation is overcome by promoting active‐species formation using ATMS as a ligand‐modifier agent.

These results demonstrate that ATMS‐pre‐activation through induced ligand modification is not limited to 44DMP but represents an effective strategy for promoting the homogeneous polymerization of the structurally distinct sterically demanding α‐olefins examined in this study, including monomers that are otherwise inaccessible or only poorly polymerized under conventional activation conditions.

X‐ray diffraction (profiles a and c of Figures 1b) and FT‐IR data (Figures S11 and S12) of the obtained samples of iPVCH and iP3MB are consistent with the formation of highly isotactic polymers in both cases [11, 45, 46, 47]. The thermal analysis of both iPVCH and iP3MB obtained via ATMS‐pre‐activation protocol reveals melting temperatures higher than those reported in the literature (Table 1). In the case of iPVCH, the polymer exhibits a melting temperature of approximately 393°C (curve c of Figure 1a and Figure S13), which, to the best of our knowledge, represents the highest *T*_m_ reported to date for this material, also exceeding previously reported values for samples prepared via heterogeneous ZN catalysis [11, 40, 48] (Table 1; Tables S1 and S6). For iP3MB, the polymer obtained without ATMS activation (sample iP3MB; Table 1, entry 6) melts at approximately 295°C (Tables 1 and S7; Figure S14), whereas the sample prepared by application of the ATMS‐based pre‐activation protocol (sample iP3MB*; Table 1, entry 7) shows a higher melting temperature of approximately 300°C (curve a of Figure 1a and Figure S15; Tables 1 and S7). We highlight that this is the only case among the sterically demanding monomers investigated in which the iP3MB sample obtained by ZN catalysis exhibits a higher melting temperature than the polymer synthesized in this work using the pyridylamido‐Hf catalyst. The slightly lower *T*_m_ may reflect a lower stereoregularity, although additional microstructural effects, including possible isomerization of 3MB units, as previously reported for metallocene‐based systems [49], cannot be excluded. The reproducibility of the difference between the samples prepared with and without ATMS pre‐activation was confirmed by DSC analyses of polymers obtained from three independent polymerization runs under each condition (Table S8 and Figure S16). This modest yet systematic enhancement may indicate an increased degree of stereoregularity, confirming our hypothesis that the ATMS‐pre‐activated Hf catalyst promotes more stereoselective chain propagation. As a matter of fact, also in the case of 3MB polymerization, DFT calculations suggest that ATMS‐modified catalytic species is more isoselective than the 3MB‐modified analogue (4.2 vs. 3.5 kcal/mol, respectively, see Figure S17).

An overview of the thermal properties of the high melting polyolefins obtained in this work and catalytic performances in comparison with different standard classes of polyolefins is provided in Figure 4. It is apparent that the ATMS‐based pre‐activation strategy allows access to isotactic polyolefins with melting temperatures spanning from the regime of conventional commodity materials to values exceeding those of established high‐performance polymers such as PEEK and PTFE (Figure 4a). It is worth emphasizing that this comparison is intended solely to place the exceptionally high melting temperatures of these polyolefins in context and does not imply equivalence in terms of mechanical properties, chemical resistance, thermal stability, or processability. In particular, iPVCH and iP44DMP define an advanced class of high‐melting polyolefins, with the latter exhibiting the highest melting temperature reported to date for any polyolefin.

**FIGURE 4:**
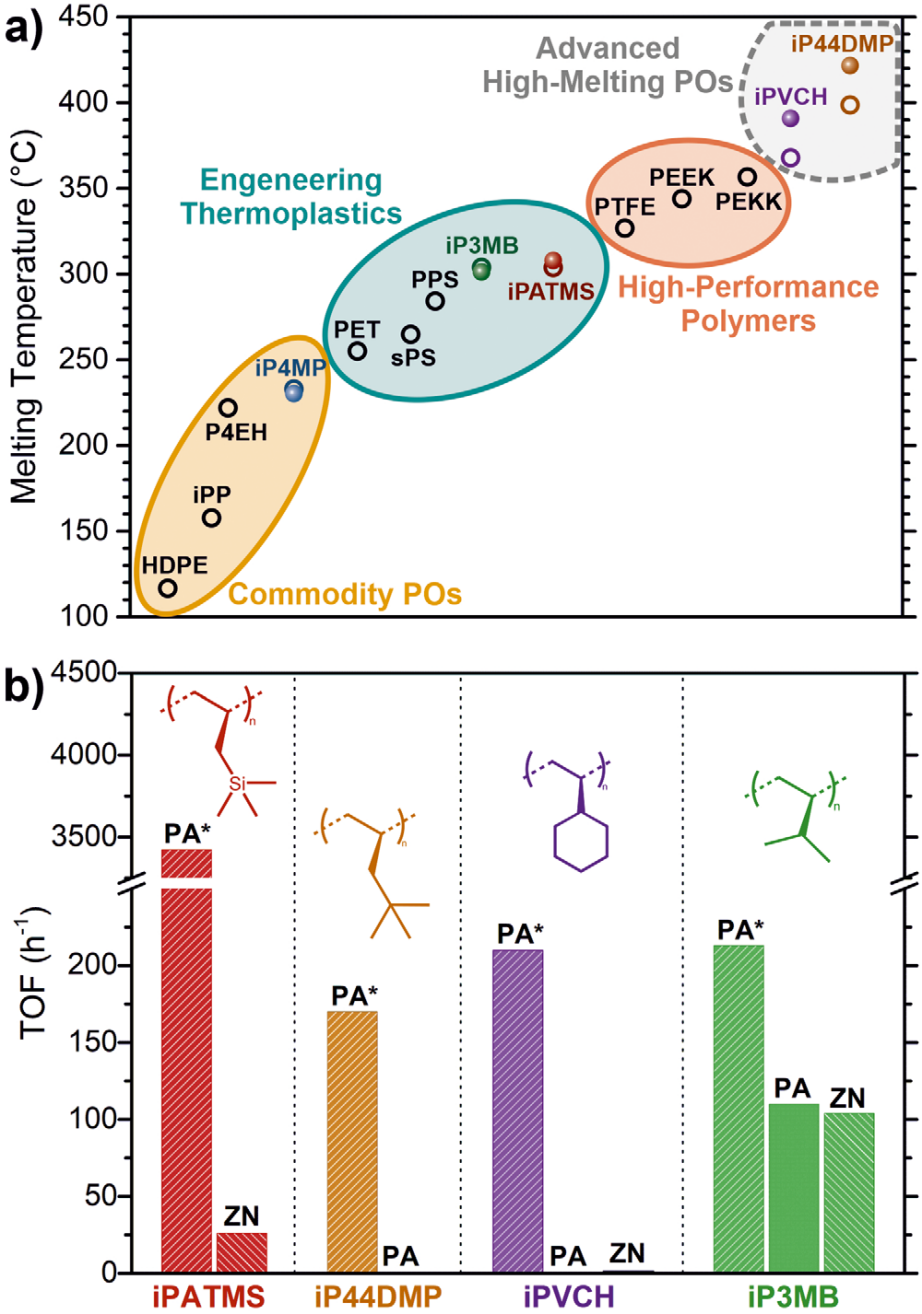
(a) Comparison of melting temperatures for different classes of polymer materials, including the polyolefins investigated in this study. For polyolefin‐based materials, open symbols denote samples obtained via ZN catalysis, while filled symbols correspond to materials synthesized in this work with the pyridylamido‐Hf catalyst (PA). (b) Turnover frequencies (TOF) for polyolefins synthesized in this study using the catalyst **1a** of Scheme 1 without (PA) and with ATMS‐induced pre‐activation (PA*), compared to literature TOF values for heterogeneous ZN systems [11, 31, 41].

To place these melting temperatures in the context of thermal stability and potential melt processability, the polymers were also analyzed by TGA (Figure S18 and Table S9). iPATMS, iP3MB, and iPVCH reach 5% mass loss at approximately 400°C, 400°C, and 420°C, respectively, whereas their melting temperatures are 308°C, 300°C, and 393°C (Table 1). Their maximum degradation rates occur at approximately 435°C, 435°C, and 450°C, respectively. By contrast, for iP44DMP, the temperature corresponding to 5% mass loss is approximately 430°C, very close to its melting temperature of about 422°C, while the maximum degradation rate is observed at approximately 460°C. This represents an important limitation of this material, whose exceptionally high melting temperature is associated with a substantially narrower thermal processing margin than those of the other polymers investigated and may, therefore, hinder its conventional melt processing. For this reason, the characterization of the mechanical properties of these ultrahigh‐melting polyolefins was not feasible, in particular, for iP44DMP. However, this limitation can be easily overcome through copolymerization of 44DMP, as well as ATMS, 3MB, or VCH, with suitably selected comonomers that may reduce the melting temperature and improve processability while retaining high thermal resistance.

To evaluate the influence of molecular weight on the melting behavior of the bulky monomers‐derived polyolefins investigated in this work, additional samples were prepared using a threefold higher Al(*^i^* Bu)_3_ loading, while keeping all other polymerization conditions unchanged, in order to promote chain transfer to aluminum (Table S10). Although the resulting samples remained insoluble, precluding direct molecular‐weight determination, they exhibited slightly lower melting temperatures and reduced supercooling values (*T*_m_−*T*_c_) compared with the corresponding samples prepared under standard conditions (Figure S19 and Table S10). These trends are consistent with the formation of shorter polymer chains. All samples retained their characteristic high‐ or ultrahigh‐melting behavior, indicating that these exceptional thermal properties are preserved under conditions expected to produce lower‐molecular‐weight polymers.

Importantly, these exceptional thermal properties are not achieved at the expense of catalytic efficiency. As shown in Figure 4b, ATMS‐induced pre‐activation consistently enhances the TOF across all monomers examined, yielding activities that surpass those reported for heterogeneous ZN catalysts, with the most pronounced effects observed for the sterically most demanding monomers. Together, these results highlight how the synthetic strategy and catalyst design reported in this study enable a simultaneous expansion of the accessible polyolefin property space while maintaining, and in some cases even enhancing, catalytic performance.

To assess the applicability of our methodology to other pyridylamido‐type catalysts, we extended the study to the polymerization of the sterically demanding monomers 44DMP, 3MB, and VCH using the industrially relevant catalyst **1b** under the same experimental conditions employed for complex **1a** (Table S11). The results obtained with **1b** closely mirror those observed for **1a**, providing direct and powerful support for the generality of the proposed strategy, apart from the higher TOF values, which are consistent with the well‐established greater catalytic activity of **1b**.

The polymers synthesized with catalyst **1b** also display thermal and structural properties very similar to those of the corresponding samples obtained with catalyst **1a**, in agreement with the behavior previously observed for iPATMS (Figure S20). To further probe the impact of our ATMS‐pre‐activation strategy, we extended the protocol to monomers that are readily polymerized by the pyridylamido‐Hf catalyst **1a** under conventional activation conditions. In particular, the polymerization of propene and 4‐methyl‐1‐pentene (4MP) was examined, even though no intrinsic reactivity limitations are known for these monomers with the catalytic system employed here. These experiments were performed to enable a direct comparison of catalytic performance with and without ATMS‐pre‐activation (Scheme S5; Tables S12 and S13).

In both cases, application of the ATMS pre‐activation protocol led to a pronounced enhancement in catalytic activity. For propene, the activity increased by approximately 30% relative to the conventionally activated system (Table S12), while a ≈15% increase was observed for 4MP polymerization (Table S13).

Since a similar TOF enhancement was also observed for 3MB polymerization, additional theoretical analyses were carried out to investigate the origin of the higher activity of the ATMS‐modified pyridylamido‐Hf species relative to the corresponding monomer‐modified analogues. We first examined whether ATMS insertion generates a less congested catalytic pocket. Steric maps of buried volume analysis (%*V*_Bur_) [50, 51] of the ATMS‐, 3MB‐, and 4MP‐modified species, however, revealed very similar ligand environments, with rather a slight increase for the ATMS‐modified species (64.2% vs. 63.4% for both the 3MB‐ and 4MP‐modified species; Figures S21 and S22). Free‐energy profiles were therefore computed for 3MB and 4MP propagation promoted by the ATMS‐ and monomer‐modified active species (Figure S23). The calculated insertion barriers were very similar, although slightly lower for the ATMS‐modified species: 17.8 versus 18.1 kcal/mol for 3MB and 7.9 versus 8.2 kcal/mol for 4MP. Given the small magnitude of these differences, they should not be regarded as the primary origin of the experimentally observed TOF enhancement.

The main contribution to the higher TOF is instead likely associated with the activation stage. Under the conventional protocol, ligand modification occurs by monomer insertion into the Hf─C_Aryl_ bond whose energetics are strictly related to the monomer bulkiness. By contrast, under the ATMS pre‐activation protocol, the catalyst is transferred into the reactor already in its propagation form. The enhanced polymerization rate is therefore mainly attributed to suppression of this activation delay, with the slightly more favorable coordination and insertion energetics providing a secondary contribution.

Notably, the iPP obtained via ATMS pre‐activation exhibits a slightly higher melting temperature (135°C) than that of the sample obtained without ATMS pre‐activation (131.5°C) (Figures S24–S27 and Table S14), analogous to the trend observed for 3MB, whereas similar melting temperatures have been obtained for samples of iP4MP (Figures S28–S31 and Table S15). As observed for iP3MB, the approximately 5°C increase in *T*_m_ detected for iPP upon ATMS pre‐activation was consistently reproduced across three independent polymerization experiments performed under each activation condition (Table S16 and Figure S32).

These observations, together with ^13^C NMR analysis (Figure S33), indicate that ATMS‐pre‐activation enhances the stereoselectivity of the catalytically active species also in propene polymerization. For 4MP the stereochemical outcome remains essentially insensitive to the nature of the initial activation event, despite the pronounced enhancement in catalytic activity. These experimental results are also consistent with the stereoselectivity predicted by DFT calculations. Indeed, an increase in Δ*G*_stereo_ is predicted for propene polymerization mediated by ATMS‐modified catalytic species compared with the propene‐modified active species (4.4 vs. 3.2 kcal/mol, Figure S34). The computed isoselectivity of the 4MP‐inserted pyridylamido‐Hf species is already very high (Δ*G*_stereo_ = 4.8 kcal/mol, Figure S35), in agreement with the high isotacticity of the obtained P4MP ([*m*]^4^ > 98%, Figure S36), and therefore any further increase in stereoselectivity upon ATMS preactivation is hardly appreciable.

It is worth noting that the ^13^C NMR spectra of both iPP and iP4MP obtained using the ATMS pre‐activation strategy show very low‐intensity signals attributable to ATMS‐derived units (Figures S33 and S36). Their contents, estimated from the ^1^H NMR spectra, were 0.2 and 0.1 mol% for iPP and iP4MP, respectively (Figures S37 and S38). Considering that the melting temperatures of these samples are equal to or higher than those of the corresponding polymers prepared under conventional conditions, the ATMS‐derived units are likely located predominantly near the chain ends rather than randomly distributed along the polymer backbone as comonomeric units.

## 3 Conclusion

Pyridylamido‐Hf catalysts were originally discovered through high‐throughput screening for the industrial production of advanced polyolefins. Our study demonstrates that a rational redesign of the catalyst active site can substantially enhance the performance of this catalyst family and unlock previously inaccessible polymer structures. We proved that ATMS can serve as an effective pre‐activator of the pyridylamido‐Hf catalysts, enabling controlled formation of highly active species and unlocking the homogeneous polymerization of sterically demanding α‐olefins. This, in turn, allows the production of extremely high melting temperature polyolefins that cannot be efficiently synthesized with either industrial heterogeneous ZN catalysts or standard homogeneous molecular catalysis.

This ATMS‐pre‐activation approach allows the first homogeneous catalyzed synthesis of iP44DMP, exhibiting an unprecedented melting temperature of ca. 420°C, as well as highly crystalline iPVCH and iP3MB displaying record or enhanced thermal properties. Importantly, these exceptional melting behaviors are achieved alongside enhanced catalytic performance, with turnover frequencies surpassing those reported for heterogeneous ZN systems, particularly for the most sterically congested monomers.

The exceptionally high melting temperatures of these materials, however, reduce their processability window. This limitation can be easily overcome through copolymerization of 44DMP, ATMS, 3MB, or VCH with suitably selected comonomers that may reduce the melting temperature offering a viable route to improve processability while retaining high thermal resistance.

DFT calculations provide mechanistic insight into this behavior revealing that the activation mechanism based on ligand‐modification by the monomer is disfavored in the presence of bulky monomers (VCH, 3MB, and 44DMP). Such limitation is exceeded when ATMS is employed as ligand modifying agent, readily generating a propagating active species. In addition to explaining the higher catalytic activity observed with ATMS, theoretical investigations also indicate that the ATMS‐modified active species exhibit enhanced isoselectivity. Extension to propene and 4‐methyl‐1‐pentene has further confirmed that ATMS‐pre‐activation can enhance catalytic efficiency and, in selected cases, stereoselectivity. We are aware that the proposed activation pathway (b in Figure 2) involving ligand modification by ATMS insertion is based primarily on DFT calculations. We are therefore pursuing complementary experimental studies aimed at directly identifying and characterizing the proposed active species [37, 38, 52]. These investigations are currently ongoing, and their results will be reported in due course.

This work demonstrates that fine tuning of the active species through monomer‐induced modification of pyridylamido‐Hf catalysts represents an effective strategy for accessing advanced polyolefins with exceptionally high‐melting temperatures.

## Author Contributions

**Fabio De Stefano**: investigation and writing – original draft. **Olga D'Anania**: investigation and writing – original draft. **Giovanni Talarico**: data curation and supervision. **Claudio De Rosa**: conceptualization, writing – review and editing, and supervision.

## Conflicts of Interest

The authors declare no conflicts of interest.

## Supporting information



The Supporting Information file includes the experimental details of polymerization procedures (standard and ATMS pre‐activation protocols) and characterization techniques; additional polymerization and characterization data for iPATMS, iP44DMP, iPVCH, iP3MB, iPP, and iP4MP, including WAXS, FT‐IR, DSC, TGA, ^13^C NMR, and ^1^H NMR data; computational details and additional DFT results. The authors have also cited additional references within the file [53, 54, 55, 56, 57, 58, 59, 60, 61, 62, 63, 64, 65].**Supporting File**: anie74884‐sup‐0001‐SuppMat.pdf.

## Data Availability

The data that support the findings of this study are available on request from the corresponding author. The data are not publicly available due to privacy or ethical restrictions.
