# Supplementary material for: Fine Tuning of Catalyst Active Sites to Access Advanced Ultrahigh‐Melting Polyolefins
Source: Angew Chem Int Ed Engl. 2026 Sep 21;65(45):e9519061. doi: 10.1002/anie.9519061 (PMC13725554; doi:10.1002/anie.9519061)
Supplement: Supplementary file 1 [file ANIE-65-e9519061-s001.pdf]

Supporting Information

Fine Tuning of Catalyst Active Sites to Access Advanced Ultra-High-Melting Polyolefins

Fabio De Stefano,[a] Olga D'Anania,[a,b] Giovanni Talarico,[a,b] and Claudio De Rosa*[a]

[a]Dipartimento di Scienze Chimiche, Università di Napoli Federico II, Complesso Monte S. Angelo, Via Cintia, I-80126 Napoli, Italy

[b]Scuola Superiore Meridionale, Largo San Marcellino 10, 80138 Napoli, Italy

Index

1) General Materials and Methods ... 3

1.1 Polymerization ... 3

1.2 Characterization ... 4

1.3 Computational details ... 6

2) Additional Experimental Data and Computational Results ... 7

2.1 Additional Polymerization Data of ATMS and Characterization of iPATMS ... 9

2.2 Pyridylamido-Hf Activation Path ... 13

2.3 Additional characterization data of iP44DMP ... 15

2.4 Effect of pre-activation variables and monomer concentration on catalytic performances ... 17

2.5 Comparative Computational Results for ligand modification by the investigated bulky monomers ... 19

2.6 Additional Characterization data of iPVCH and iP3MB ... 20

2.7 Thermogravimetric Analysis of Bulky-Monomer-Derived Polyolefins ... 25

2.8 Effect of Increased TIBA Loading on Polymerization Performance and Thermal Properties ... 26

2.9 Additional Data of Bulky Monomers Polymerization Promoted by Catalyst 1b ... 28

2.10 Additional Data of Propene and 4-Methyl-1-Pentene Polymerization and Computational Data on 3MB and 4MP Propagation ... 30

3) References ... 45

1) General Materials and Methods

1.1) Polymerization

All manipulations involving air- and/or moisture-sensitive substances were carried out under an inert atmosphere using standard Schlenk and glovebox techniques.

As described in the main text, polymer syntheses were performed following two distinct protocols, shown in Scheme 2. The first corresponds to a conventional polymerization method, hereafter referred to as the *standard polymerization procedure*, in which the precatalyst is directly activated in the presence of the monomer to be polymerized (procedure 1 in the Scheme 2). The second protocol differs in that the precatalyst is pre-activated with allyltrimethylsilane (ATMS) prior to injection of the resulting active species into the polymerization medium. This latter method is hereafter referred to as the *ATMS pre-activation polymerization procedure* (procedure 2 in the Scheme 2). For clarity, the Scheme 2 illustrates only the cases of ATMS, 4,4-dimethyl-1-pentene (44DMP), vinylcyclohexane (VCH), and 3-methyl-1-butene (3MB) as representative monomers, while 4-methyl-1-pentene (4MP) and propene are omitted.

<u>1) Standard polymerization procedure.</u>

Polymerizations were carried out at 25 °C in a 80 mL Pyrex reactor equipped with a magnetic stirrer. In a typical experiment, the reactor was initially charged with toluene, $Al(^{i}Bu)_3$ (15 wt% solution in toluene), and the liquid monomer. The resulting mixture was allowed to equilibrate at the reaction temperature for 1 h under stirring, in order to ensure thermal stabilization and effective scavenging of residual impurities.

After this equilibration period, polymerization was initiated by injection of a toluene solution containing the pyridylamido hafnium precatalyst (≈13.8 µmol) and the boron activator (≈18.5 µmol). The B/Hf molar ratio was maintained at about 1.3. The reaction was allowed to proceed for the desired time (see Table 1) under constant stirring (800 rpm).

The polymerization was quenched by addition of methanol acidified with concentrated aqueous HCl. The resulting polymer was isolated by filtration, thoroughly washed with methanol, and dried under vacuum at 40 °C to constant weight. This procedure was applied to the homopolymerization of allyltrimethylsilane, 4,4-dimethyl-1-pentene, vinylcyclohexane, 3-methyl-1-butene and 4-methyl-1-pentene. In the case of propene homopolymerization, the reactor was charged with propene gas at 1 bar and allowed to equilibrate for 1 h to ensure saturation prior to catalyst injection.

<u>2) ATMS pre-activation polymerization procedure.</u>

Polymerizations were carried out at 25 °C in an 80 mL Pyrex reactor equipped with a magnetic stirrer. In a typical experiment, the reactor was initially charged with toluene, $Al(^{i}Bu)_3$ (15 wt% solution in toluene), and the liquid monomer. The resulting mixture was allowed to equilibrate at the reaction temperature for 1 h under stirring, in order to ensure thermal stabilization and effective scavenging of residual impurities.

In parallel, a toluene solution of the pyridylamido hafnium precatalyst (≈13.8 µmol) and the boron activator (≈18.5 µmol, B/Hf ≈ 1.3) was pre-contacted with allyltrimethylsilane (ATMS,

45 µL, corresponding to 20 equivalents with respect to the precatalyst) for 5 min at room temperature, allowing formation of the ATMS-modified active species (pre-activation step).
After this pre-activation period, the resulting solution was injected into the reactor to initiate polymerization. The reaction was allowed to proceed for the desired time (see Table 1) under constant stirring (800 rpm).
Polymerization was quenched by addition of methanol acidified with concentrated aqueous HCl. The resulting polymer was isolated by filtration, thoroughly washed with methanol, and dried under vacuum at 40 °C to constant weight. This procedure was applied to the homopolymerization of 4,4-dimethyl-1-pentene, vinylcyclohexane, 3-methyl-1-butene and 4-methyl-1-pentene. In the case of propene homopolymerization, the reactor was charged with propene gas at 1 bar and allowed to equilibrate for 1 h to ensure saturation prior to catalyst injection.
For the homopolymerization of 4-methyl-1-pentene and propene, additional experiments were performed using a lower ATMS loading, corresponding to 5 equivalents with respect to the precatalyst, in addition to the standard 20 equivalents employed in the pre-activation step.

1.2) Characterization

Nuclear magnetic resonance (NMR) spectra were recorded on a Bruker DPX-400 spectrometer operating in the Fourier transform mode at 120 °C at 100.61 MHz. The carbon spectra were acquired with a 90° pulse and 15 seconds of delay between pulses and CPD (WALTZ 16) to remove ^{1}H-^{13}C coupling. About 1500-3000 transients were stored in 32K data points using a spectral window of 6000 Hz. Mestrenova software by Mestrelab Research S.L. (version 14.1.2) was used for data evaluation. Solution-state NMR analysis was feasible only for isotactic polypropylene and isotactic poly(4-methyl-1-pentene) samples, as all other polyolefins investigated in this study were insoluble in common organic solvents.
The molar fractions of ATMS units in the iPP and iP4MP samples synthesized with the ATMS-preactivation strategy, were determined by ^{1}H NMR and calculated according to Equations 1-5.[S1]
Equation 1 sets the value of a proton belonging to the methyl group of ATMS unit and was used as a reference for the ATMS content evaluation.
Equations 2 and 4 set the value of a proton belonging to a propene and 4-methyl-1-pentene units, respectively. To define the value of a proton belonging to the monomeric unit of interest (propene or 4-methyl-1-pentene), it is necessary to subtract the contribute of the aliphatic protons of the ATMS units (*i.e.* 5 protons besides those belonging to methyl groups linked to the silicon atom) from the signals counting all the aliphatic protons (0.5-1.9 ppm) and then divide it by the number of protons in each monomeric unit (*i.e*. 6 for propene and 11 for 4-methyl-1-pentene). The concentration of ATMS is calculated as mol% by using Equations 3 and 5.

Eq. 1 $$H_{Me-ATMS} = \frac{H_{(0.0-0.3ppm)}}{9}$$

Eq. 2 $$H_P = \frac{H_{(0.5-1.9ppm)} - 5H_{Me-ATMS}}{6}$$

Eq. 3 $$[ATMS] = \frac{H_{Me-ATMS}}{H_{Me-ATMS} + H_P} * 100$$

Eq. 4 $$H_{4MP} = \frac{H_{(0.5-1.9ppm)} - 5H_{Me-ATMS}}{11}$$

Eq. 5 $$[ATMS] = \frac{H_{Me-ATMS}}{H_{Me-ATMS} + H_{4MP}} * 100$$

The average molecular masses and the polydispersity were determined by gel permeation chromatography (GPC), using a Polymer Laboratories GPC220 apparatus equipped with a differential refractive index (RI) detector and a Viscotek 220R viscometer (Agilent Company, Santa Clara, CA, USA), on polymer solutions in 1,2,4-trichlorobenzene at 135 °C. GPC analysis was feasible only for isotactic polypropylene and isotactic poly(4-methyl-1-pentene) samples, as all other polyolefins investigated in this study were insoluble under the analysis conditions.

Differential scanning calorimetry (DSC) measurements were performed using a Mettler DSC822 instrument operating under a nitrogen atmosphere. Polymer samples (typically ca. 5 mg) were sealed in aluminum pans.

The temperature program was adapted to the thermal stability and melting behavior of each polyolefin investigated. Specifically, measurements were conducted over the following temperature ranges: 25-180 °C for isotactic polypropylene, 25-270 °C for isotactic poly(4-methyl-1-pentene), 25-330 °C for isotactic poly(3-methyl-1-butene), 25-350 °C for isotactic poly(allyltrimethylsilane), and 25-430 °C for isotactic poly(4,4-dimethyl-1-pentene) and isotactic poly(vinylcyclohexane).

For all samples, DSC analyses consisted of a first heating scan performed on the as-polymerized material, followed by a cooling scan and a second heating scan on the melt-crystallized sample. All scans were recorded at a constant heating and cooling rate of 10 °C min^{-1}. The melting temperatures of high-melting polyolefins discussed in the main text are consistently taken from the first heating scans (see Table 1), which most accurately reflect the thermal properties of the as-polymerized materials.

X-ray diffraction patterns have been recorded at room temperature with Ni filtered Cu Kα radiation (wavelength λ=0.15418 nm). The powder diffraction profiles have been obtained with an automatic Empyrean diffractometer (Marvern-PANalytical) operating in the reflection geometry with continuous scans of the 2θ angle and scanning rate of 0.02 degree/s. Diffraction measurements were recorded on all as-polymerized polymer samples.

FT-IR spectra were collected with a JASCO FT-IR spectrometer (model 4700LE, with resolution 4 cm^{-1}, scan number 128). The range of scanning wavenumber was selected from 4000 to 400 cm^{-1}.

Thermogravimetric analysis (TGA) was carried using a Perkin-Elmer (Waltham, MA, USA)

TA4000 instrument at a heating rate of 10 °C min^{-1} under a flowing N_2 atmosphere in the temperature range 150–900 °C.

1.3) Computational Details

<u>DFT calculations</u>

The structures of all the intermediates and transition states (TSs) have been optimized through employment of the Gaussian16 set of programs.[S2] The B3LYP functional[S3, S4] has been used in conjunction with two layers of basis sets. The standard polarized split-valence basis set of Ahlrichs et al. (SVP)[S5] has been employed for H, C, N, and Si atoms, while the LANL2DZ basis and Effective Core Potential (ECP)[S6] have been used for the Hf metal center. The nature of the stationary points has been confirmed through a vibrational analysis and by checking the presence of zero for intermediates and only one imaginary frequency for TSs. This analysis has been also used for the computation of zero-point energies and thermal (enthalpy and entropy) corrections (298.15 K, 1 bar). Refined electronic energies have been calculuted by performing single point calculations with the same functional, but a larger TZVP basis set[S7] for H, C, N and Si and the SDD basis augmented with a f function with exponent 0.5 and pseudopotential[S8] for Hf. The dispersion corrections (EmpiricalDispersion=D3)[S9] and solvation contribution (toluene) through the Polarizable Continuum Model (PCM)[S10] have been included. Refined electronic energies have been added to the thermal corrections computed at the B3LYP/SVP/LANL2DZ level to obtain the free energy (ΔG) values. In the comparison of Gibbs free energies along the reaction profiles, where unimolecular species (coordination intermediates, insertion transition states, and products) were referenced to two separated optimized species, translational entropies were corrected using the Martin correction[S11] as modified for olefin polymerization catalysis.[S12] The employed computational methodology reported in the main text has been already used in benchmark studies on the stereoselectivity of propene polymerization and diastereoselectivity in α,ω-dienes cyclopolymertization promoted by *ansa*-metallocene and nonmetallocene complexes.[S13, S14]

The steric maps of Buried Volume (%V_{Bur})[S15,S16] have been computed by using the SambVca 2.1 program. The sphere, centered on the Hf atom, was set to have a radius of 4.5 Å.

2) Additional Experimental Data and Computational Results

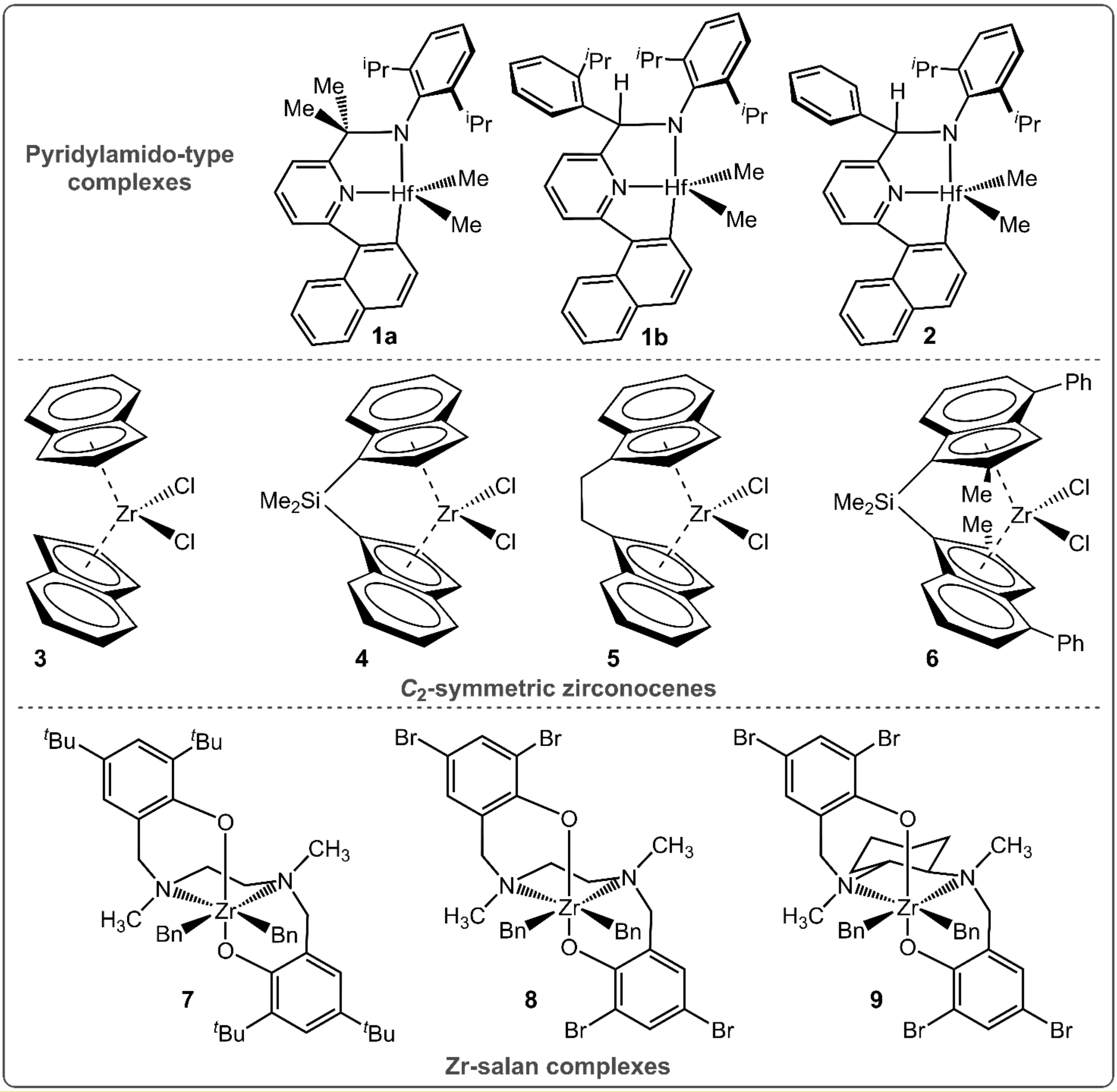

Scheme S1. Homogeneous catalysts reported in literature for the polymerization of bulky monomers[S17-S21] (Table S1).

Table S1. Results of polymerizations of ATMS, 44DMP, VCH and 3MB catalyzed by heterogenous ZN catalysts and homogenous catalysts reported in the Scheme S1 taken from literature.[S14-S20] Used co-catalysts, monomer concentration, polymerization temperature (T_{pol}), average turnover frequency (TOF) and melting temperatures (T_m) of the isotactic polymers are reported in comparison with data obtained in this work with catalysts **1a** and **1b** of Schemes 1 and S1.

Sample	Catalyst	Cocatalyst	[Monomer] (mol·L^{-1})	T_{pol} (°C)	TOF (h^{-1}) [a]	T_m (°C) [c]	*Ref.*
iPATMS	**1a**	**$[Ph_3C]^+[B(C_6F_5)_4]^-$**	**0.36**	**25**	**3425.00**	**308**	**This work**
	**1b**	**$[Ph_3C]^+[B(C_6F_5)_4]^-$**	**0.36**	**25**	**8372.00**	**308**	**This work**
	**$TiCl_4$**	$Al(C_2H_5)_3$	-	85	26.36[b]	305	S17, S21
	**3**	MAO	-	22	466.00	-(atactic)	S17
	**4**	MAO	-	50	6799.00	267	S17
	**5**	MAO	0.95	50	1208.00	281	S18
iP44DMP	**1a**	**$[Ph_3C]^+[B(C_6F_5)_4]^-$**	**0.39**	**25**	**170.00**	**422**	**This work**
	**1b**	**$[Ph_3C]^+[B(C_6F_5)_4]^-$**	**0.36**	**25**	**306.00**	**423**	**This work**
iPVCH	**1a**	**$[Ph_3C]^+[B(C_6F_5)_4]^-$**	**0.58**	**25**	**210.00**	**393**	**This work**
	**1b**	**$[Ph_3C]^+[B(C_6F_5)_4]^-$**	**0.58**	**25**	**680.00**	**394**	**This work**
	**$TiCl_3$**	$Al(i\text{-}C_4H_9)_3$	4.00	30	1.33[b]	370	S22
	**7**	$B(C_6F_5)_3$	-	-	0.04	381	S19
	**8**	$B(C_6F_5)_3$	-	-	36.00	369	S19
	**9**	$B(C_6F_5)_3$	-	-	26.00	371	S19
	**6**	MAO	1.45	20	42.00	-	S20
iP3MB	**1a**	**$[Ph_3C]^+[B(C_6F_5)_4]^-$**	**0.71**	**25**	**213.00**	**300**	**This work**
	**1b**	**$[Ph_3C]^+[B(C_6F_5)_4]^-$**	**0.71**	**25**	**427.00**	**302**	**This work**
	**$TiCl_3$**	$Al(CH_3)_3$	5.35	60	104.00[b]	305	S23
	**2**	$[Ph_3C]^+[B(C_6F_5)_4]^-$	2.10	20	50.00	-	S20
	**6**	MAO	2.10	20	152.00	-	S20

[a] Average turnover frequency (TOF): mol monomer/mol Hf × h.
[b] TOFs reported for heterogeneous ZN catalysts have been calculated by considering 10% of the total Ti centers as active sites.
[c] Evaluated from DSC measurements recorded at 10°C/min.

2.1) Additional Polymerization Data of ATMS and Characterization of iPATMS

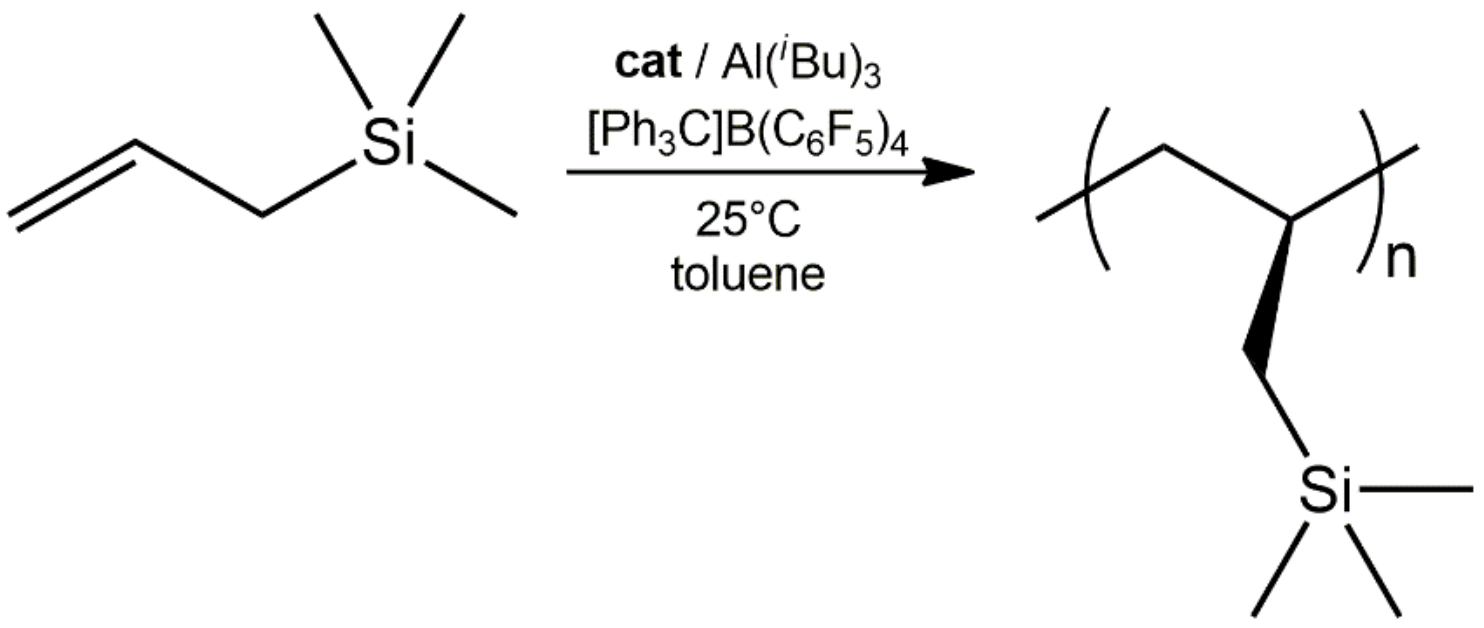

Scheme S2. Polymerization of ATMS catalyzed by pyridylamido-Hf complexes **1a** and **1b** of Scheme 1. Complex **1a**, employed throughout this work (see Scheme 1), was used for the polymerization of all sterically demanding monomers investigated, whereas complex **1b** represents an industrially implemented pyridylamido-Hf catalyst.

Table S2. Results of ATMS polymerization catalyzed by pyridylamido-Hf complexes **1a** and **1b** reported in Scheme 1.[a]

Entry	Sample	cat	[M] (mol·L^{-1})	t_{pol} (min)	Yield (mg)	TOF (h^{-1})
1	iPATMS	**1a**	0.36	5	450	3425
2	iPATMS-2	**1b**	0.36	5	1100	8372

[a] 13.8 µmol precat., 18.5 µmol co-cat., 0.50 mmol Al(^{i}Bu)$_3$, 800 rpm, 60 min of solvent (toulene)/monomer/scavenger precontact, [B]/[Hf]=1.3, T_{pol}=25 °C.

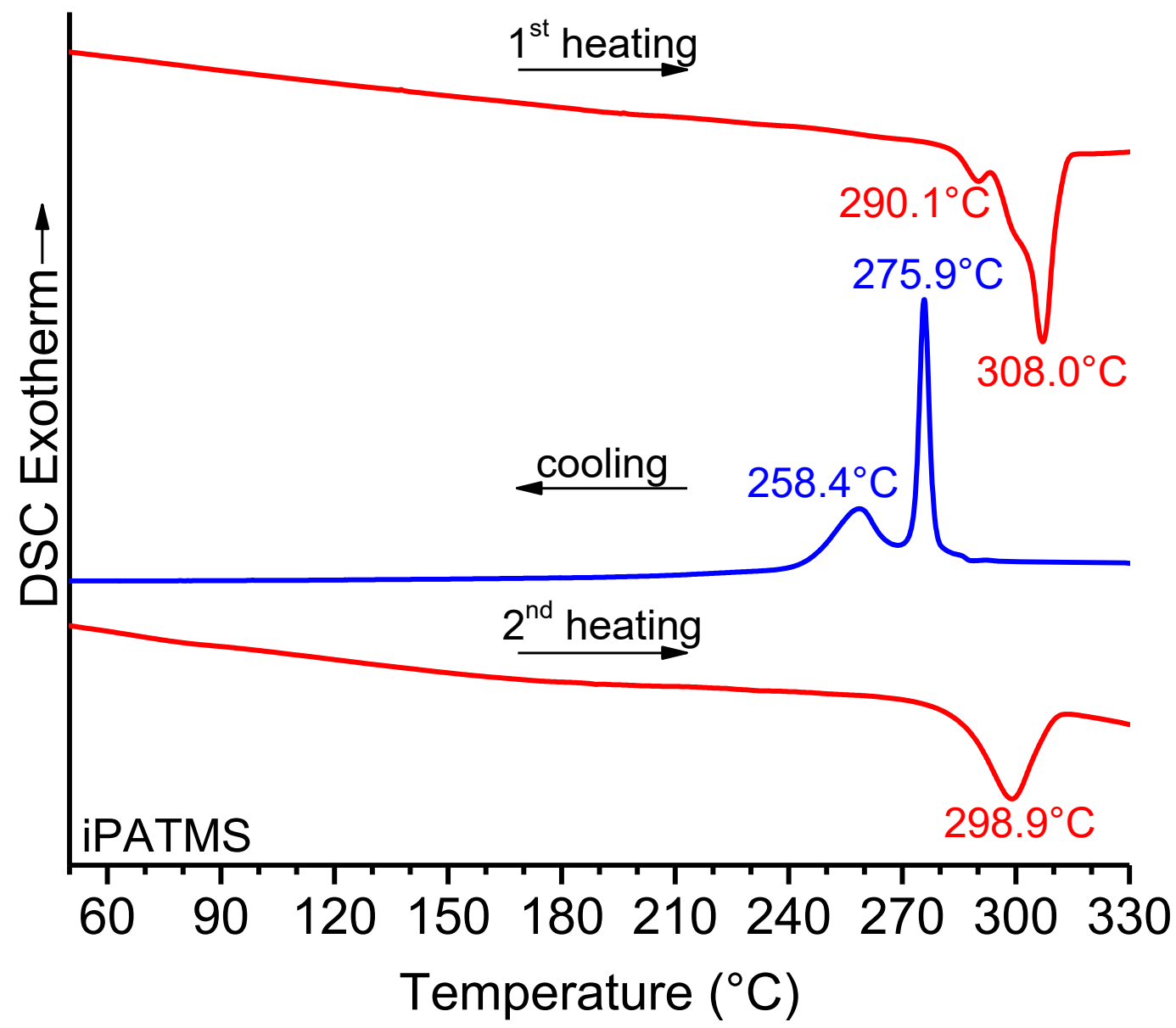

Figure S1. DSC thermograms of the sample iPATMS (entry 1 of Table 1 and Table S1) synthesized using the pyridylamido-Hf complex **1a** shown in the Scheme 1, displaying the first heating, cooling, and successive second heating scans recorded at a scan rate of 10 °C min^{-1}.

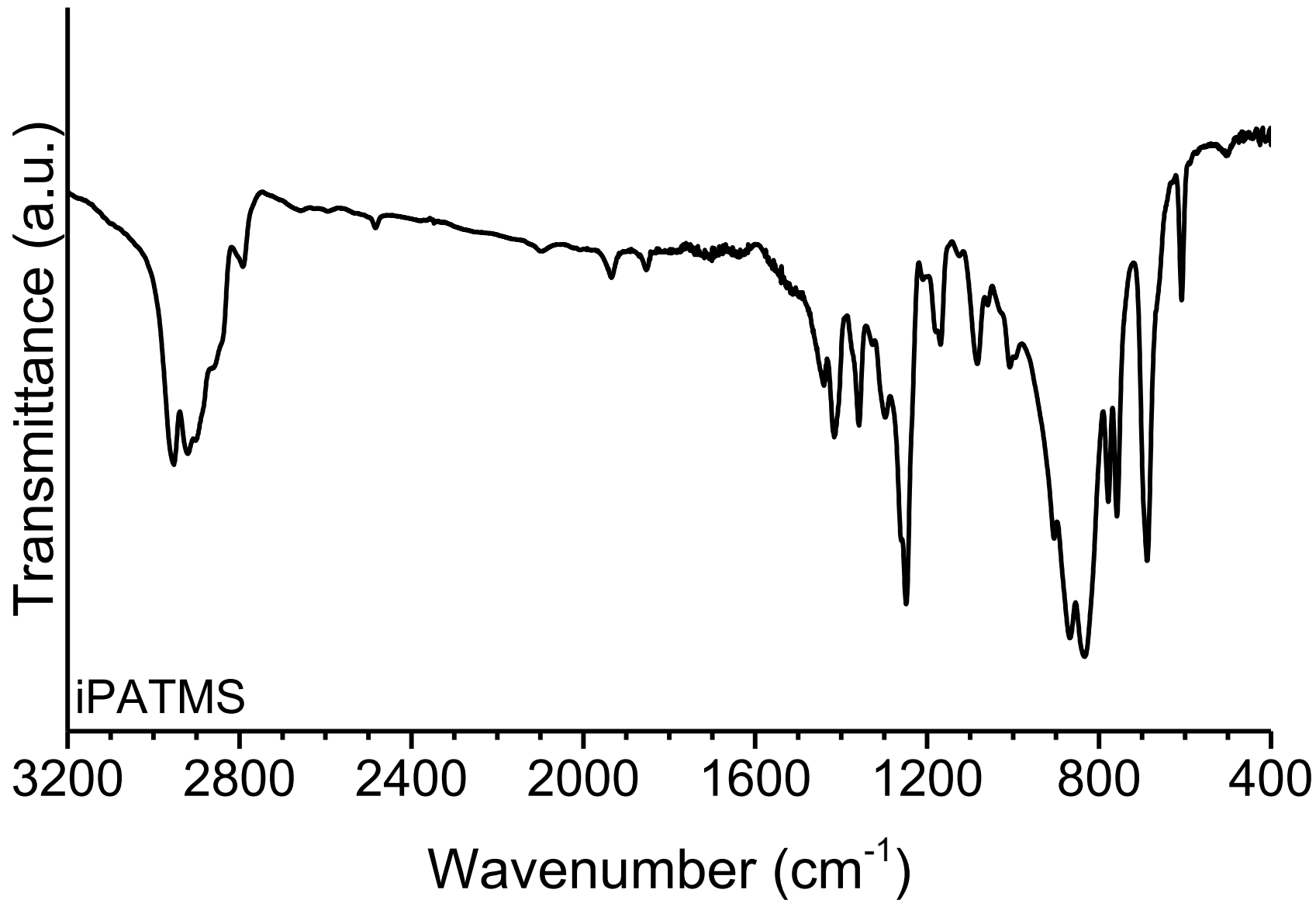

Figure S2. FT-IR spectra of the sample iPATMS (entry 1 of Table 1 and Table S1) synthesized using the pyridylamido-Hf complex **1a** shown in Scheme 1.

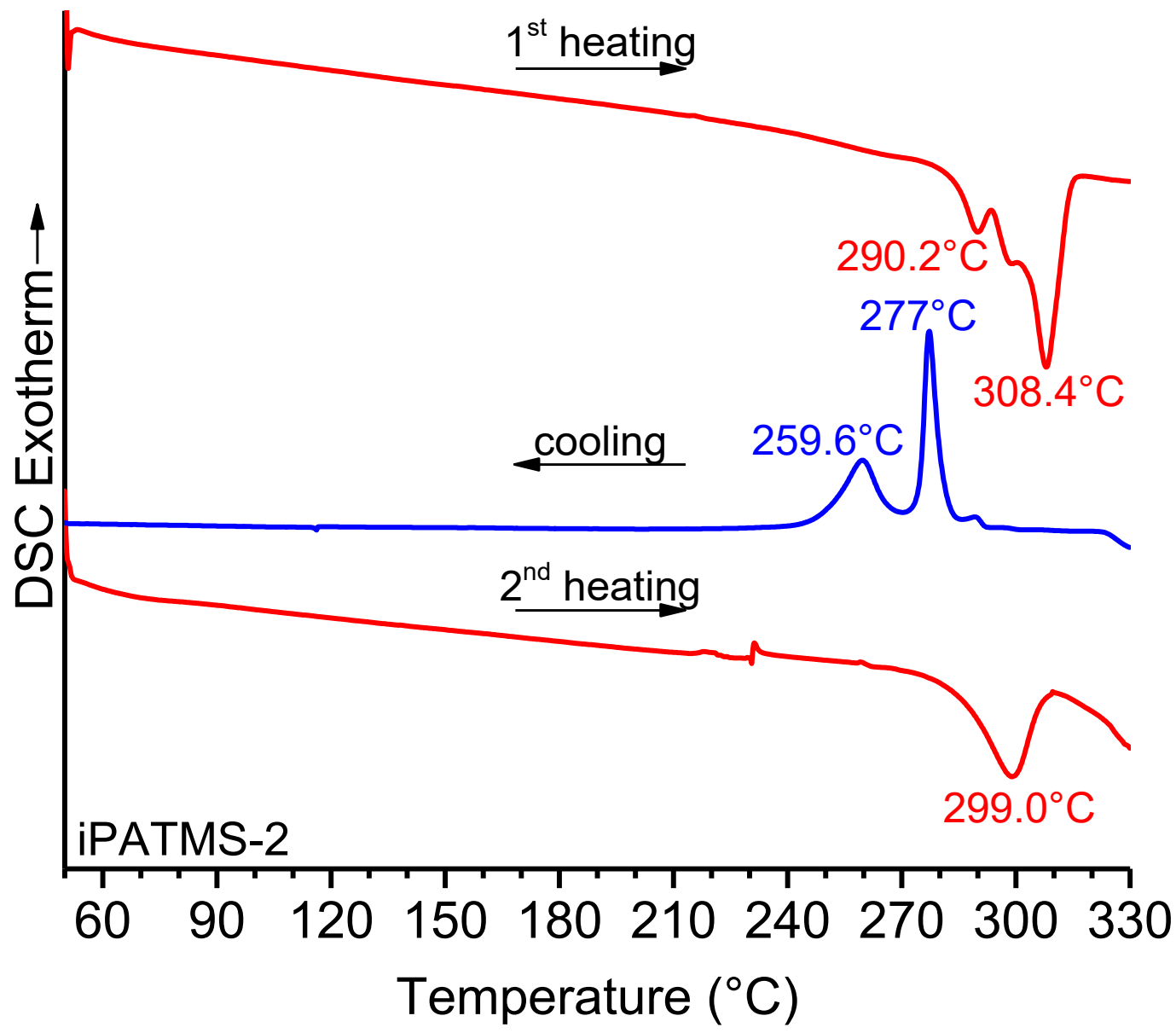

Figure S3. DSC thermograms of the sample iPATMS-2 synthesized using the industrial implemented pyridylamido-Hf complex **1b** shown in the Scheme 1, displaying the first heating, cooling, and successive second heating scans recorded at a scan rate of 10 °C min^{-1}.

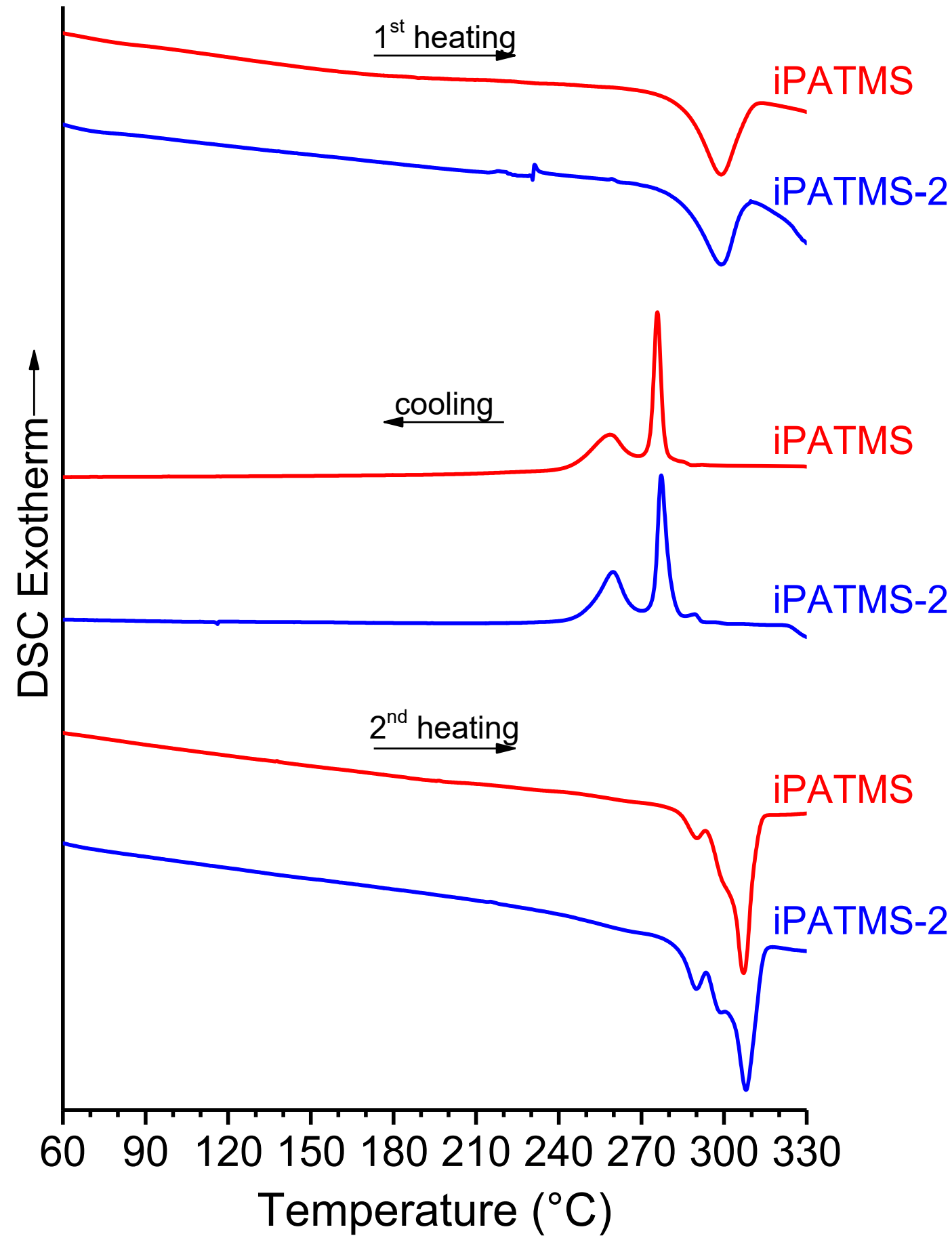

Figure S4. Comparison of the first heating, cooling, and successive second heating DSC curves of iPATMS samples synthesized using the pyridylamido-Hf complex **1a** (sample iPATMS) and the industrial catalyst variant **1b** (sample iPATMS-2) shown in Scheme 1, recorded at a scan rate of 10 °C min^{-1}.

Table S3. Melting temperature recorded during first heating of as-prepared samples (T_m^{I}), crystallization temperature recorded upon cooling from the melt (T_c) and melting temperature (T_m^{II}) recorded during second heating after melt-crystallization of samples of iPATMS prepared with catalysts **1a** and **1b** of Scheme 1 extracted from DSC curves of Figures S1 and S3.

Sample	cat	T_m^{I} (°C)	T_c (°C)	T_m^{II} (°C)	ΔH_m^{I} (J·g^{-1})	ΔH_c (J·g^{-1})	ΔH_m^{II} (J·g^{-1})
**iPATMS**	**1a**	290.2, 308.0	258.4, 275.9	298.9	44.3	39.2	27.0
**iPATMS-2**	**1b**	290.2, 308.4	259.6, 277.0	299.0	37.7	34.7	22.3

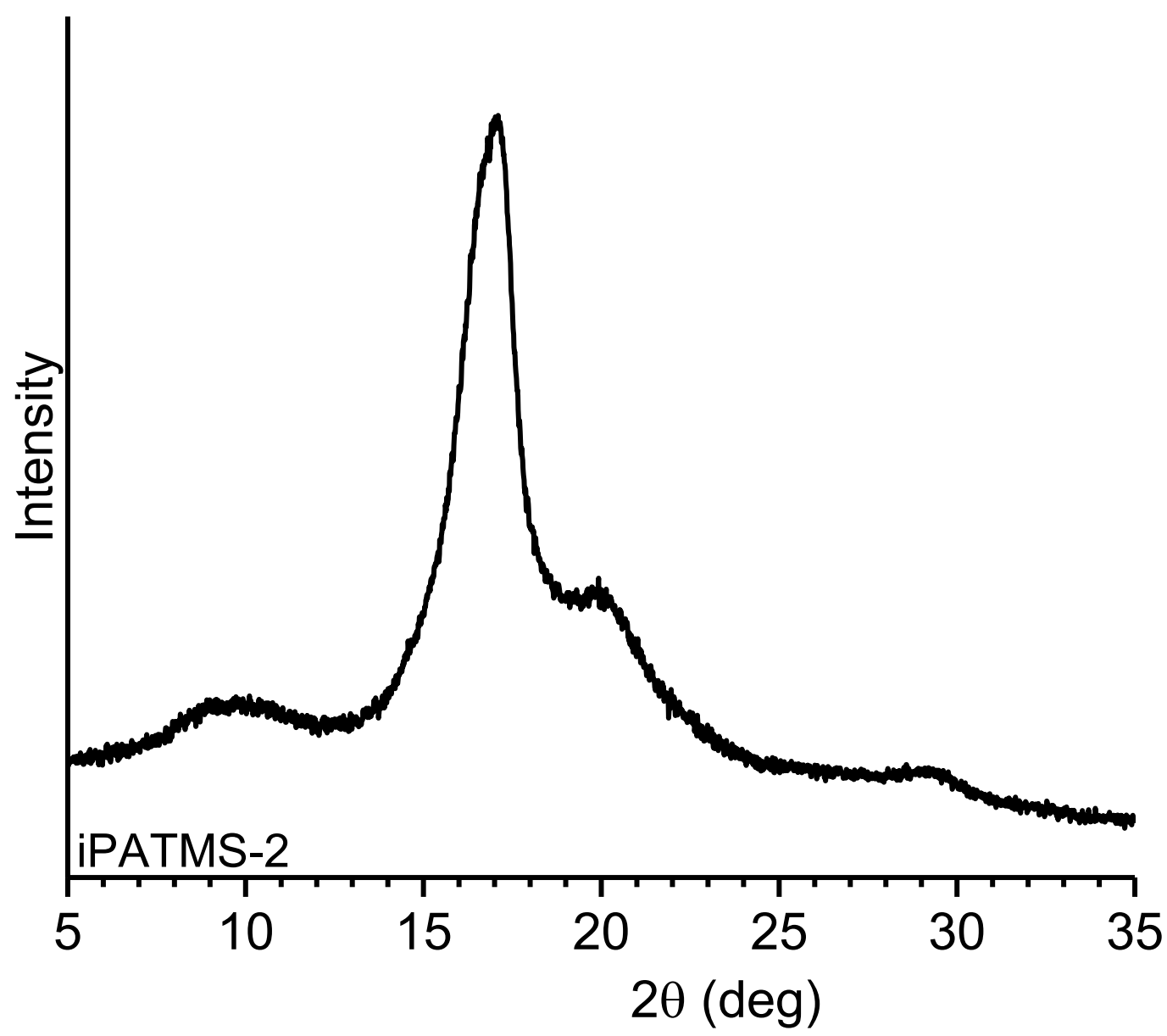

Figure S5. X-ray powder diffraction profile of the as-prepared sample iPATMS-2, synthesized using the pyridylamido-Hf complex **1b** shown in Scheme 1.

2.2) Pyridylamido-Hf Activation Path

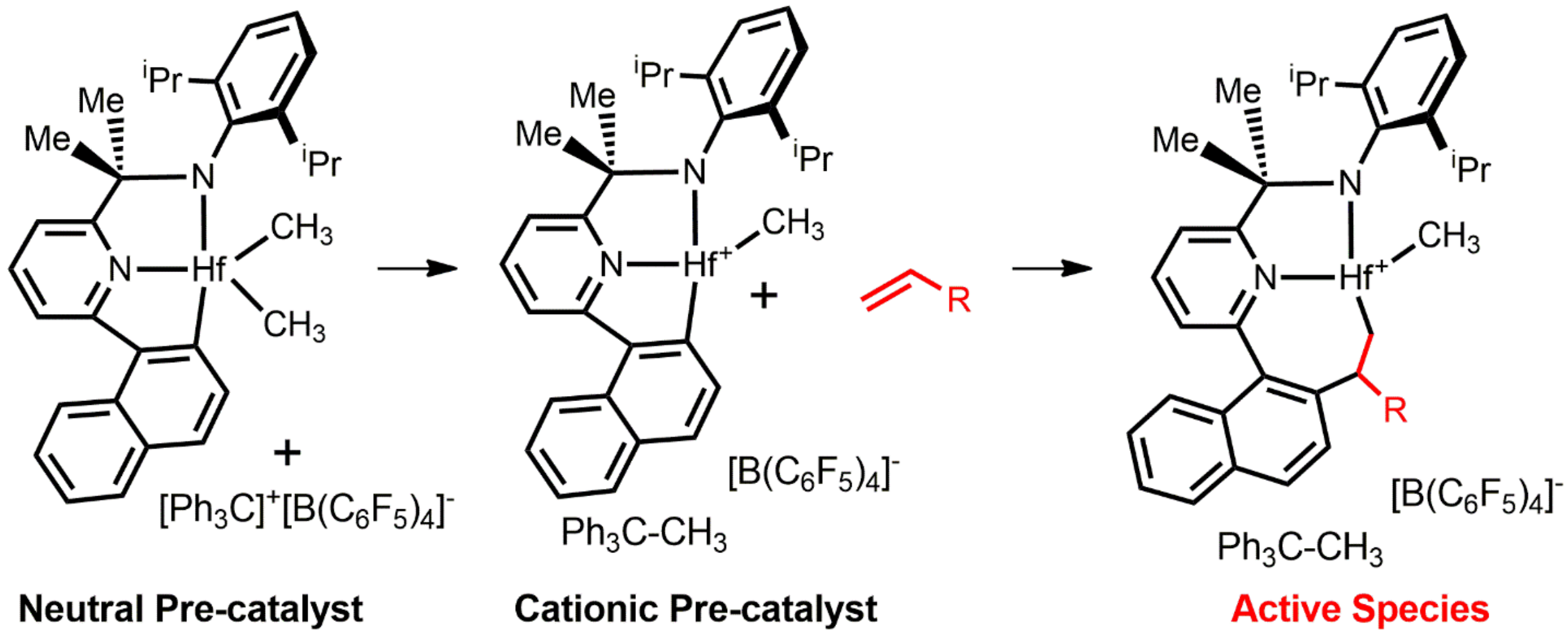

Scheme S3. Schematic representation of pyridylamido-Hf complex activation with ligand modification by first insertion of the α-olefin monomer into the Hf-C_{aryl} bond.

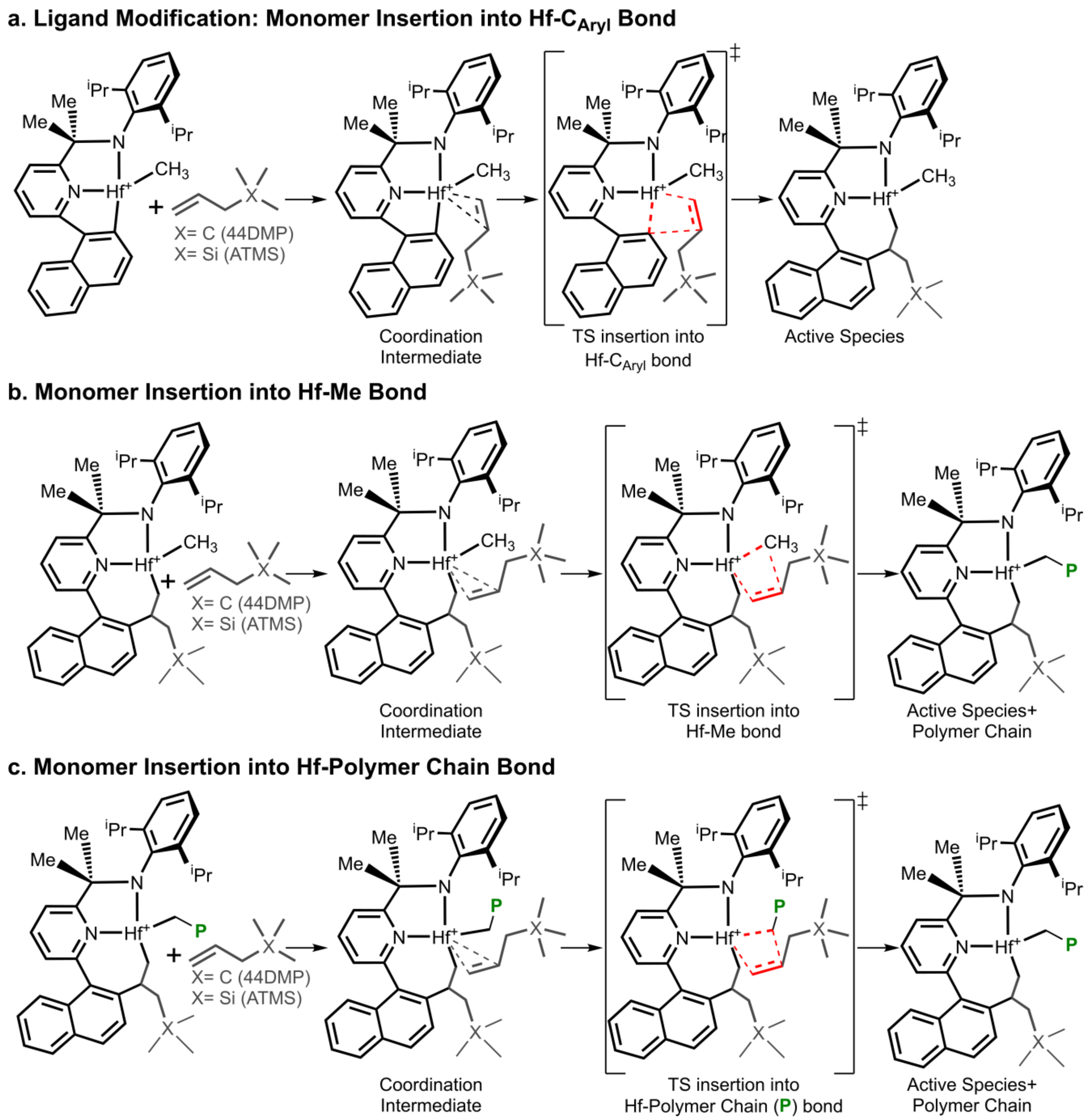

Scheme S4. Schematic representation of steps in 44DMP and ATMS polymerization promoted by pyridylamido-Hf complex: a) Ligand modification, b) Initiation (monomer insertion into Hf-Me bond) and c) Propagation (monomer insertion into Hf-Polymer Chain bond)

2.3) Additional Characterization Data of iP44DMP

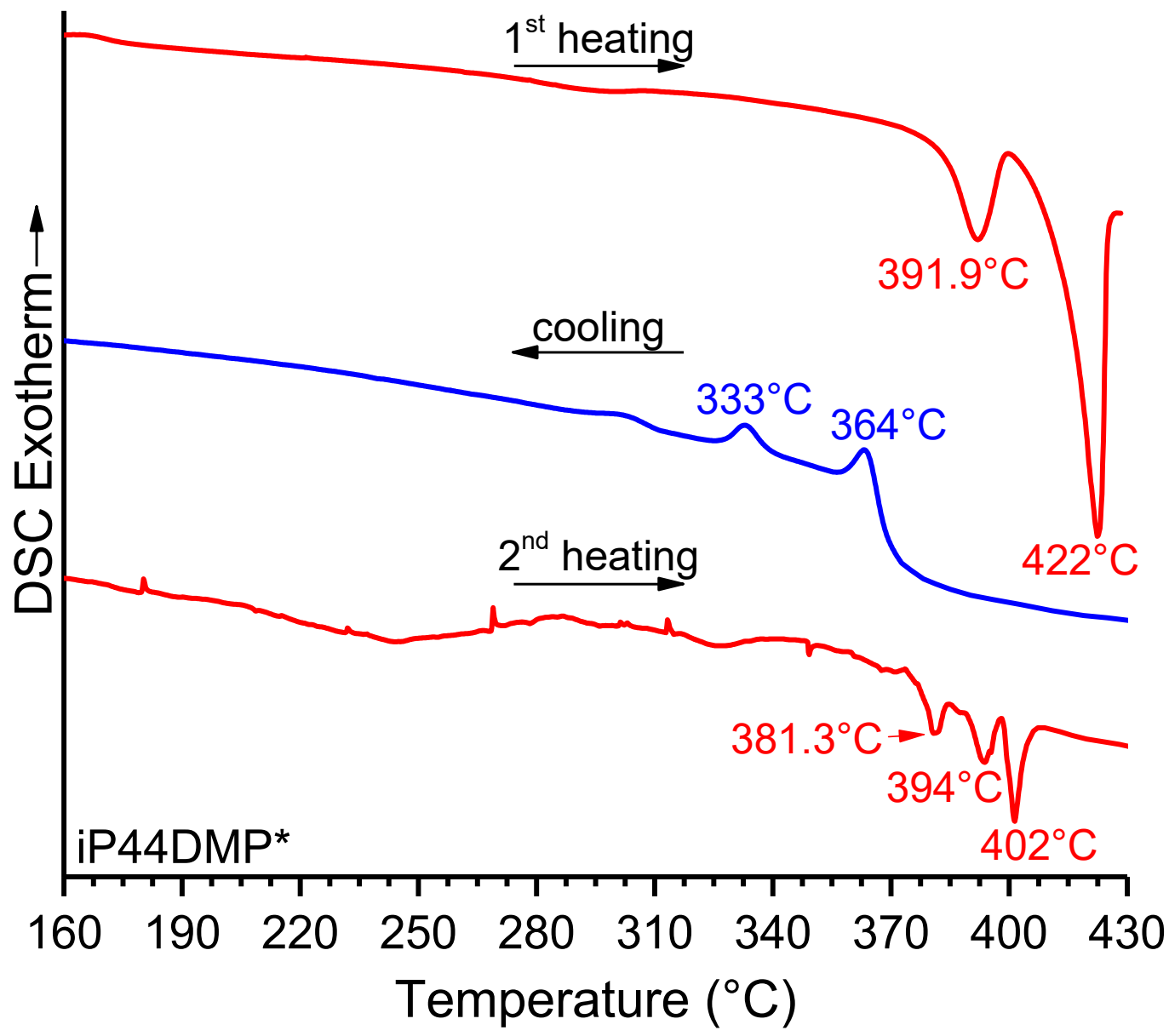

Figure S6. DSC thermograms of the sample iP44DMP* (entry 3 of Table 1) synthesized using the ATMS-pre-activated pyridylamido-Hf complex **1a** in Scheme 1, displaying the first heating, cooling, and successive second heating scans recorded at a scan rate of 10 °C min^{-1}.

Table S4. Melting temperature (T_m^I) and enthalpy (ΔH_m^I) recorded during first heating of as-prepared sample, crystallization temperature (T_c) and enthalpy (ΔH_c) recorded upon cooling from the melt and melting temperature (T_m^{II}) and enthalpy (ΔH_m^{II}) recorded during second heating after melt-crystallization of the sample iP44DMP* extracted from DSC curves of Figure S6.

**Sample**	T_m^I **(°C)**	T_c **(°C)**	T_m^{II} **(°C)**	ΔH_m^I **(J·g^{-1})**	ΔH_c **(J·g^{-1})**	ΔH_m^{II} **(J·g^{-1})**
**iP44DMP***	391.9, 422.0	333, 364	381.3, 394, 402.0	45.7	32.5	20.5

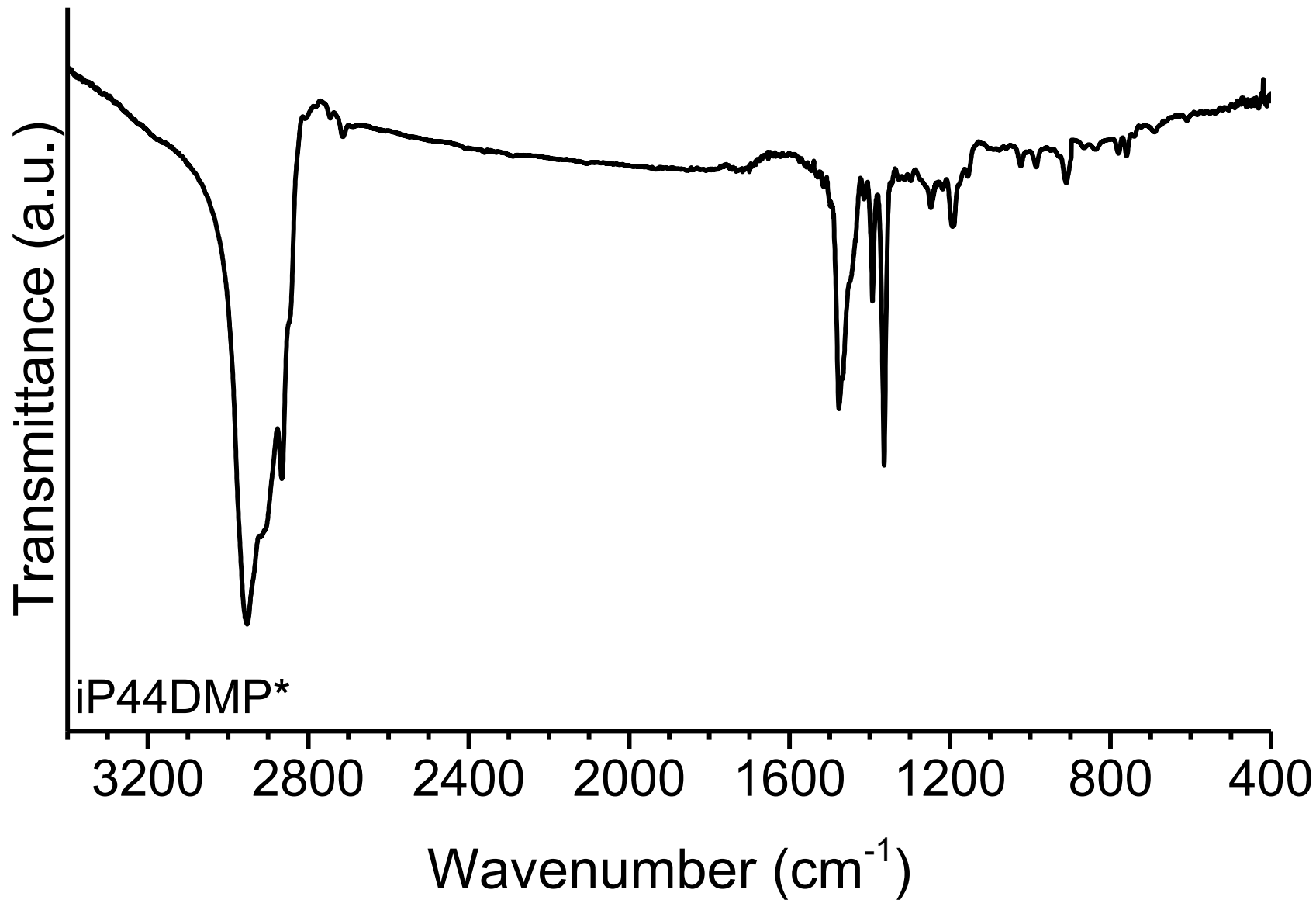

Figure S7. FT-IR spectra of the sample iP44DMP* (entry 3 of Table 1) synthesized using the ATMS-pre-activated pyridylamido-Hf complex **1a** in Scheme 1.

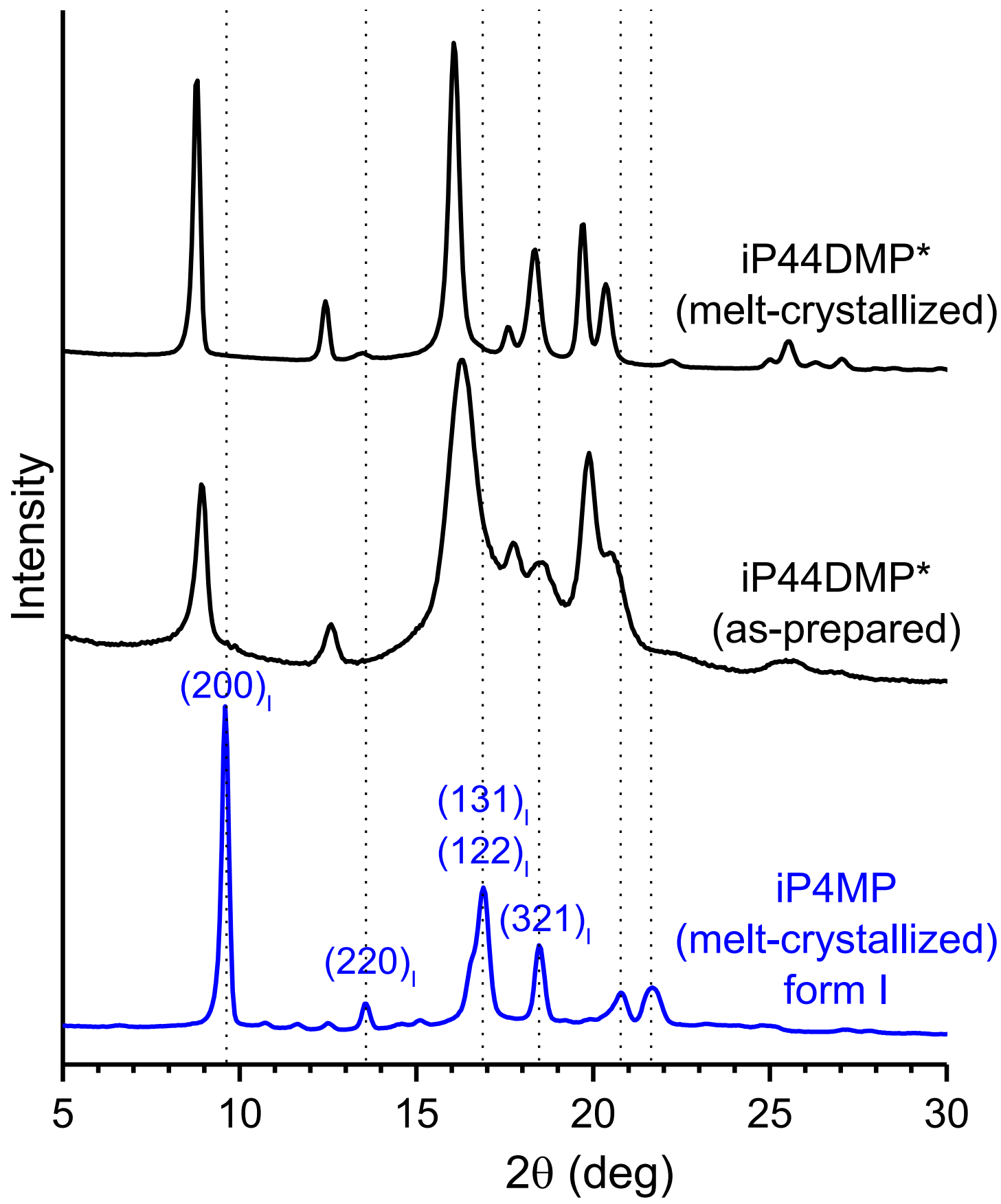

Figure S8. Comparison between the X-ray powder diffraction profiles of the as-prepared and melt-crystallized specimen of the sample iP44DMP* (entry 3 of Table 1) and of a sample of iP4MP crystallized from the melt in the form I. The sample of iP4MP has been synthesized using the same pyridylamido-Hf catalyst **1a** of Scheme 1. The main Bragg reflections characteristic of the form I of iP4MP are indicated.

2.4) Effect of pre-activation variables and monomer concentration on catalytic performances

Table S5. Effect of pre-activation time, ATMS loading, and VCH concentration on VCH polymerization promoted by catalyst **1a**.[a]

Series	Entry	t_{pre} (min)	ATMS/Hf	[VCH] (mol L^{-1})	Yield (mg)	TOF (h^{-1})	T_m (°C)
**Pre-activation time**	1	1	20	0.58	190	83	385
	2	5	20	0.58	480	210	393
	3	15	20	0.58	465	204	392
**ATMS loading**	4	5	2	0.58	320	140	391
	5	5	5	0.58	410	180	393
	6	5	20	0.58	480	210	393
**VCH concentration**	7	5	20	0.30	225	99	378
	8	5	20	0.58	480	210	393
	9	5	20	1.17	610	268	393

[a] Polymerizations were carried out under the same conditions as those reported in Table 1, except for the parameter varied in each series. The reference conditions were t_{pre} = 5 min, ATMS/Hf = 20, and [VCH] = 0.58 mol L^{-1} (entries 2, 6 and 9). T_m values were determined from the first-heating DSC curves reported in Figure S9.

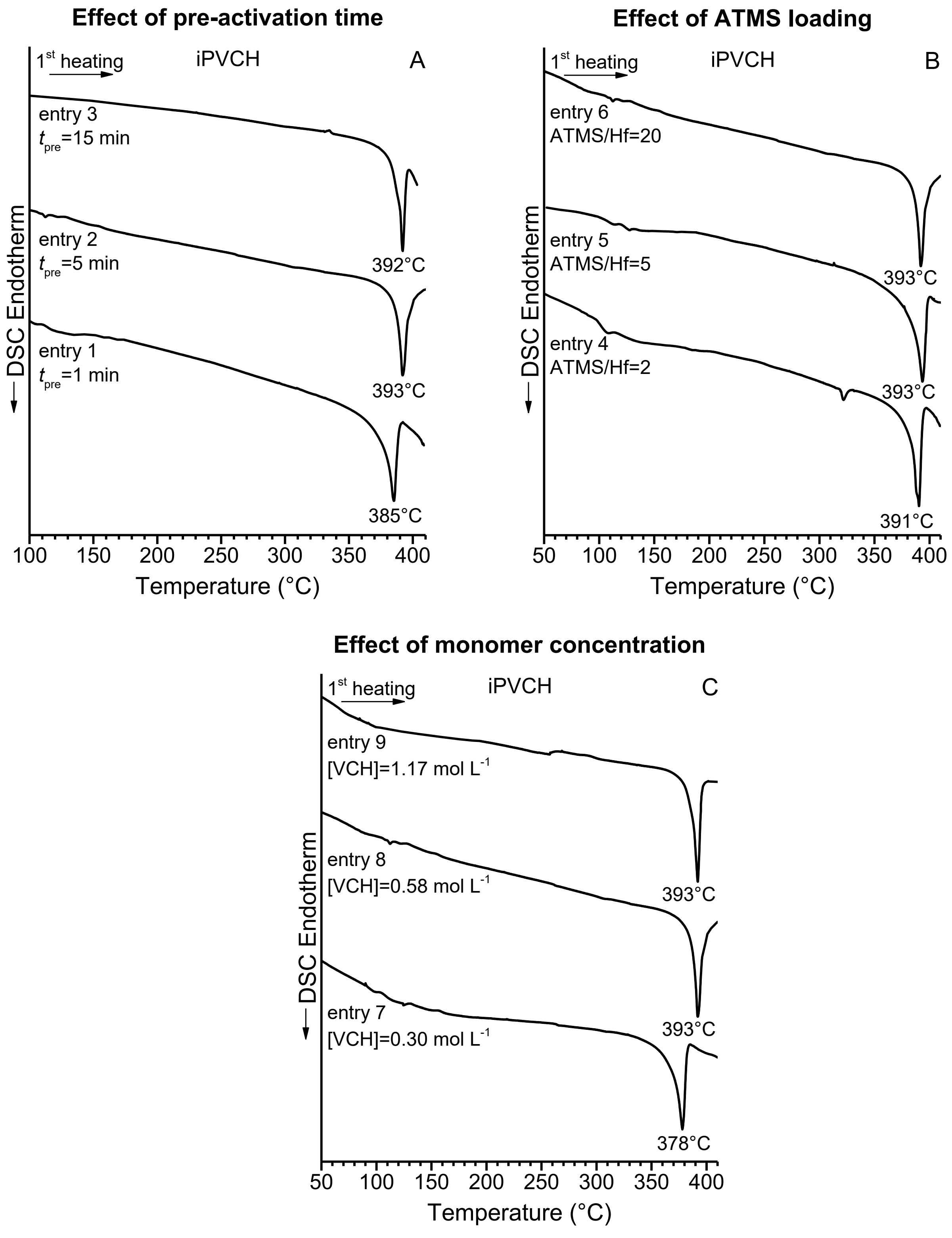

Figure S9. DSC first-heating curves of iPVCH samples synthesized using catalyst **1a** under different ATMS pre-activation conditions: (A) different pre-activation times (1, 5, and 15 min) at constant ATMS loading (20 equiv relative to Hf); (B) different ATMS loadings (2, 5, and 20 equiv relative to Hf) at a fixed pre-activation time of 5 min; and (C) different VCH concentrations (0.30, 0.58, and 1.17 mol L^{-1}) using the standard pre-activation conditions (20 equiv ATMS relative to Hf, 5 min). All other polymerization conditions were kept unchanged.

2.5) Comparative Computational Results for ligand modification by the investigated bulky monomers

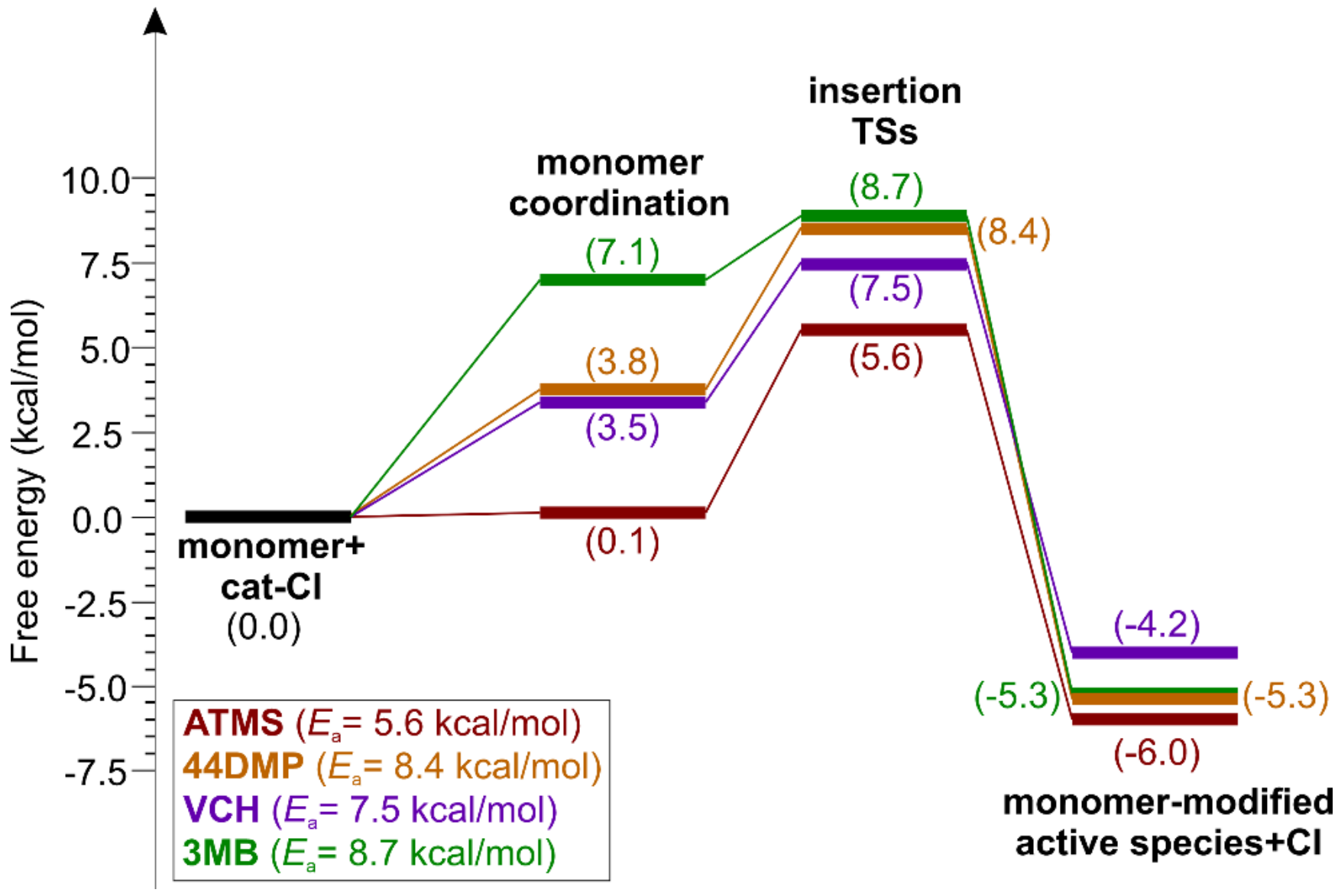

Figure S10. DFT energy profiles for pyridylamido ligand modification by bulky monomers ATMS, 44DMP, VCH and 3MB. The activation energy for ATMS insertion into the Hf-CAryl bond is the lowest, thus suggesting that ligand modification by ATMS is a faster and more favorable reaction with respect to the other investigated monomers.

2.6) Additional Characterization Data of iPVCH and iP3MB

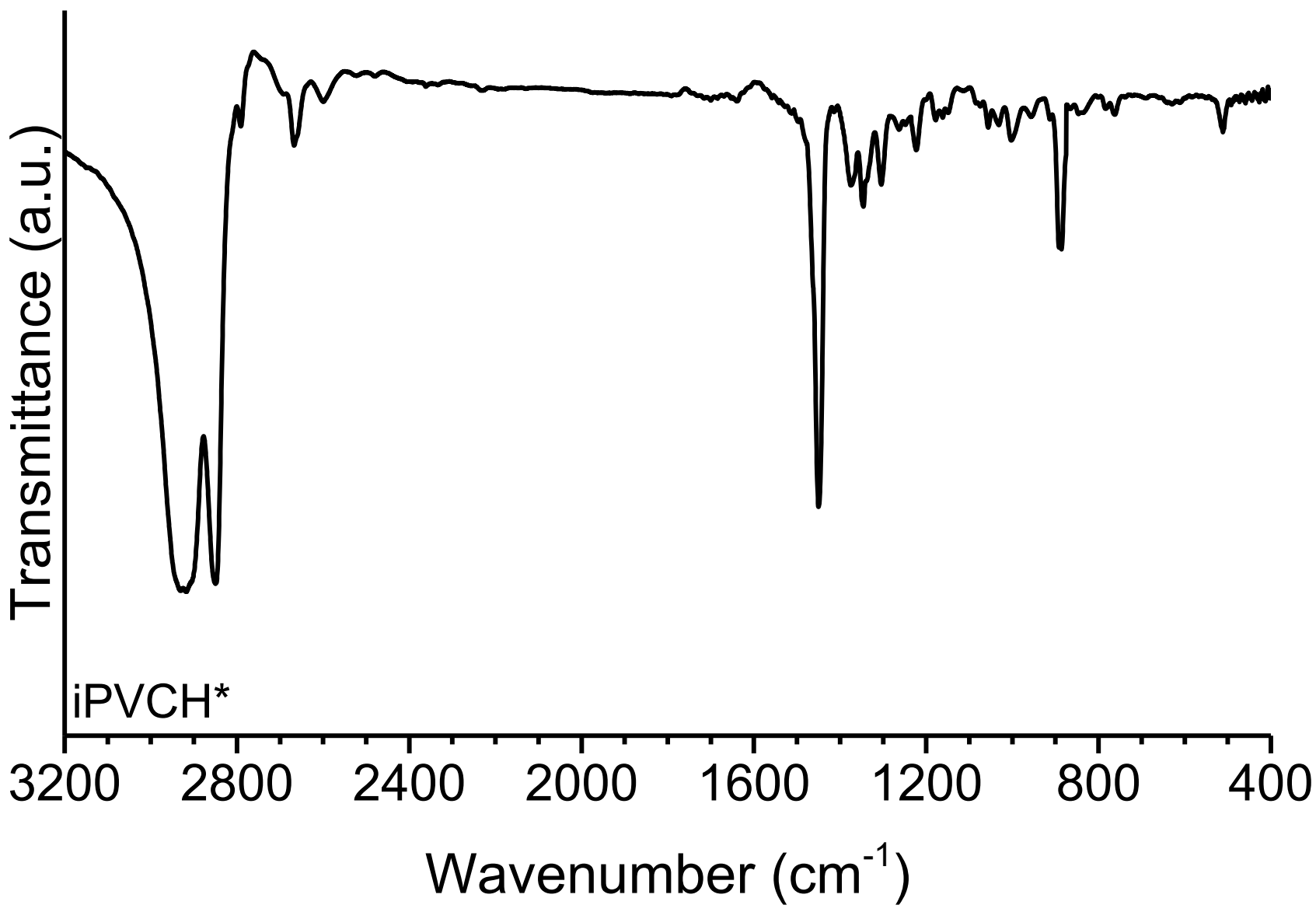

Figure S11. FT-IR spectra of the sample iPVCH* (entry 5 of Table 1) synthesized using the ATMS-pre-activated pyridylamido-Hf complex **1a** in Scheme 1.

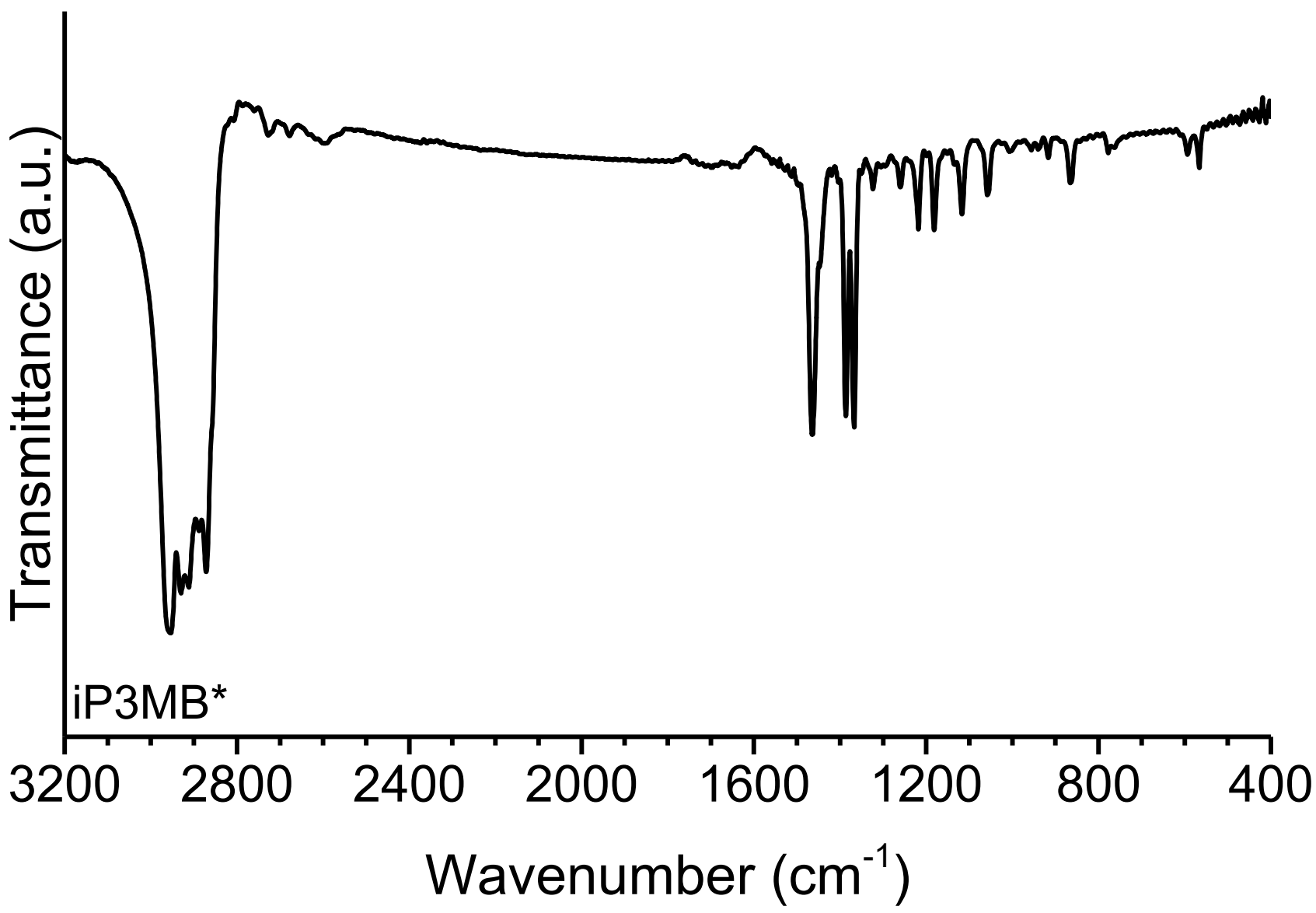

Figure S12. FT-IR spectra of the sample iP3MB* (entry 7 of Table 1) synthesized using the ATMS-pre-activated pyridylamido-Hf complex **1a** in Scheme 1.

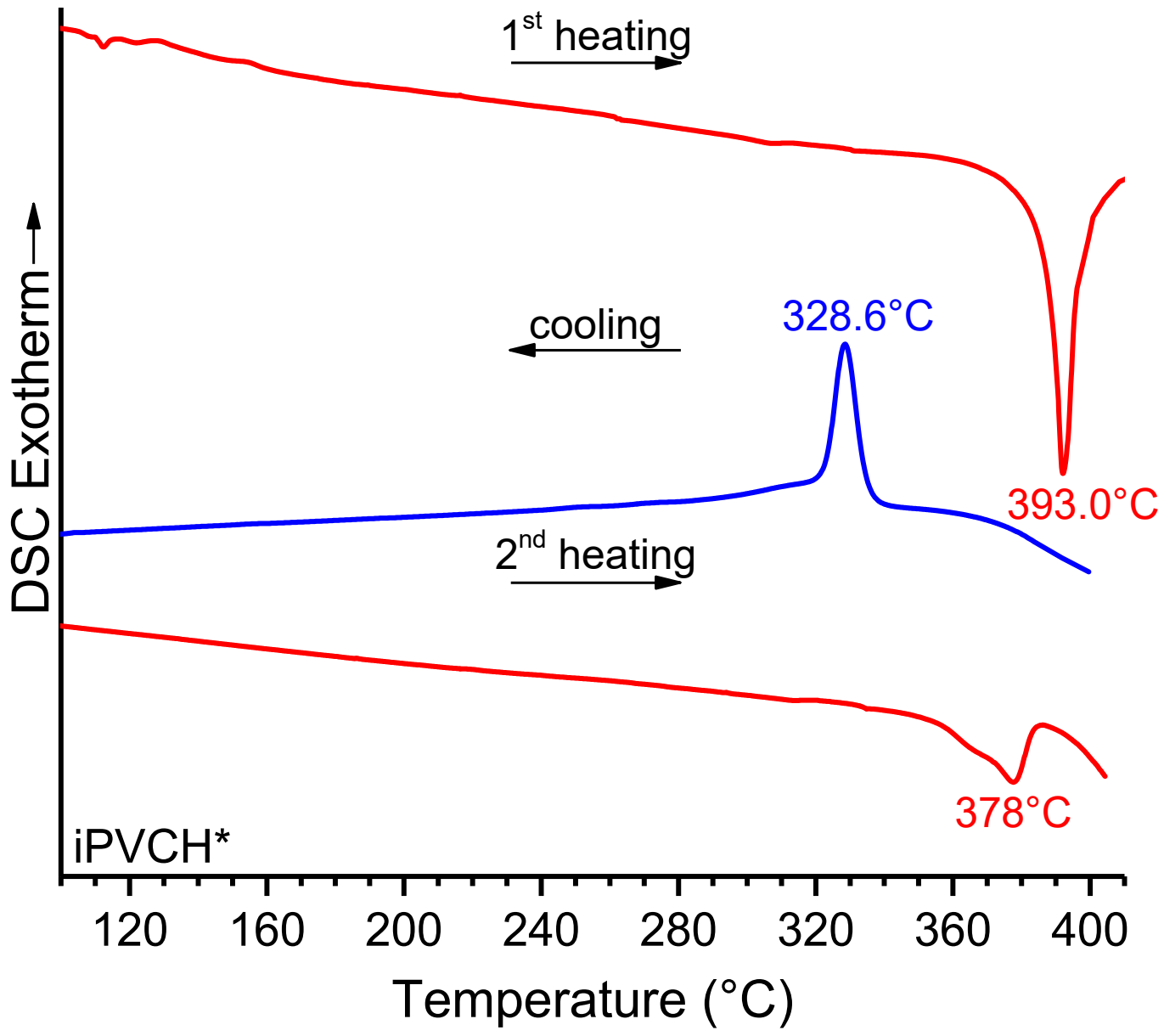

Figure S13. DSC thermograms of the sample iPVCH* (entry 5 of Table 1) synthesized using the ATMS-pre-activated pyridylamido-Hf complex **1a** shown in Scheme 1, displaying the first heating, cooling, and successive second heating scans recorded at a scan rate of 10 °C min^{-1}.

Table S6. Melting temperature (T_m^{I}) and enthalpy (ΔH_m^{I}) recorded during first heating of as-prepared sample, crystallization temperature (T_c) and enthalpy (ΔH_c) recorded upon cooling from the melt and melting temperature (T_m^{II}) and enthalpy (ΔH_m^{II}) recorded during second heating after melt-crystallization of the sample iPVCH* extracted from DSC curves of Figure S13.

**Sample**	**T_m^{I} (°C)**	**T_c (°C)**	**T_m^{II} (°C)**	**ΔH_m^{I} ($J \cdot g^{-1}$)**	**ΔH_c ($J \cdot g^{-1}$)**	**ΔH_m^{II} ($J \cdot g^{-1}$)**
**iPVCH***	393.0	328.6	378.0	16.1	24.2	12.5

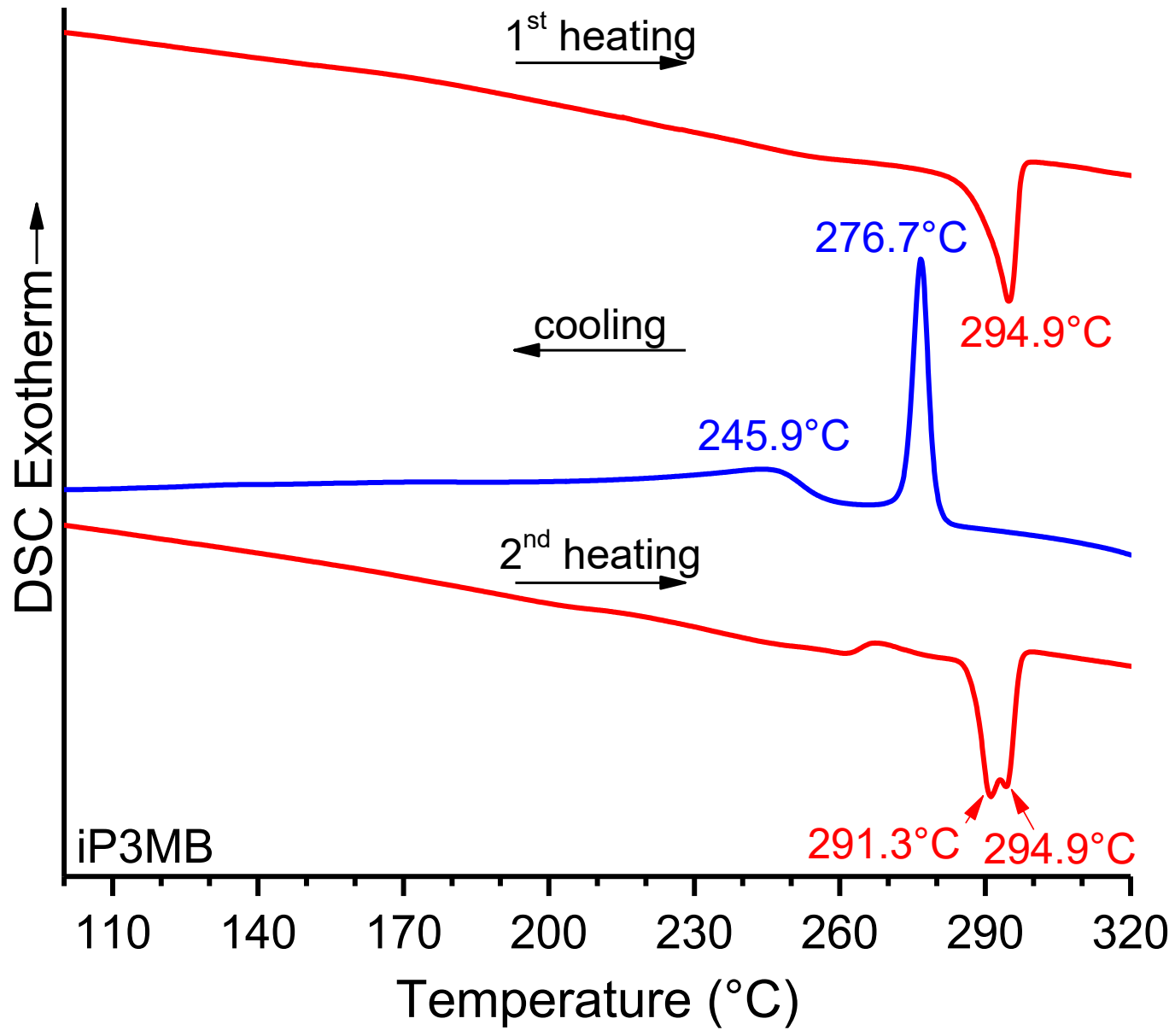

Figure S14. DSC thermograms of the sample iP3MB (entry 6 of Table 1) synthesized using the pyridylamido-Hf complex **1a** shown in Scheme 1, displaying the first heating, cooling, and successive second heating scans recorded at a scan rate of 10 °C min^{-1}.

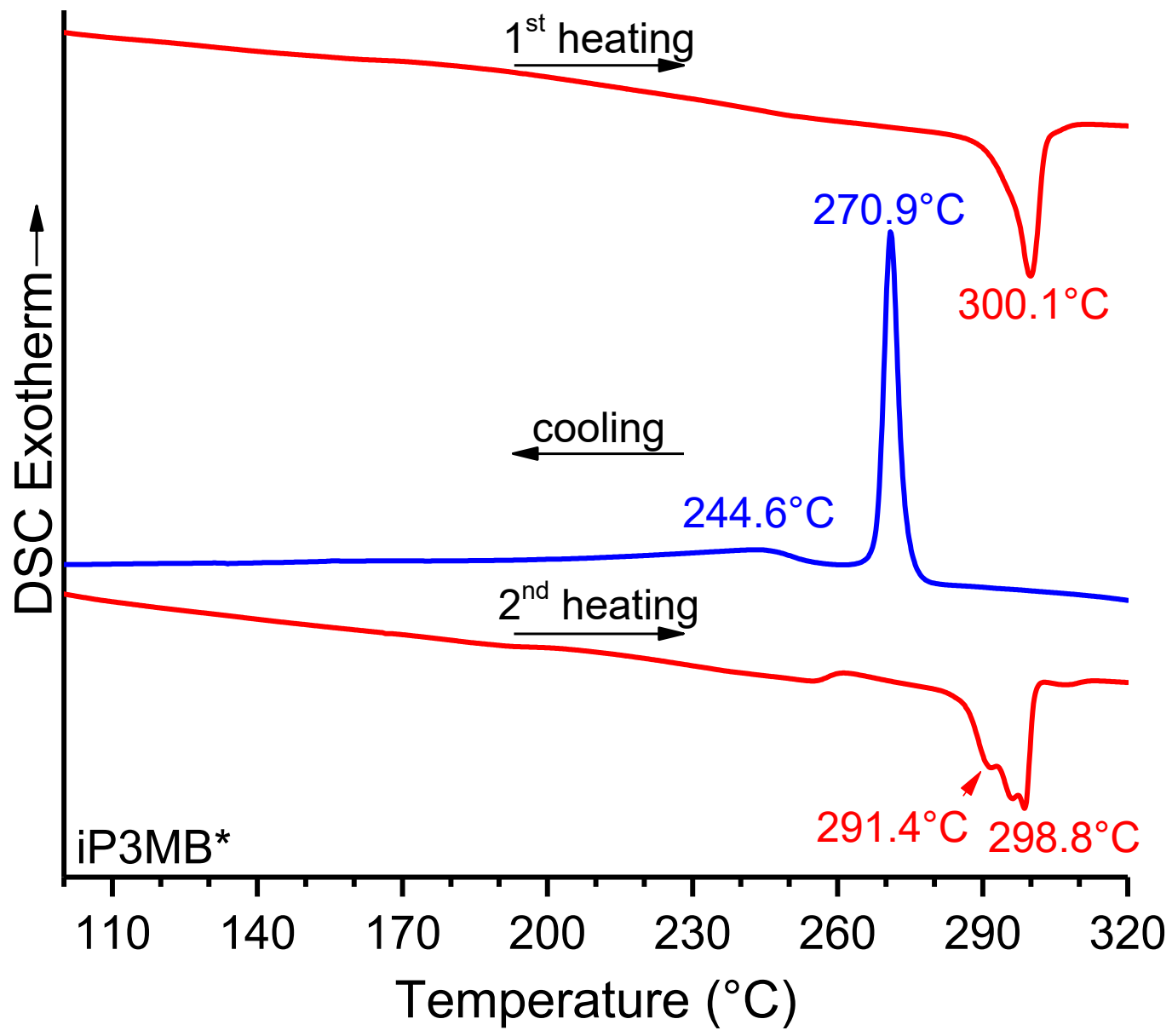

Figure S15. DSC thermograms of the sample iP3MB* (entry 7 of Table 1) synthesized using the ATMS-pre-activated pyridylamido-Hf complex **1a** shown in Scheme 1, displaying the first heating, cooling, and successive second heating scans recorded at a scan rate of 10 °C min^{-1}.

Table S7. Melting temperature (T_m^I) and enthalpy (ΔH_m^I) recorded during first heating of as-prepared samples, crystallization temperature (T_c) and enthalpy (ΔH_c) recorded upon cooling from the melt and melting temperature (T_m^{II}) and enthalpy (ΔH_m^{II}) recorded during second heating after melt-crystallization of samples iP3MB and iP3MB* extracted from DSC curves of Figures S14 and S15.

Sample	T_m^I (°C)	T_c (°C)	T_m^{II} (°C)	ΔH_m^I ($J \cdot g^{-1}$)	ΔH_c ($J \cdot g^{-1}$)	ΔH_m^{II} ($J \cdot g^{-1}$)
**iP3MB**	294.9	245.9, 276.7	291.3, 295.9	37.2	56.7	40.5
**iP3MB***	300.1	244.6, 270.9	291.4, 298.8	49.6	54.6	49.0

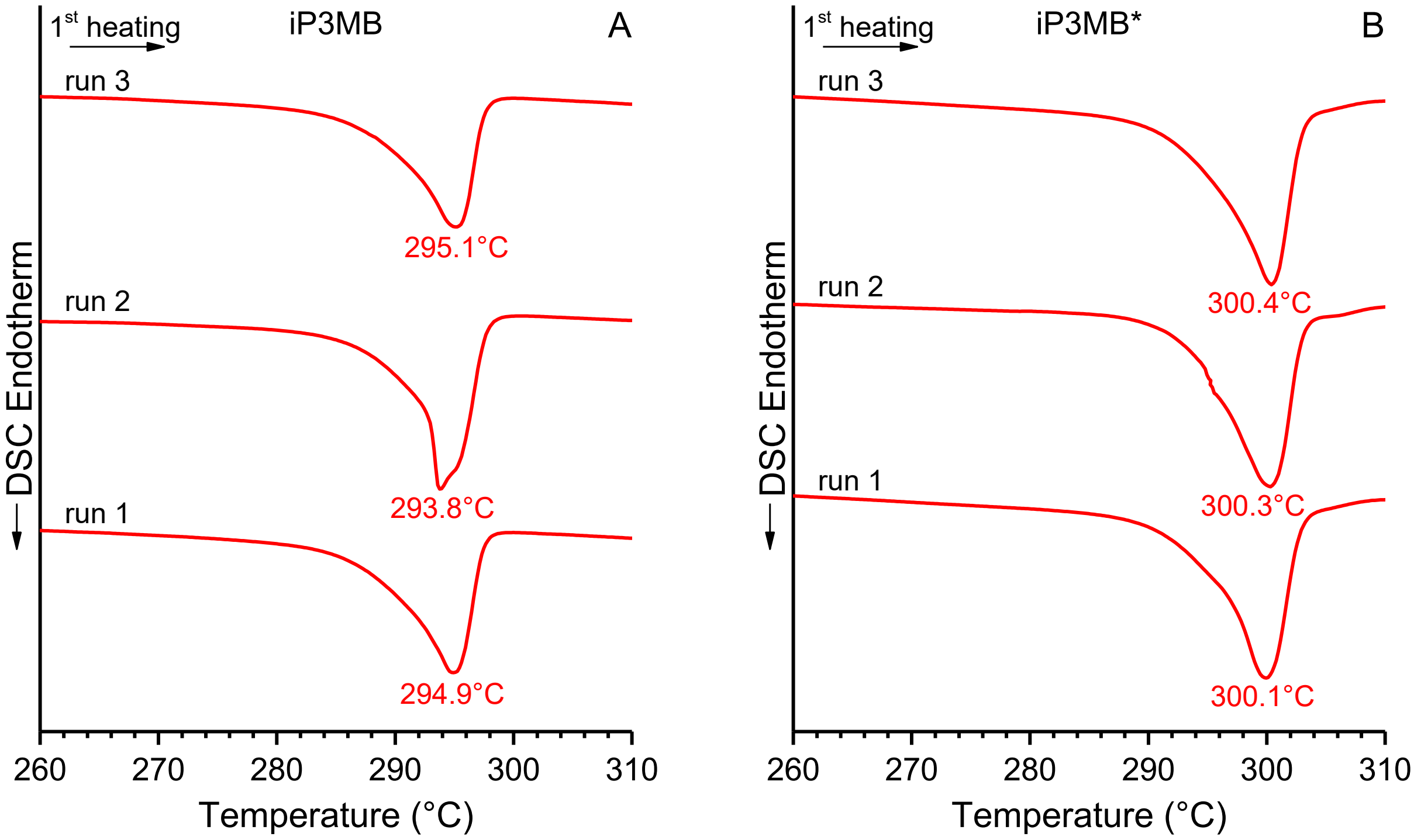

Figure S16. Second-heating DSC curves of three independent iP3MB samples obtained from separate polymerization runs using (A) the conventional activation procedure and (B) the ATMS pre-activation strategy.

Table S8. Melting temperatures determined for iP3MB samples obtained with catalyst **1a** from three independent polymerization experiments under conventional conditions and following ATMS pre-activation.

Sample	Catalyst activation protocol	$T_{m,1}$ (°C)[a]	$T_{m,2}$ (°C)[a]	$T_{m,3}$ (°C)[a]	T_m ± SD (°C)
**iP3MB**	Conventional activation	294.9	293.8	295.1	294.6 ± 0.7
**iP3MB***	ATMS pre-activation	300.1	300.3	300.4	300.3 ± 0.2

[a] T_m values were determined from the first-heating DSC curves reported in Figures S16A and S16B.

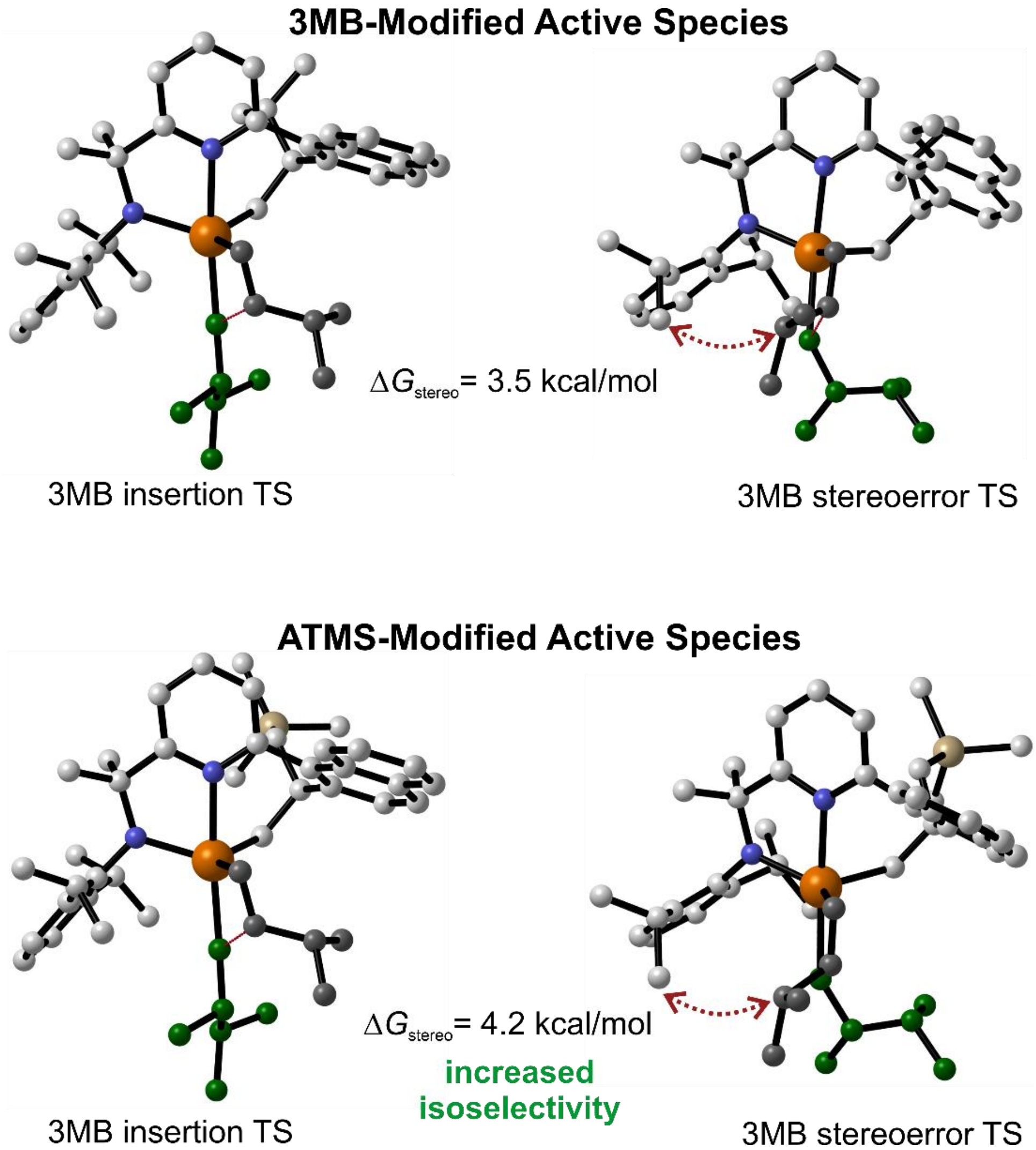

Figure S17. DFT optimized geometries for 3MB insertion and stereoerror TSs in the presence of 3MB-modified (top) and ATMS-modified active species (bottom). The computed free energies for stereoselectivity (ΔG_{stereo}) are reported.

2.7) Thermogravimetric Analysis of Bulky-Monomer-Derived Polyolefins

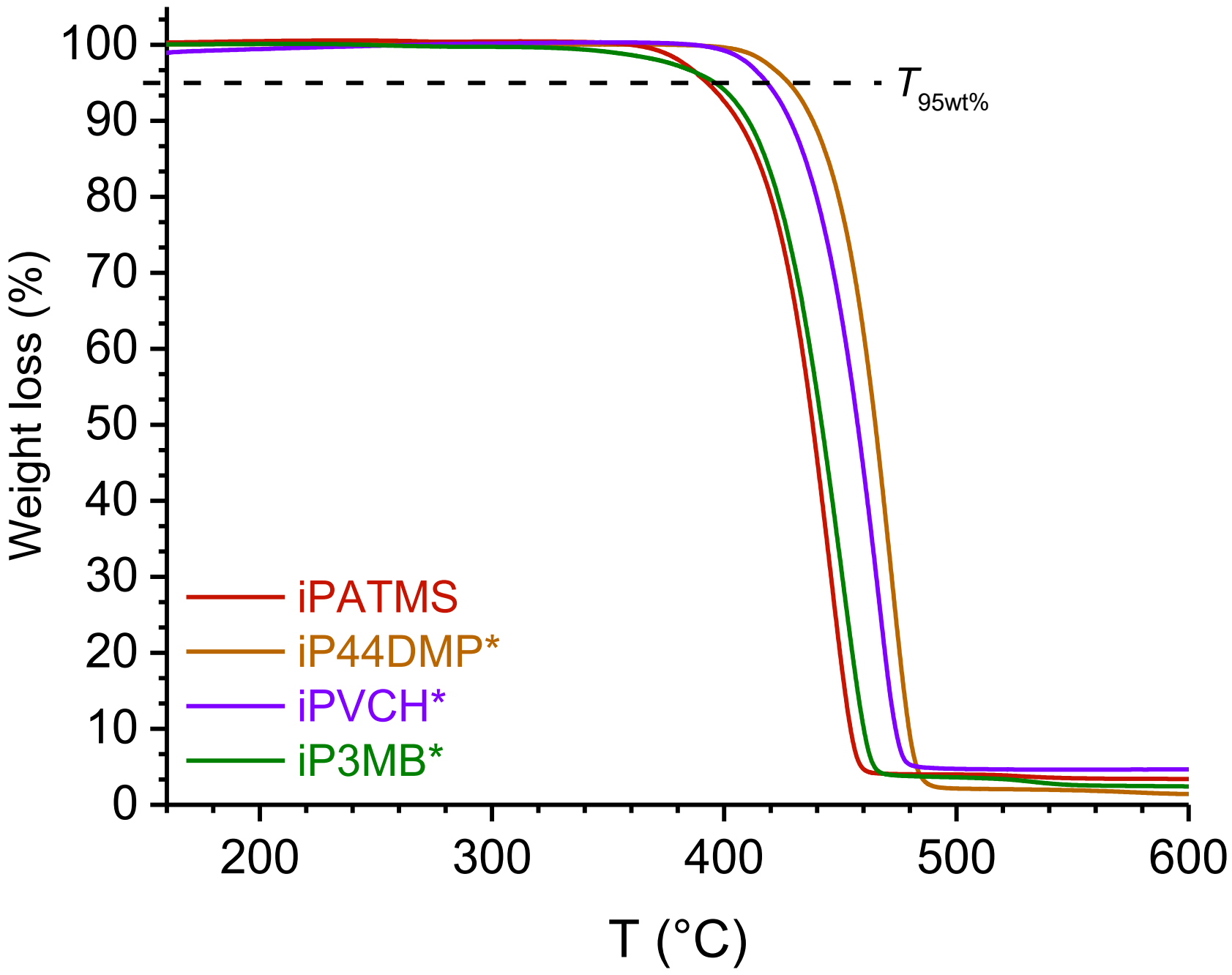

Figure S18. TGA curves of iPATMS, iP44DMP*, iPVCH*, and iP3MB* recorded under flowing N_2 at a heating rate of 10 °C min^{-1}.

Table S9. Thermal degradation parameters of the bulky-monomer-derived polyolefins investigated in this work, as determined by TGA under flowing N_2 at a heating rate of 10 °C min^{-1}. $T_{95\%}$ denotes the temperature corresponding to 5% mass loss, while T_d is the temperature at the maximum rate of mass loss.

Sample	T_m (°C)[a]	$T_{95\%}$ (°C)[b]	T_d (°C)[c]	$T_{95\%}$ - T_m (°C)
**iPATMS**	308	≈400	≈435	≈92
**iP44DMP***	422	≈430	≈460	≈8
**iPVCH***	393	≈420	≈450	≈27
**iP3MB***	300	≈400	≈435	≈100

[a] Melting temperature determined from the DSC heating scan reported in Figure 1.
[b]Temperature at which 95% of the initial mass is retained, corresponding to 5% mass loss.
[c]Degradation temperature, defined as the temperature at the maximum rate of mass loss.

2.8) Effect of Increased TIBA Loading on Polymerization Performance and Thermal Properties

Table S10. Effect of increased Al(^{i}Bu)$_3$ loading on the polymerization performance and thermal properties of iPATMS, iP44DMP, iPVCH and iP3MB.[a]

Sample[b]	Al(^{i}Bu)$_3$ loading (mmol)	Yield (mg)	TOF (h^{-1})	T_m^I (°C)	T_c (°C)	T_m^I-T_c (°C)
iPATMS	0.50	450	3425	308.0	275.9	32.1
**iPATMS-HAl**	**1.50**	**360**	**2740**	**305.7**	**290.0**	**15.7**
iP44DMP*	0.50	230	170	422.0	364.0	58.0
**iP44DMP*-HAl**	**1.50**	**195**	**144**	**394.6**	**381.5**	**13.1**
iPVCH*	0.50	480	210	393.0	328.6	64.4
**iPVCH*-HAl**	**1.50**	**300**	**132**	**377.1**	**347.4**	**29.7**
iP3MB*	0.50	310	213	300.1	270.9	29.2
**iP3MB*-HAl**	**1.50**	**270**	**186**	**291.0**	**276.7**	**14.3**

[a] Except for the higher Al(^{i}Bu)$_3$ loading, all polymerizations were carried out under the same conditions as the corresponding experiments performed at the conventional scavenger concentration reported in Table 1, whose data are also reproduced here for comparison. [b] HAl denotes samples synthesized using an increased amount of Al(^{i}Bu)$_3$ scavenger.

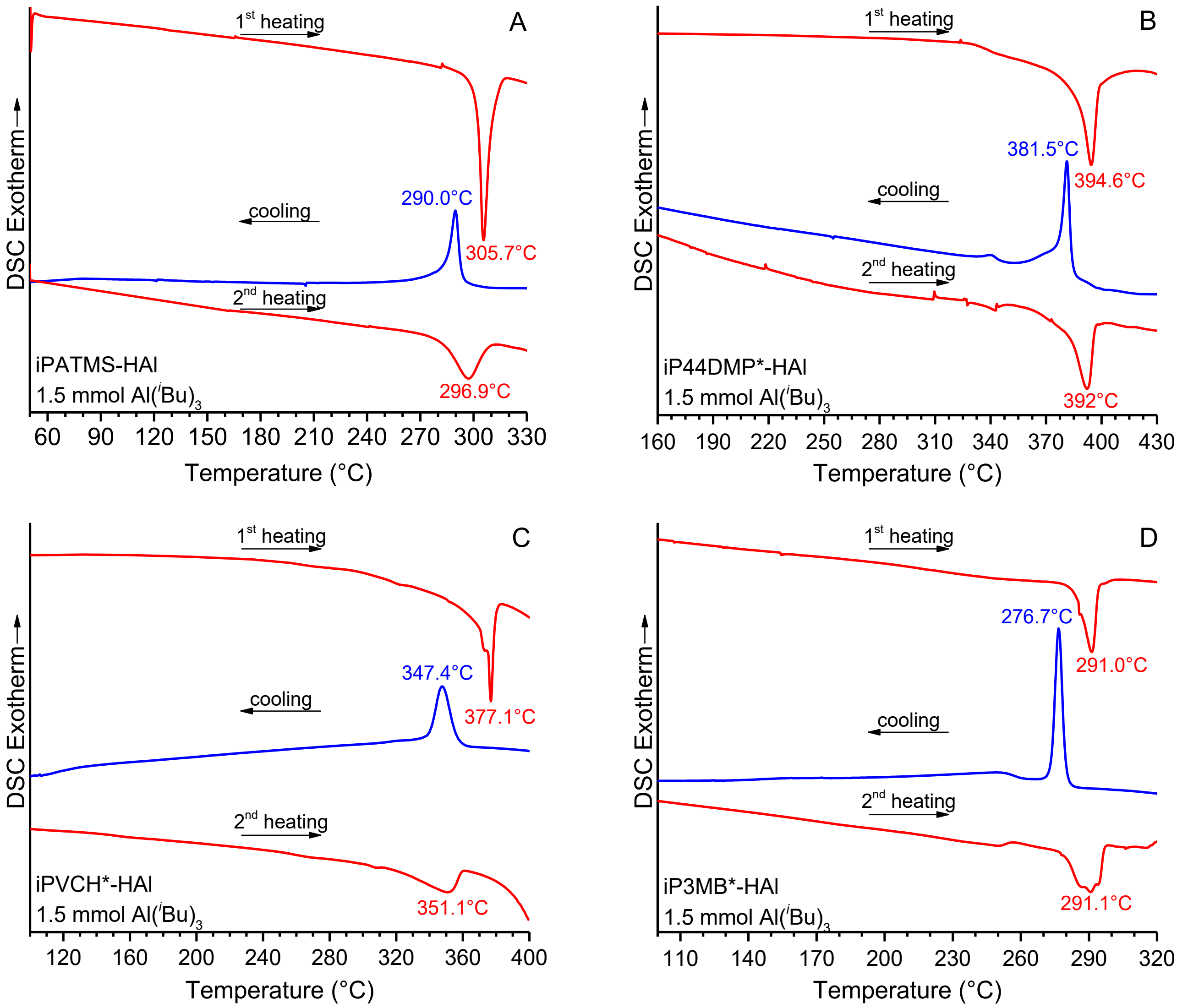

Figure S19. DSC thermograms, including the first-heating, cooling, and second-heating scans, of (A) iPATMS-HAl, (B) iP44DMP*-HAl, (C) iPVCH*-HAl, and (D) iP3MB*-HAl samples synthesized using an increased Al(^{i}Bu)$_3$ loading.

2.9) Additional Data of Bulky Monomers Polymerization Promoted by Catalyst 1b

Table S11. Results of polymerizations of 44DMP, VCH and 3MB (Scheme 1) catalyzed by **1b** of Scheme 1 and by **1b** with ATMS-modified active species. Monomer concentration, polymerization time (t_{pol}), polymer yield, average turnover frequency (TOF), melting temperatures (T_m) of the resulting isotactic polymers.[a]

Entry	Monomer	Polymer[b]	[Monomer] (mol·L^{-1})	t_{pol} (min)	Yield (mg)	TOF (h^{-1})[c]	T_m (°C)[d]
**1**	44DMP	iP44DMP-1b	0.39	60	-	-	-
**2**	44DMP	iP44DMP*-1b	0.39	60	415	306	423
**3**	VCH	iPVCH-1b	0.58	90	-	-	-
**4**	VCH	iPVCH*-1b	0.58	90	1550	680	394
**5**	3MB	iP3MB-1b	0.71	90	230	158	298
**6**	3MB	iP3MB*-1b	0.71	90	620	427	302

[a] 13.8 μmol precatalyst, 18.5 μmol co-catalyst $[Ph_3C][B(C_6F_5)_4]$, 0.50 mmol $Al(^iBu)_3$, 800 rpm, 60 min of solvent (toulene)/monomer/scavenger contact, [B]/[Hf]=1.3, T_{pol}=25 °C. [b] Samples marked with * have been obtained with ATMS-modified active species of the pyridylamido complex (2 in Scheme 2). ATMS (45μL, 20 eq with respect to the catalyst) was precontacted with the catalyst solution for 5 minutes prior to injection (prepolymerization step). [c] Average turnover frequency (TOF): mol monomer/mol Hf ×·h. [d] Highest melting temperature values of the obtained polymers in the two polymerization conditions (1 and 2 in Scheme 2).

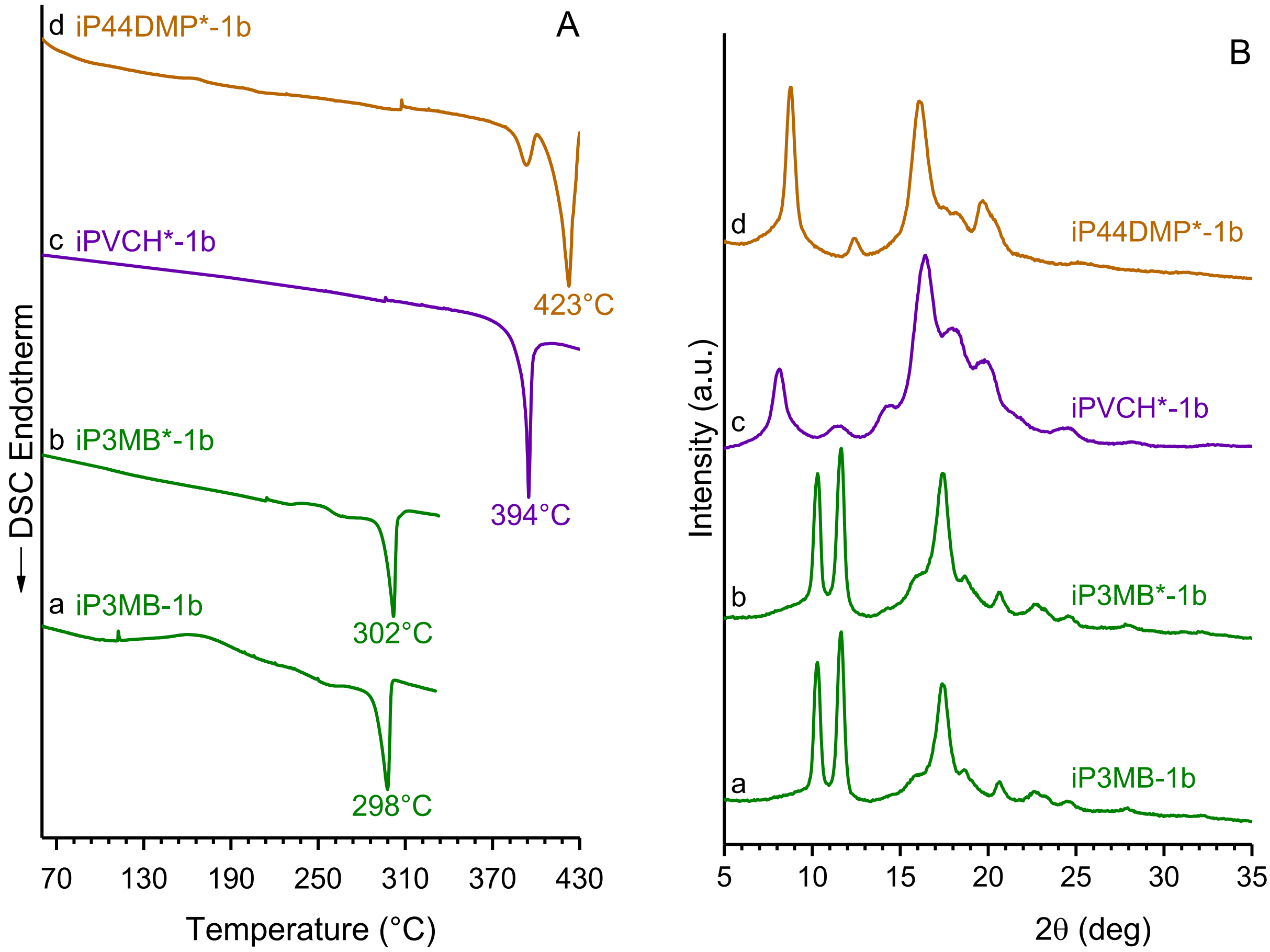

Figure S20. DSC heating curves (A) and X-ray diffraction profiles (B) of iP3MB-1b (a), synthesized with catalyst **1b** of Scheme 1 under conventional condition (path a in Figure 2), and iP3MB*-1b (b), iPVCH*-1b (c) and iP44DMP*-1b (d), synthesized *via* ATMS-induced pre-activation of catalyst **1b** (path b in Figure 2).

2.10) Additional Data of Propene and 4-Methyl-1-Pentene Polymerization and Computational Data on 3MB and 4MP Propagation

For the homopolymerization of 4-methyl-1-pentene and propene under ATMS pre-activation conditions, additional control experiments were performed using a lower ATMS loading (5 eq. with respect to the precatalyst), in comparison to the standard 20 eq. employed in this study. These experiments were designed to exclude the possibility that ATMS might act as a comonomer rather than solely as a pre-activating agent.

The polymers obtained using 5 and 20 equivalents of ATMS exhibited essentially identical thermal properties, including comparable melting temperatures and melting enthalpies. These results indicate that ATMS does not significantly participate in chain propagation under the polymerization conditions employed but rather functions primarily as a pre-activation agent for the generation of the active catalytic species.

However, minor signals of very low intensity attributable to ATMS-derived units were detected in the NMR spectra. These are plausibly associated with short ATMS-ATMS sequences formed during the pre-activation stage immediately following catalyst activation. Given their negligible intensity and the indistinguishable thermal properties of the resulting polymers, incorporation of ATMS into the final polymer backbone can be considered negligible.

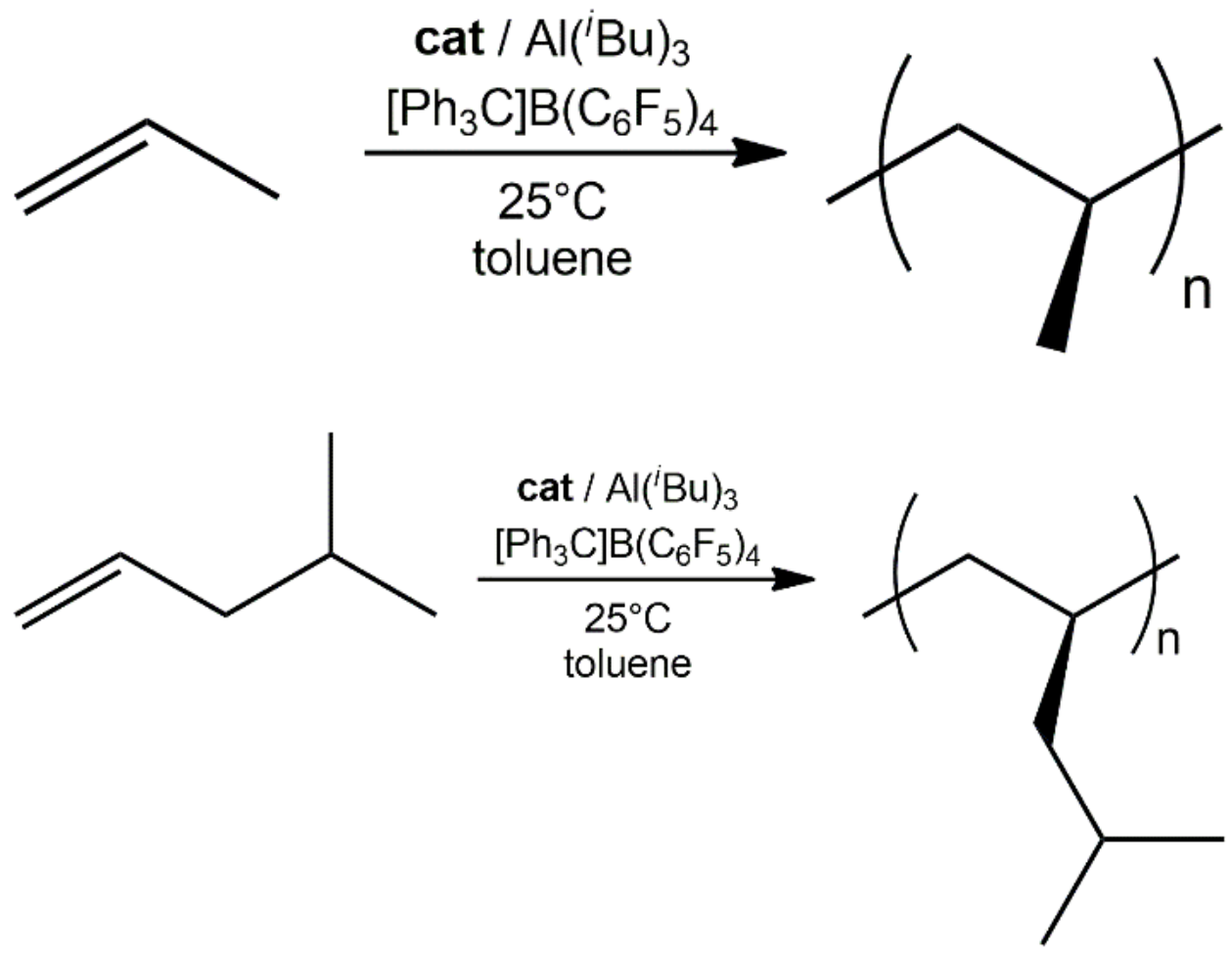

Scheme S5. Homopolymerization of propene and 4-methyl-1-pentene catalyzed by the pyridyl-amido hafnium complex **1a** of Scheme 1, carried out both in the absence of ATMS pre-activation and in the presence of ATMS using two different ATMS/Hf ratios (20:1 and 5:1). Polymerization data are reported in Tables S12 and S13.

Table S12. Results of propene polymerization catalyzed by the pyridylamido hafnium complex **1a** in Scheme 1, performed both in the absence of pre-activation and under ATMS-induced pre-activation at different ATMS/Hf ratios.[a]

Entry	Sample[b]	[ATMS]/[Hf]	P_{C3} (bar)	t_{pol} (min)	Yield (mg)	TOF (h^{-1})[c]	M_n (M_w/M_n) (kg mol^{-1})[f]
**1**	iPP	-	1.0	30	983	3386	150 (2.0)
**2**	iPP*	20:1	1.0	30	1560	5376	145 (2.0)
**3**	iPP**	5:1	1.0	30	1480	5097	155 (2.1)

[a] 13.8 µmol precat., 18.5 µmol co-cat., 0.50 mmol Al(^{i}Bu)$_3$, 800 rpm, 60 min of solvent (toulene)/monomer/scavenger precontact, [B]/[Hf]=1.3, T_{pol}=25 °C.
[b] Samples marked with * and ** have been obtained with ATMS-modified active species of pyridylamido complex **1a**. ATMS (45µL, 20 eq with respect to the catalyst for the sample iPP* and 12µL, 5 eq with respect to the catalyst for the sample iPP**) was precontacted with the catalyst solution for 5 minutes prior to injection (prepolymerization step).
[c] Average turnover frequency (TOF): mol monomer/mol Hf·h.
[f] Determined by GPC.

Table S13. Results of 4-methyl-1-pentene (4MP) homopolymerization catalyzed by the pyridylamido hafnium complex **1a** shown in Scheme 1, performed both in the absence of pre-activation and under ATMS-induced pre-activation at different ATMS/Hf ratios.[a]

Entry	Sample[b]	[ATMS]/[Hf]	[M] (mol·L^{-1})	t_{pol} (min)	Yield (mg)	TOF (h^{-1})[c]	M_n (M_w/M_n) (kg mol^{-1})[f]
**1**	iP4MP	-	0.45	30	690	1188	205 (2.3)
**2**	iP4MP*	20:1	0.45	30	790	1360	222 (2.0)
**3**	iP4MP**	5:1	0.45	30	750	1291	195 (2.1)

[a] 13.8 µmol precat., 18.5 µmol co-cat., 0.50 mmol Al(^{i}Bu)$_3$, 800 rpm, 60 min of solvent (toulene)/monomer/scavenger precontact, [B]/[Hf]=1.3, T_{pol}=25 °C.
[b] Samples marked with * and ** have been obtained with ATMS-modified active species of pyridylamido complex. ATMS (45µL, 20 eq with respect to the catalyst for the sample iP4MP* and 12µL, 5 eq with respect to the catalyst for the sample iP4MP**) was precontacted with the catalyst solution for 5 minutes prior to injection (prepolymerization step).
[c] Average turnover frequency (TOF): mol monomer/mol Hf·h.
[f] Determined by GPC.

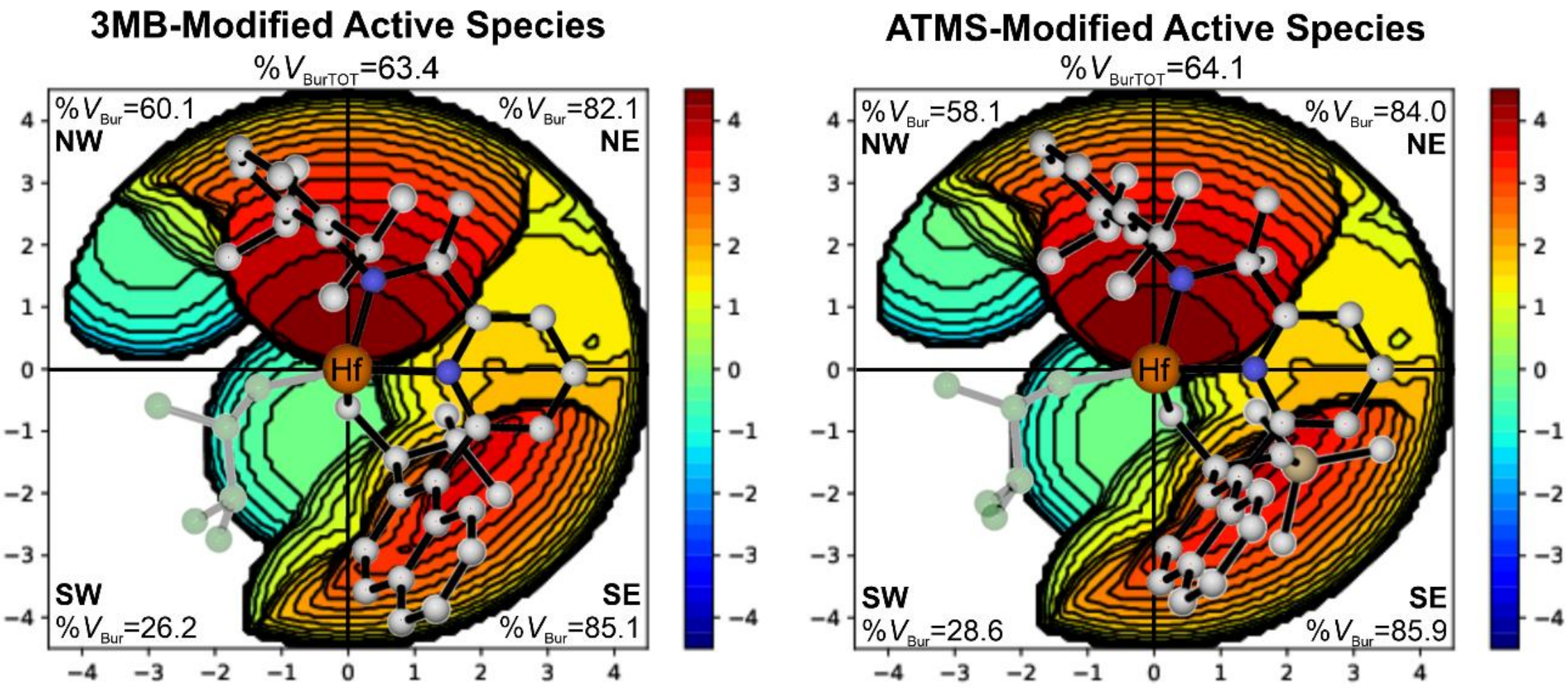

Figure S21. Steric maps of buried volume (%V_{Bur}) computed on the 3MB- and ATMS-modified propagating active species. The buried volume was computed using a sphere of 4.5 Å radius centered on the Hf atom.

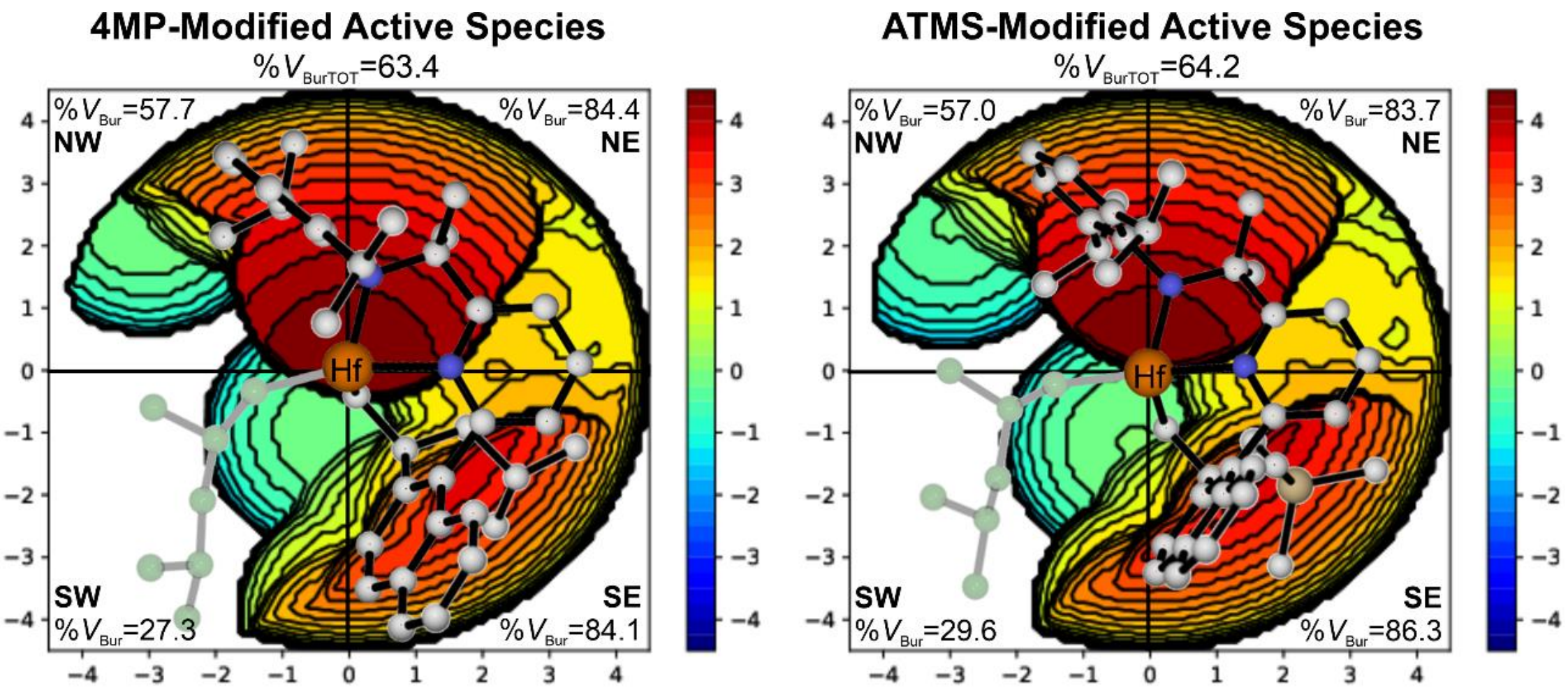

Figure S22. Steric maps of buried volume (%V_{Bur}) computed on the 4MP- and ATMS-modified propagating active species. The buried volume was computed using a sphere of 4.5 Å radius centered on the Hf atom.

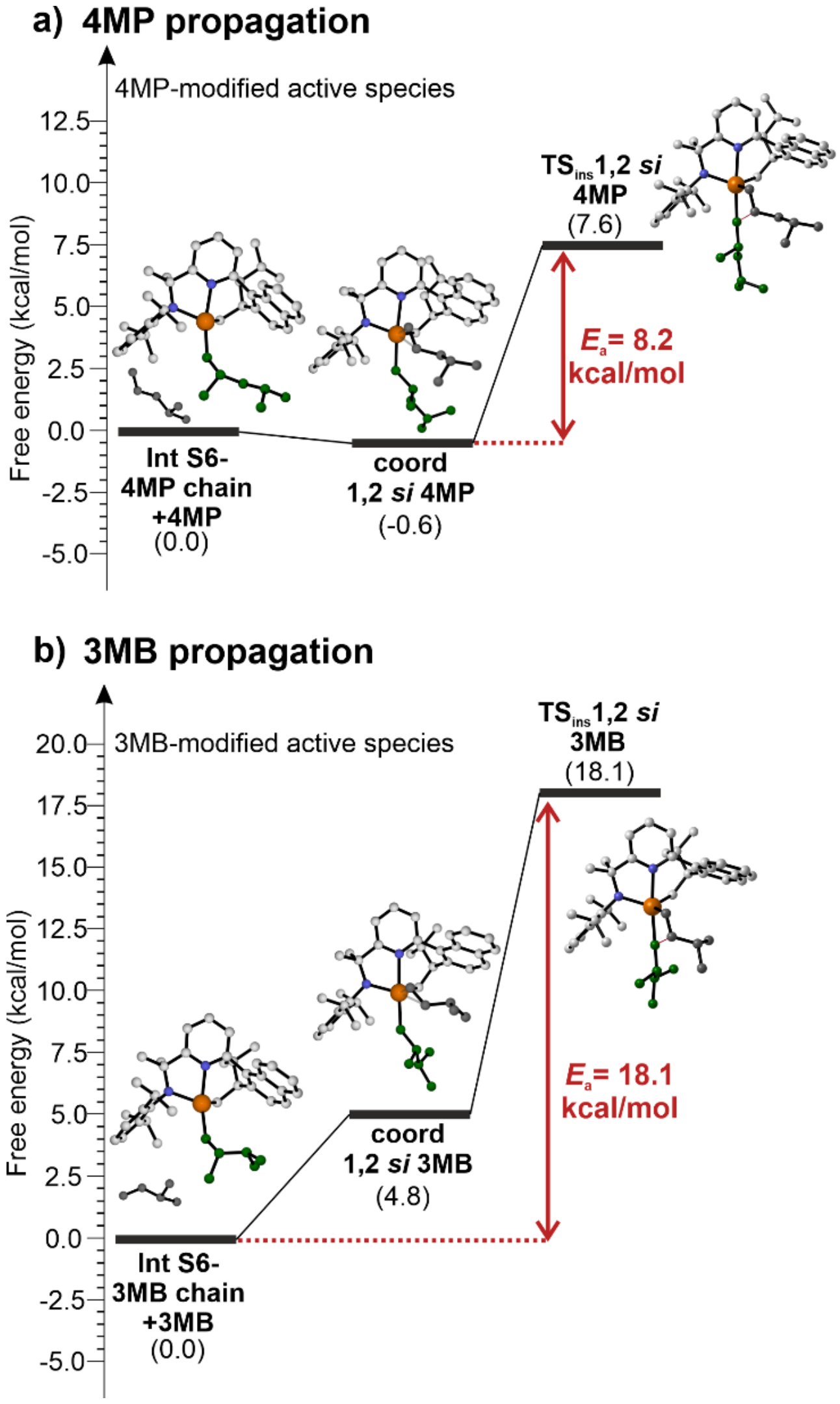

Figure S23. DFT free energy profiles for (a) 3MB and (b) 4MP propagation promoted by the corresponding monomer-inserted (left panels) and ATMS-modified (right panels) active species.

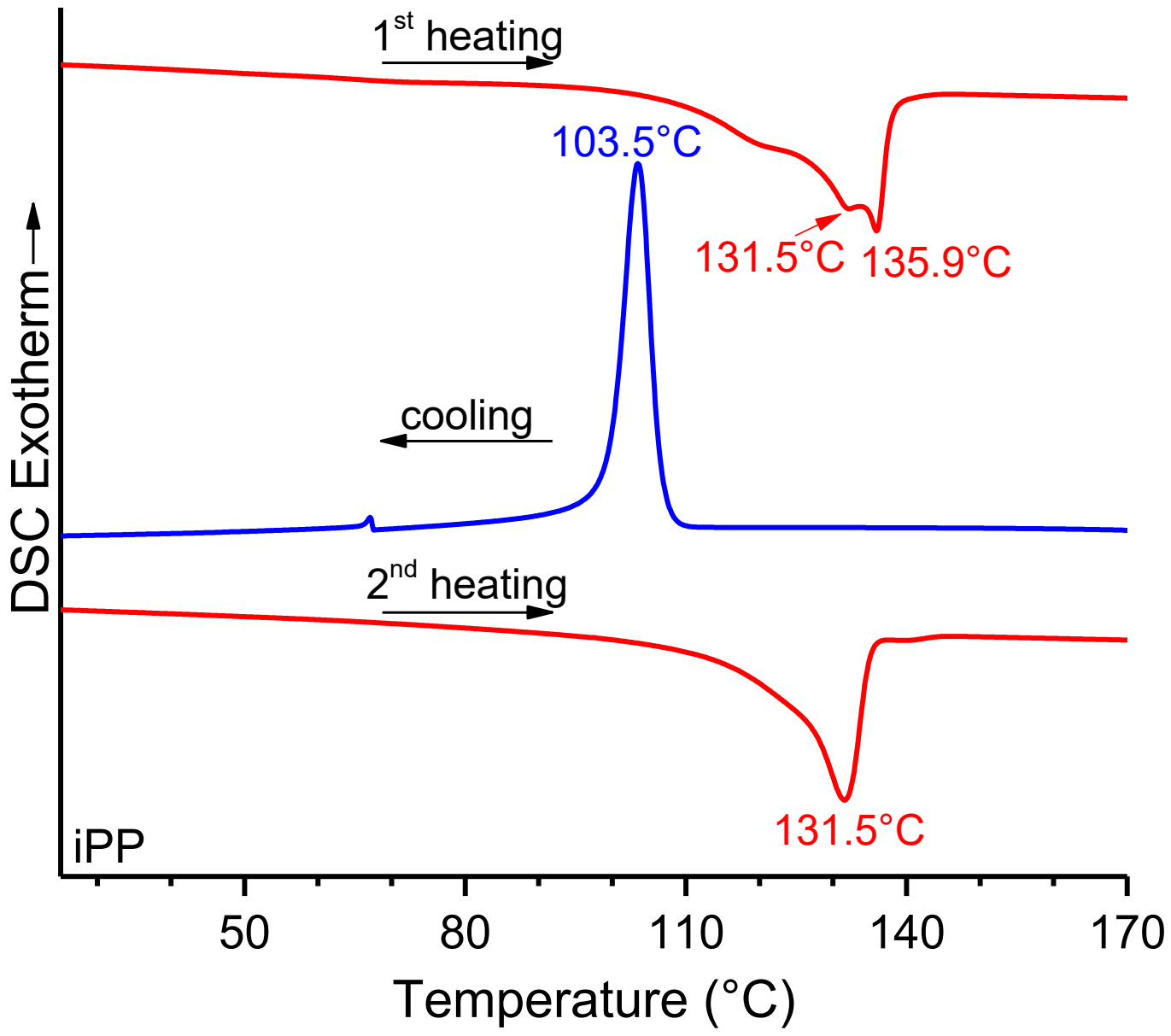

Figure S24. DSC thermograms of the sample iPP (entry 1 of Table S12) synthesized using the pyridylamido-Hf complex **1a** shown in Scheme 1, displaying the first heating, cooling, and successive second heating scans recorded at a scan rate of 10 °C min^{-1}.

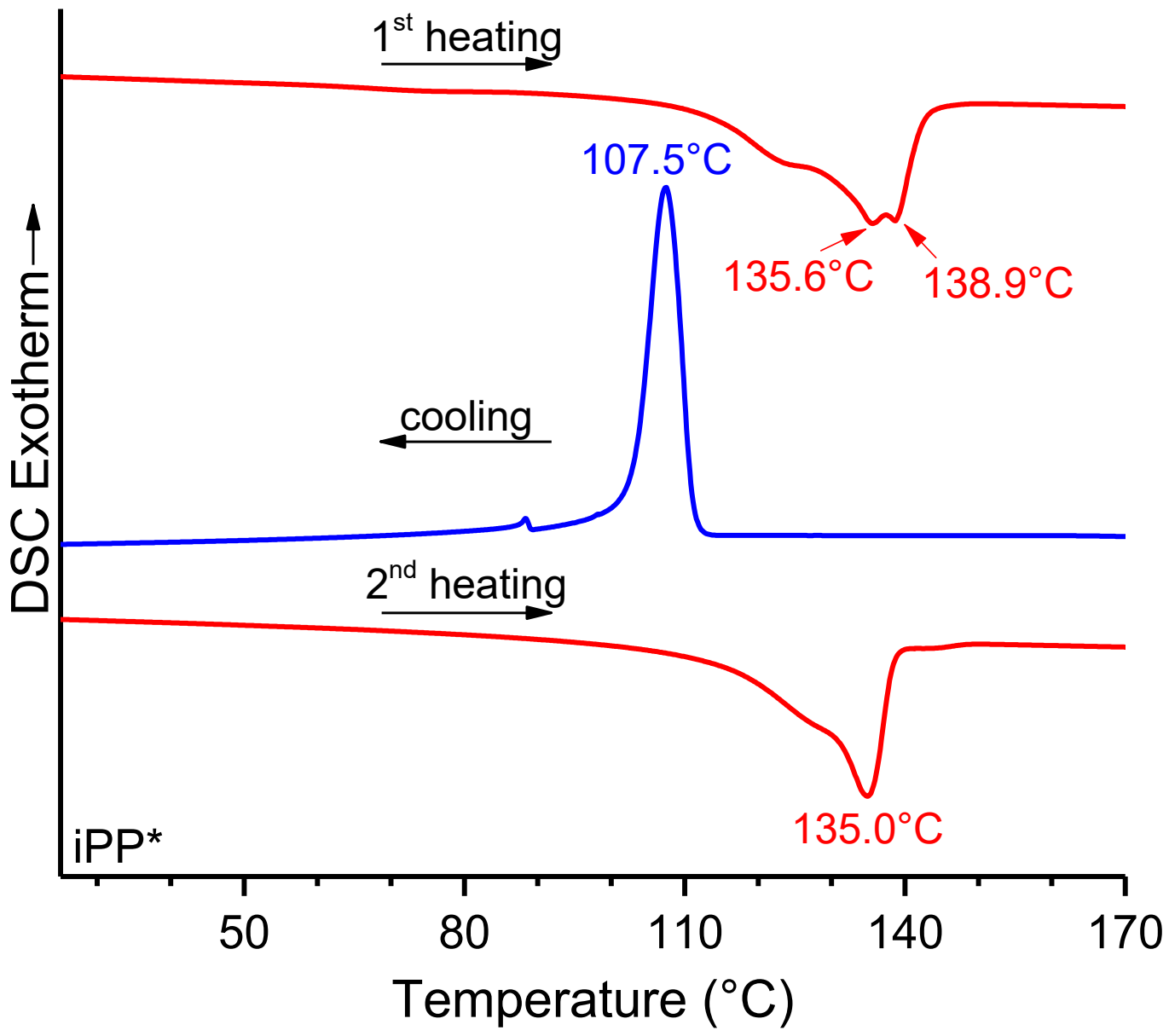

Figure S25. DSC thermograms of the sample iPP* (entry 2 of Table S12) synthesized using the ATMS-pre-activated pyridylamido-Hf complex **1a** shown in Scheme 1 (ATMS/precatalyst = 20:1), displaying the first heating, cooling, and successive second heating scans recorded at a scan rate of 10 °C min^{-1}.

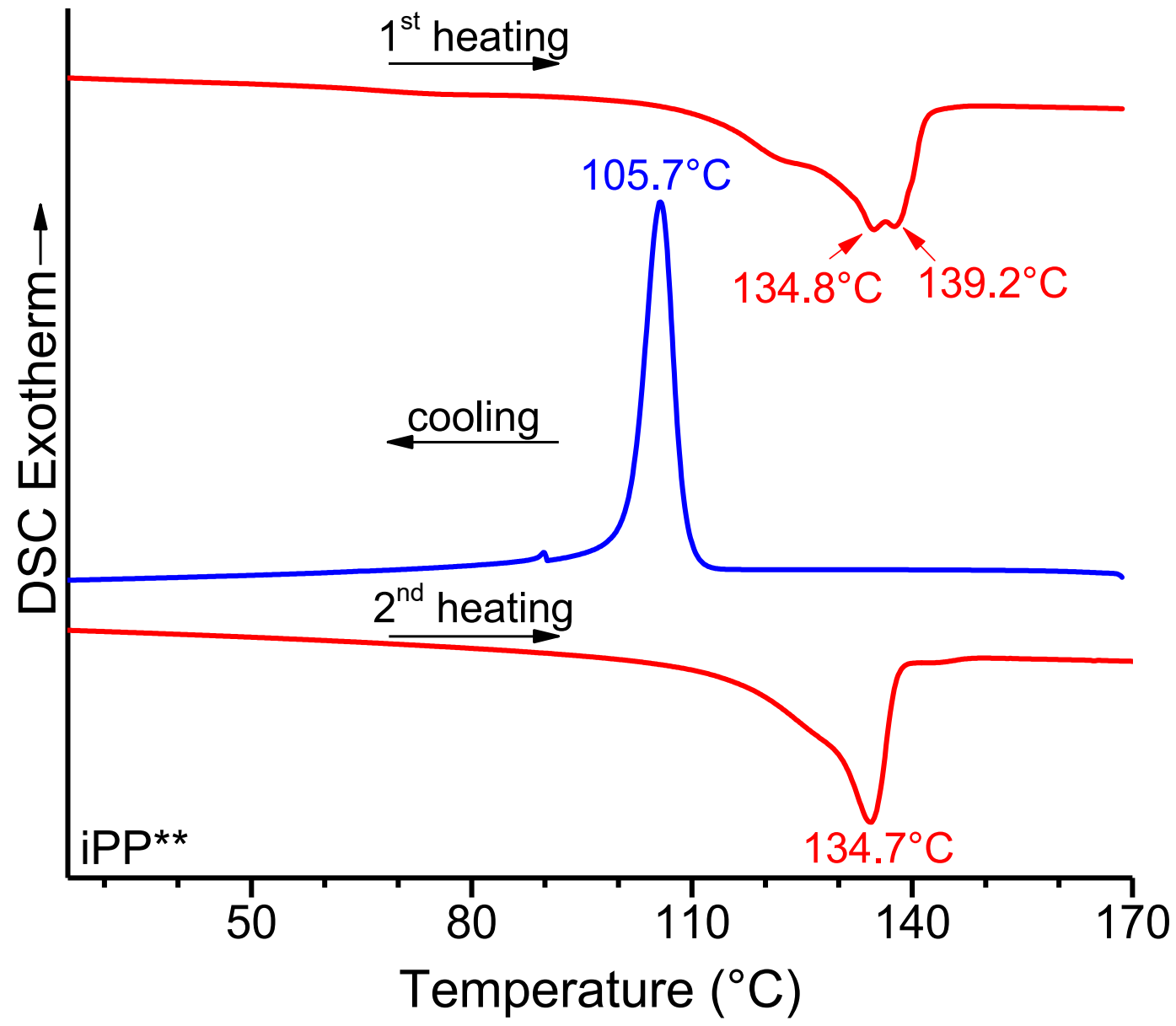

Figure S26. DSC thermograms of the sample iPP** (entry 3 of Table S12) synthesized using the ATMS-pre-activated pyridylamido-Hf complex **1a** in Scheme 1 (ATMS/precatalyst = 5:1), displaying the first heating, cooling, and successive second heating scans recorded at a scan rate of 10 °C min^{-1}.

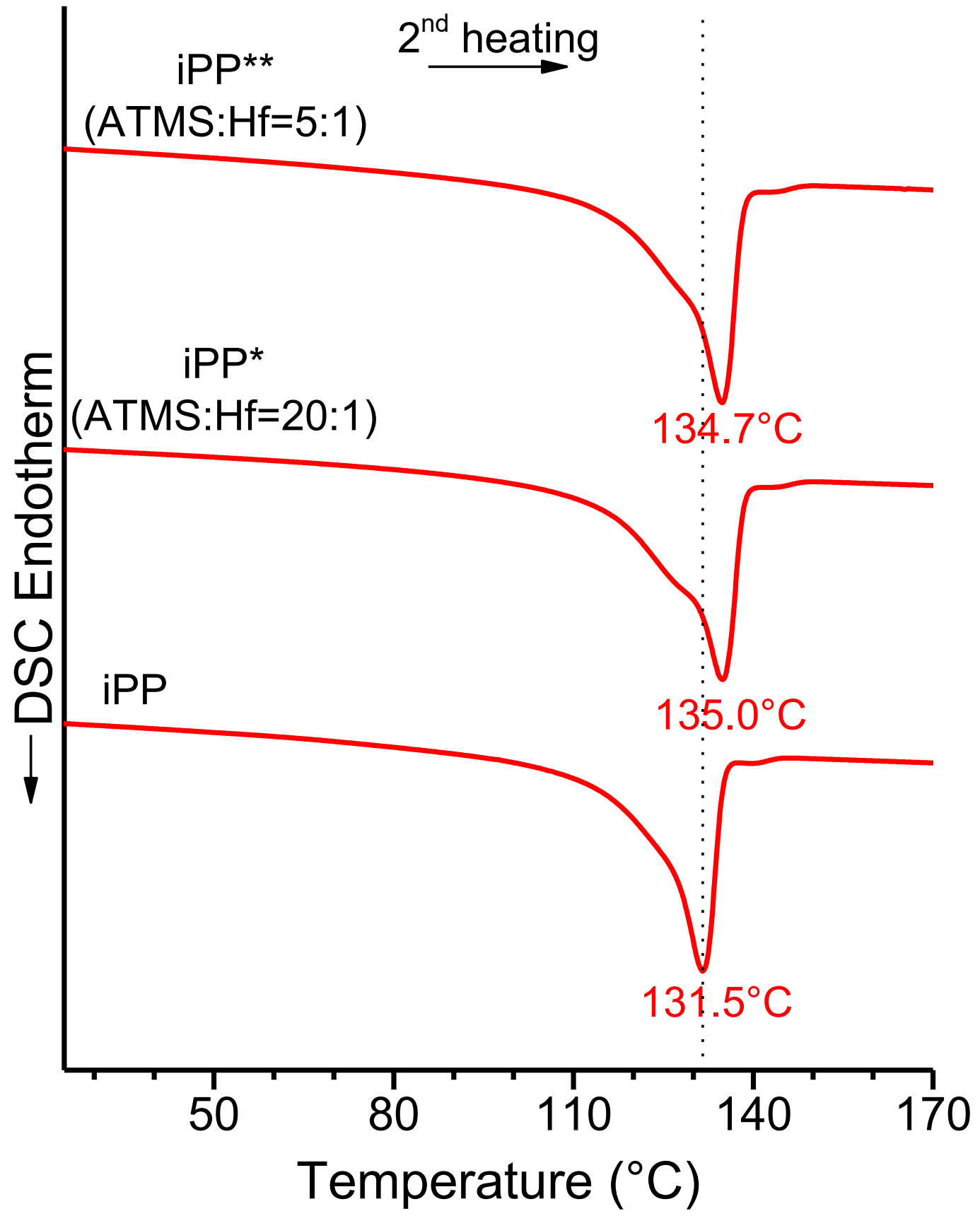

Figure S27. Comparison of the second heating DSC curves of iPP samples synthesized with the pyridylamido-Hf complex **1a** in Scheme 1 without the ATMS pre-activation step (sample iPP) and in the presence of ATMS pre-activation at ATMS/Hf ratios of 20:1 (sample iPP*) and 5:1 (sample iPP**).

Table S14. Melting temperature (T_m^I) and enthalpy (ΔH_m^I) recorded during first heating of as-prepared samples, crystallization temperature (T_c) and enthalpy (ΔH_c) recorded upon cooling from the melt and melting temperature (T_m^{II}) and enthalpy (ΔH_m^{II}) recorded during second heating after melt-crystallization of samples iPP, iPP* and iPP** extracted from DSC curves of Figures S24-S26.

Sample	T_m^I (°C)	T_c (°C)	T_m^{II} (°C)	ΔH_m^I (J·g^{-1})	ΔH_c (J·g^{-1})	ΔH_m^{II} (J·g^{-1})
**iPP**	131.5, 139.5	103.5	131.5	85.9	84.3	86.6
**iPP***	135.6, 138.9	107.5	135.0	92.4	87.5	93.1
**iPP****	134.8, 139.2	105.7	134.7	92.3	88.8	89.4

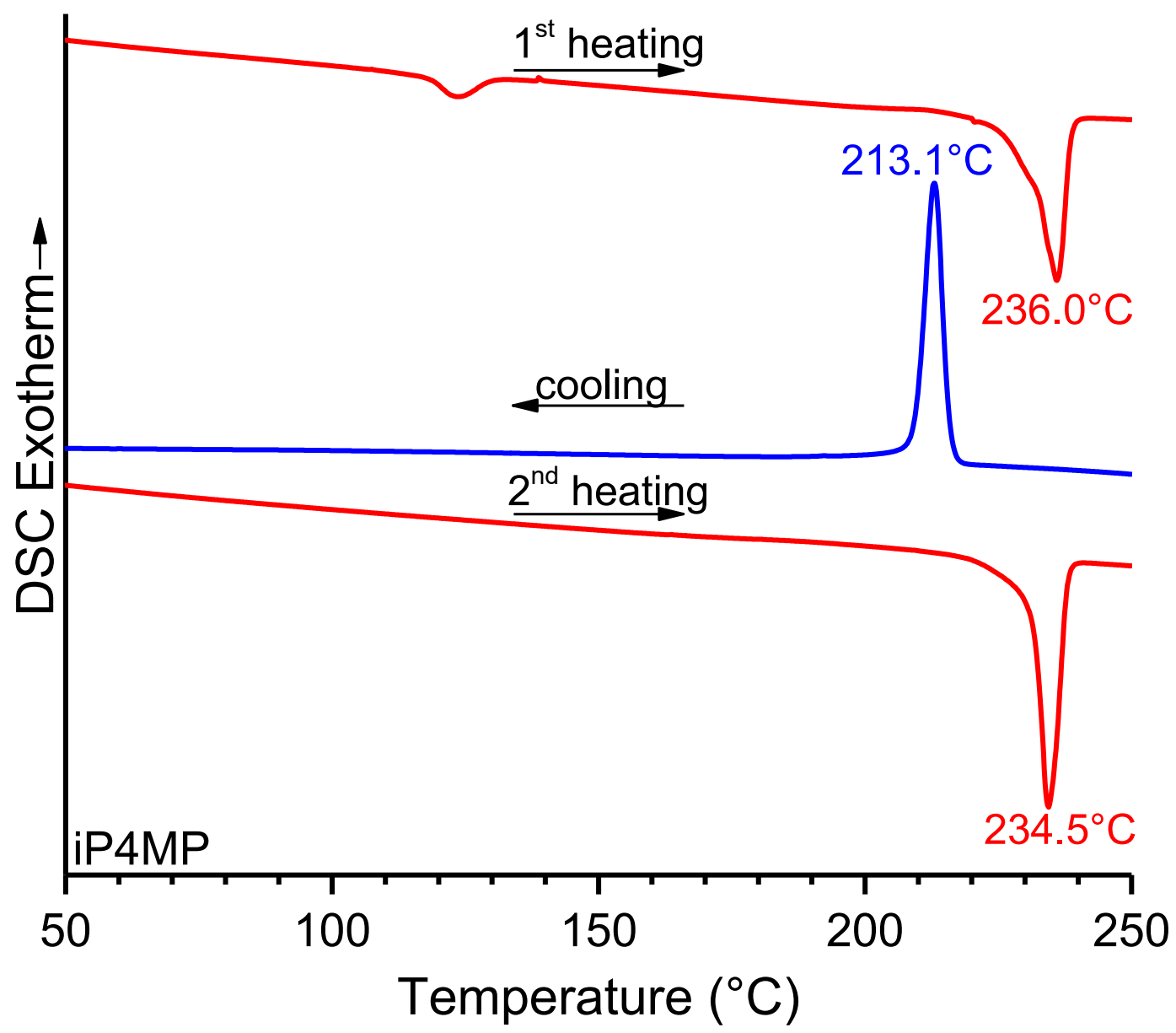

Figure S28. DSC thermograms of the sample iP4MP (entry 1 of Table S13) synthesized using the pyridylamido-Hf complex **1a** shown in Scheme 1, displaying the first heating, cooling, and successive second heating scans recorded at a scan rate of 10 °C min^{-1}.

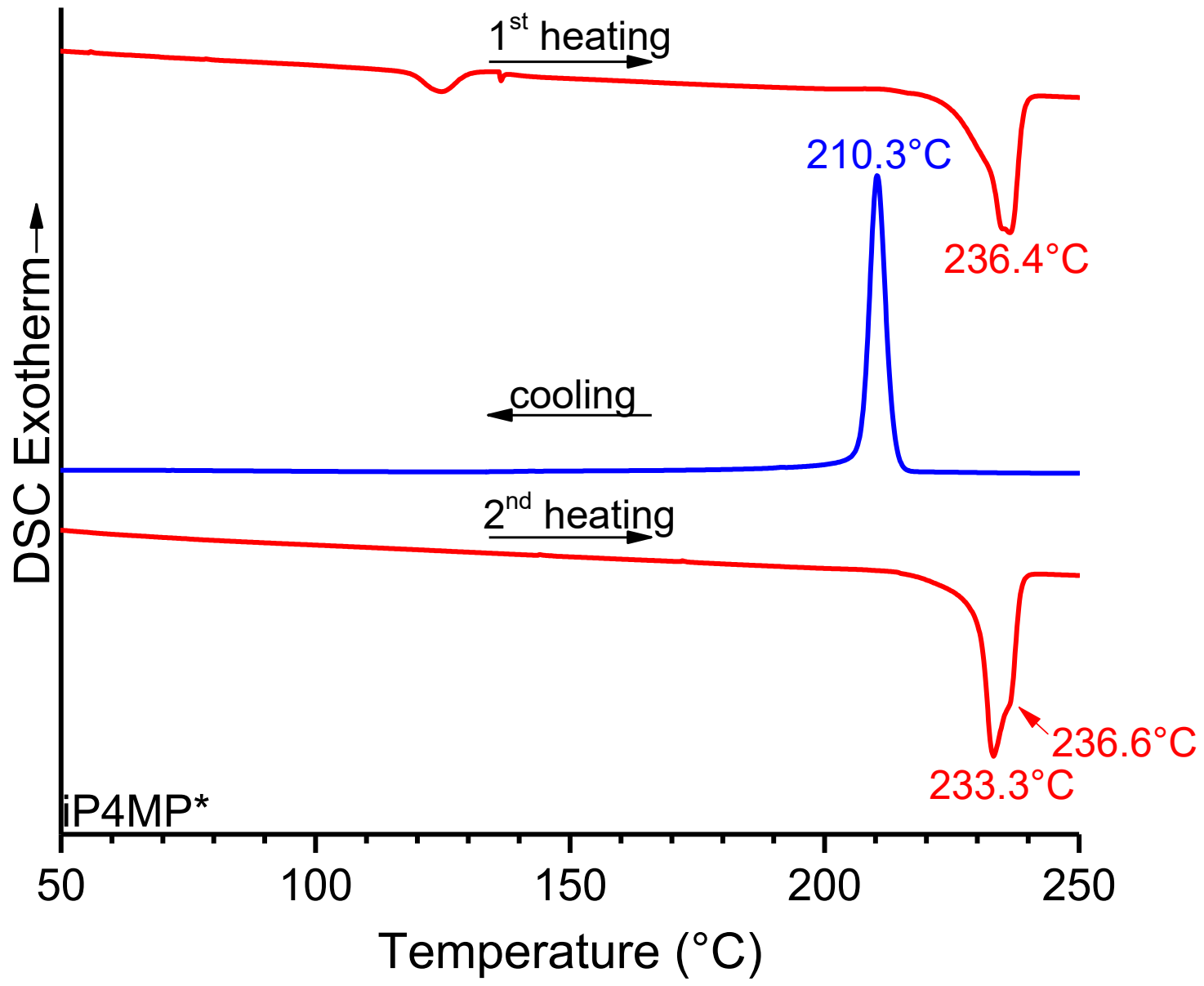

Figure S29. DSC thermograms of the sample iP4MP* (entry 2 of Table S13 synthesized using the ATMS-pre-activated pyridylamido-Hf complex **1a** in Scheme 1 (ATMS/precatalyst = 20:1), displaying the first heating, cooling, and successive second heating scans recorded at a scan rate of 10 °C min^{-1}.

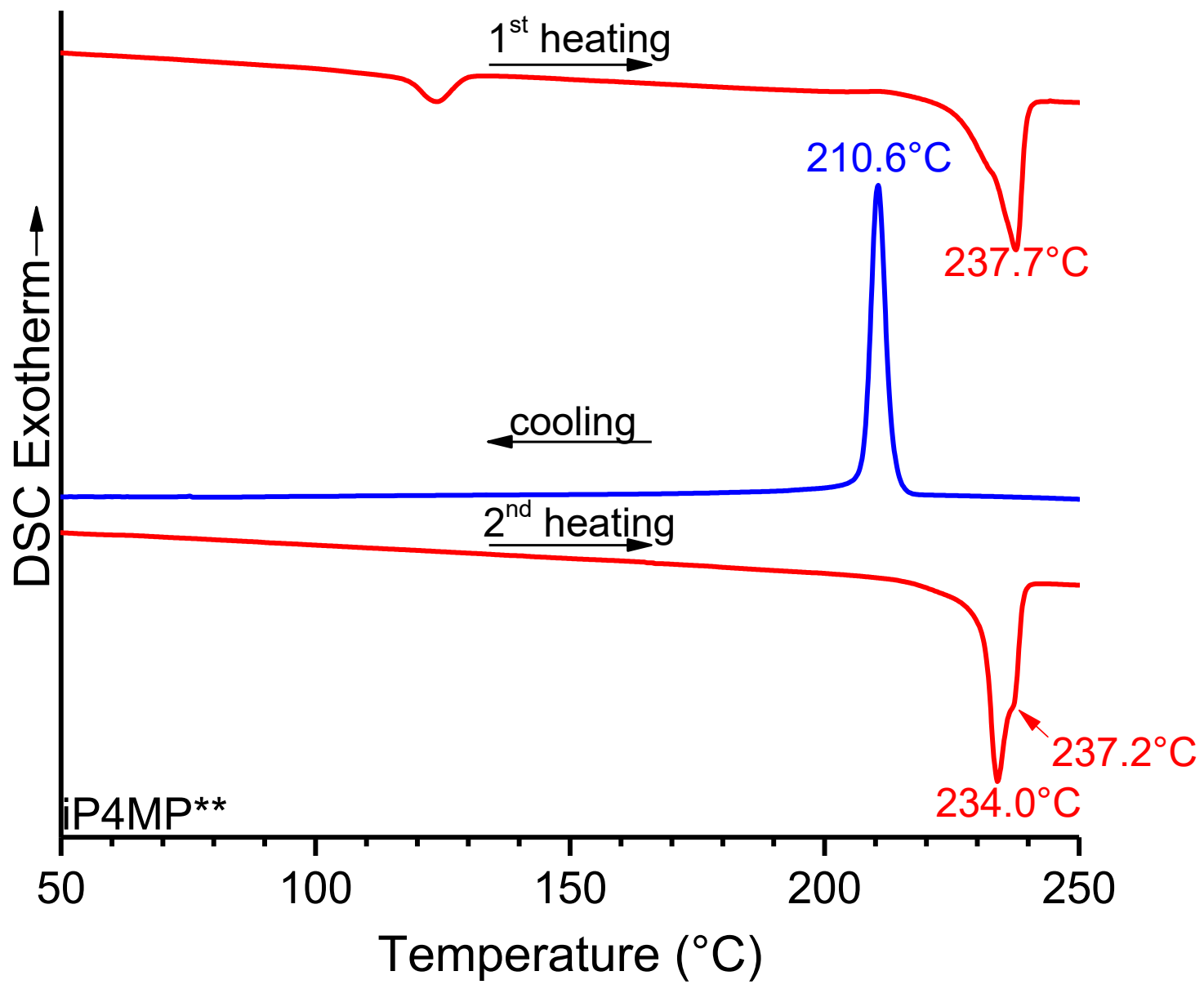

Figure S30. DSC thermograms of the sample iP4MP** (entry 3 of Table S13) synthesized using the ATMS-pre-activated pyridylamido-Hf complex **1a** in Scheme 1 (ATMS/precatalyst = 5:1), displaying the first heating, cooling, and successive second heating scans recorded at a scan rate of 10 °C min^{-1}.

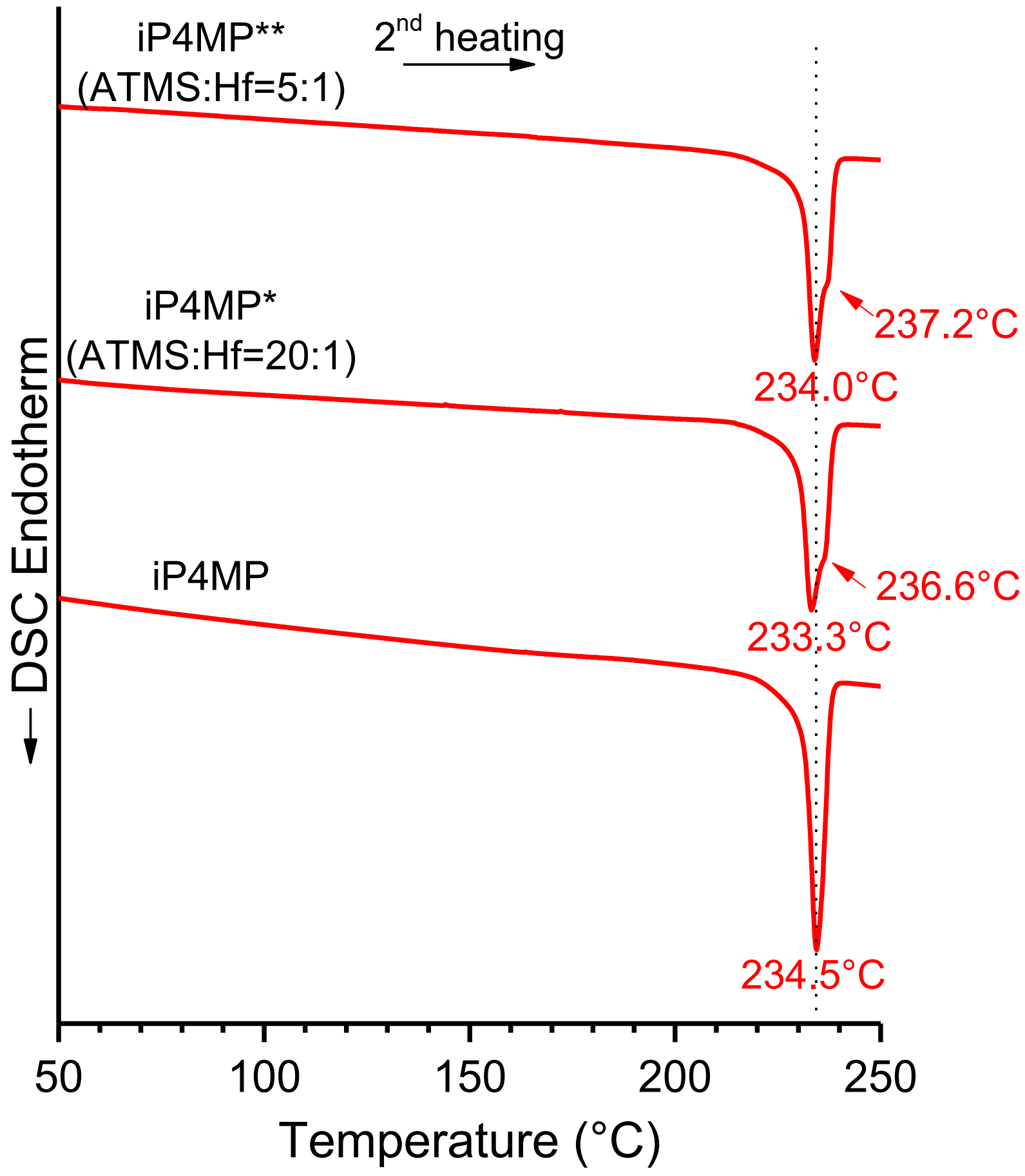

Figure S31. Comparison of the second heating DSC curves of iP4MP samples synthesized with the pyridylamido-Hf complex **1a** in Scheme 1 without the ATMS pre-activation step (sample iP4MP) and in the presence of ATMS pre-activation at ATMS/Hf ratios of 20:1 (sample iP4MP*) and 5:1 (sample iP4MP**).

Table S15. Melting temperature (T_m^I) and enthalpy (ΔH_m^I) recorded during first heating of as-prepared samples, crystallization temperature (T_c) and enthalpy (ΔH_c) recorded upon cooling from the melt and melting temperature (T_m^{II}) and enthalpy (ΔH_m^{II}) recorded during second heating after melt-crystallization of samples iP4MP, iP4MP* and iP4MP* extracted from DSC curves of Figures S28-S30.

Sample	T_m^I (°C)	T_c (°C)	T_m^{II} (°C)	ΔH_m^I (J·g^{-1})	ΔH_c (J·g^{-1})	ΔH_m^{II} (J·g^{-1})
**iP4MP**	236.0	213.1	234.5	48.8	56.3	56.1
**iP4MP***	236.4	210.3	233.2, 236.6	43.8	51.3	52.2
**iP4MP****	237.7	210.6	234.0, 237.2	46.5	51.8	53.1

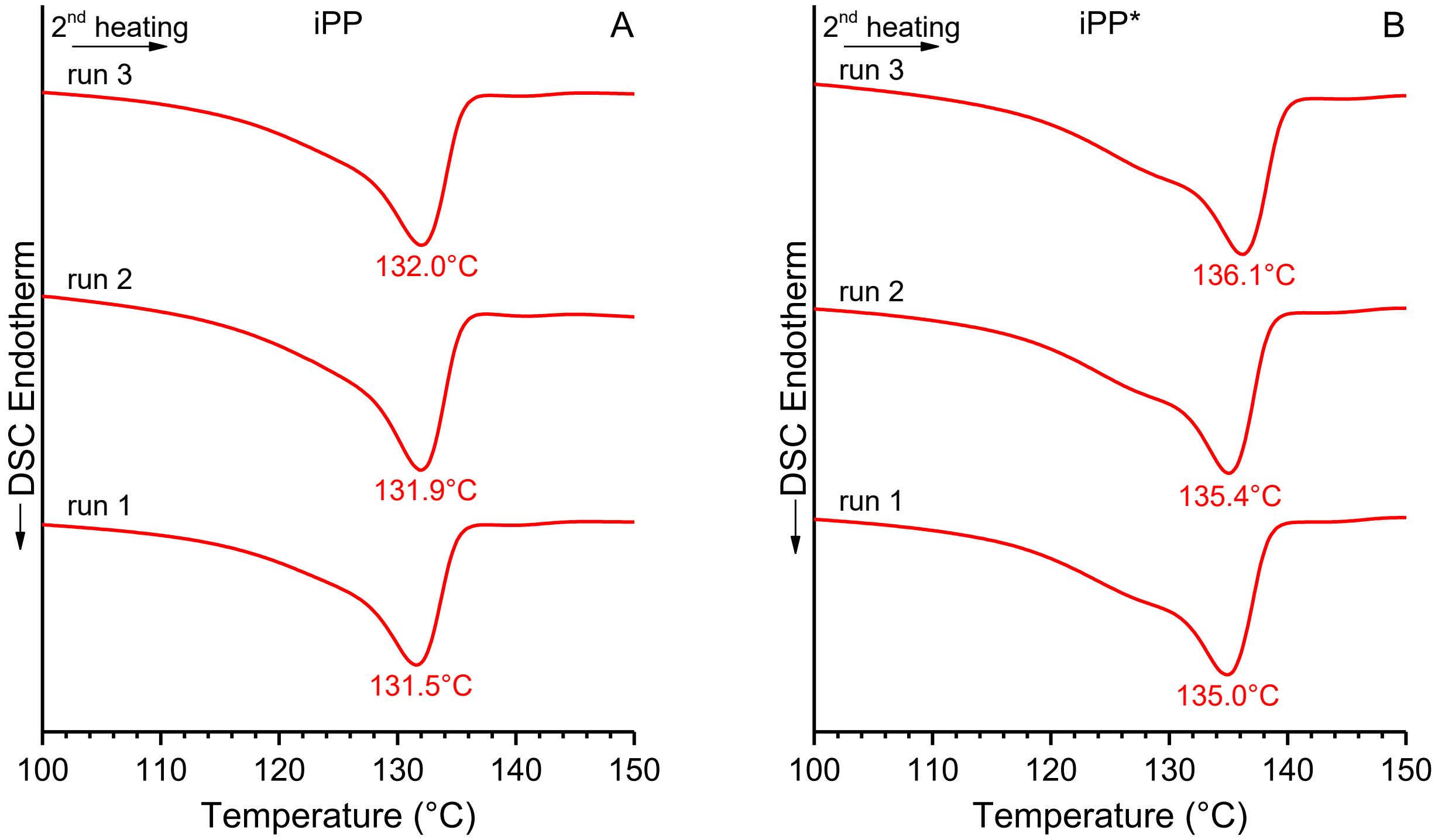

Figure S32. Second-heating DSC curves of three independent iPP samples obtained from separate polymerization runs using (A) the conventional activation procedure and (B) the ATMS pre-activation strategy.

Table S16. Melting temperatures determined for iPP samples obtained with catalyst **1a** from three independent polymerization experiments under conventional conditions (Figure S32A) and following ATMS pre-activation (Figure S32B).

**Sample**	**Catalyst activation protocol**	$T_{m,1}$ **(°C)**[a]	$T_{m,2}$ **(°C)**[a]	$T_{m,3}$ **(°C)**[a]	T_m **± SD (°C)**
**iPP**	Conventional activation	131.5	131.9	132.0	131.8 ± 0.3
**iPP***	ATMS pre-activation	135.0	135.4	136.1	135.5 ± 0.6

[a] T_m values were determined from the second-heating DSC curves reported in Figures S32A and S32B.

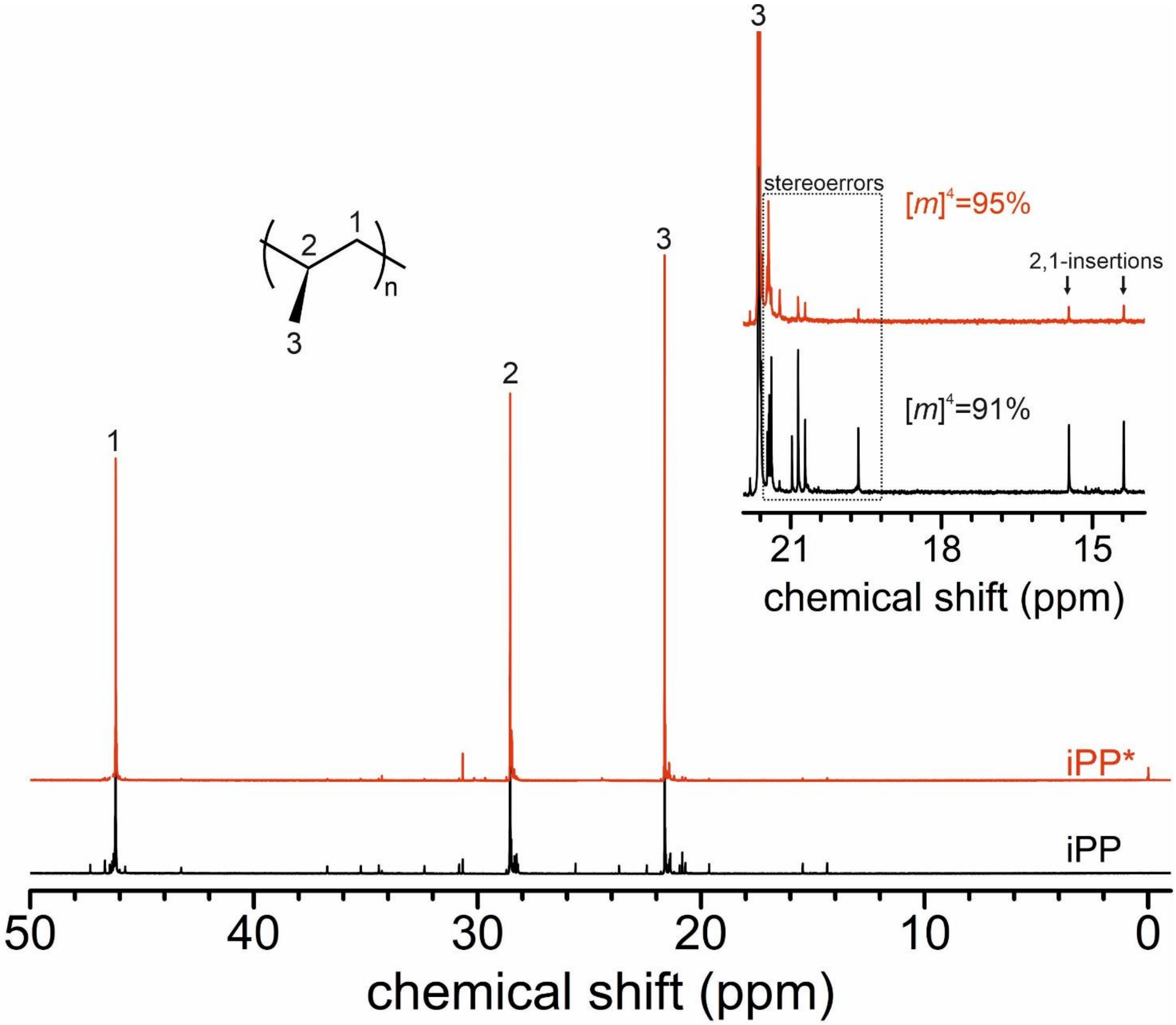

Figure S33. Comparison between ^{13}C NMR spectra of iPP (black line) and iPP* (red line) samples synthesized with the pyridylamido-Hf complex **1a** in Scheme 1 without (sample iPP) and with the ATMS pre-activation step (sample iPP*). An enlargement of the methyl stereoerrors region is shown in the inset. In agreement with previous literature,[S21] the iPP sample synthesized without the ATMS pre-activation step exhibits an $[m]^4$ value of 91%, whereas the sample iPP*, synthesized with the ATMS-pre-activation step shows an $[m]^4$ of 95%. However, this value may be slightly overestimated due to the presence of low-intensity signals in the methyl region, attributable to short ATMS-ATMS sequences formed during the prepolymerization step and therefore located at the beginning of the polymer chain. Nevertheless, the comparison clearly shows that the intensities of signals associated with both stereo- and regio-defects are significantly lower in the sample iPP* than in the sample iPP, demonstrating the higher isoselectivity of the ATMS-modified catalysts.

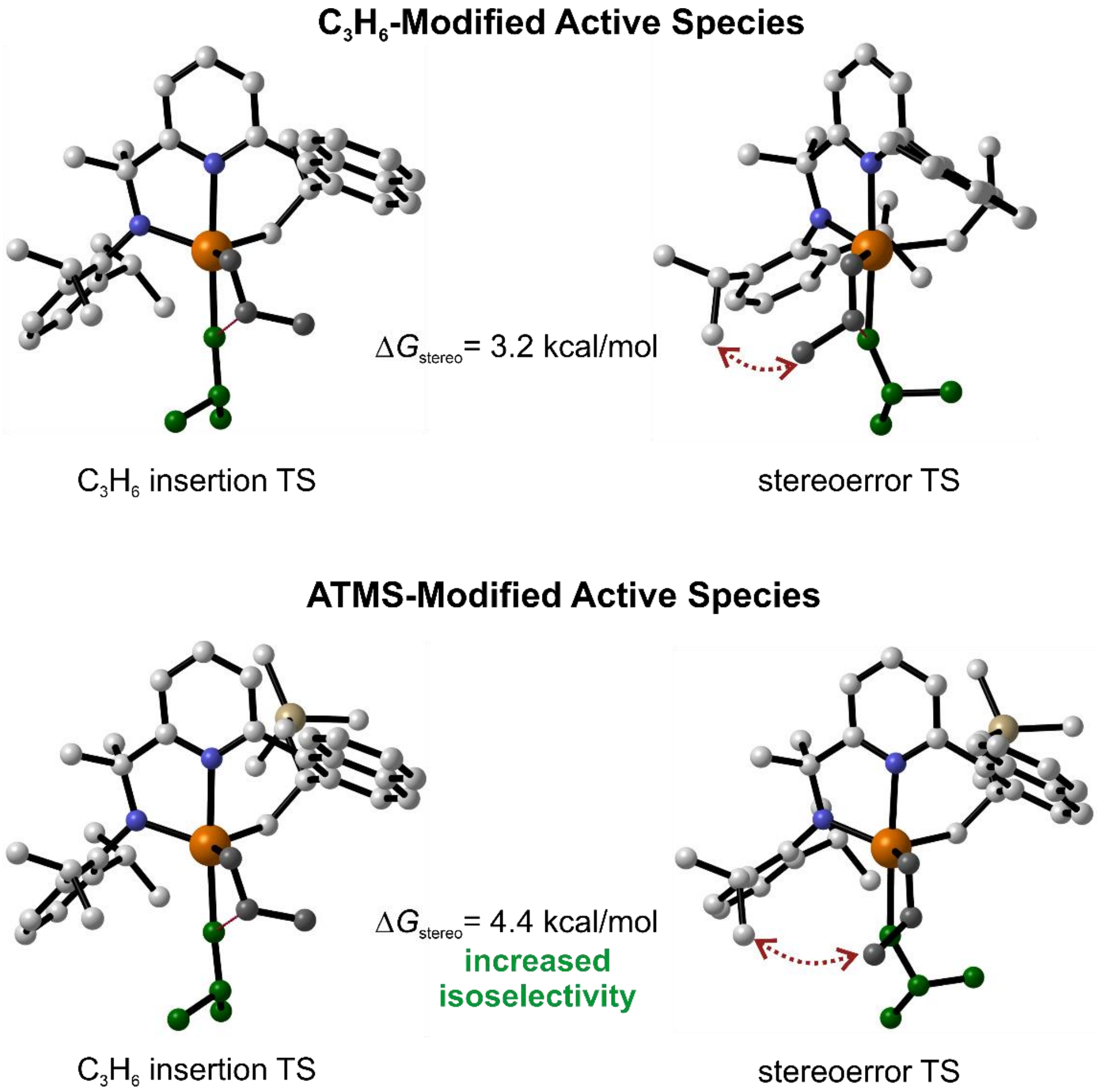

Figure S34. DFT optimized geometries for propene insertion and stereoerror TSs in the presence of propene-modified (top) and ATMS-modified active species (bottom). Free energies of stereoselectivity (ΔG_{stereo}) are reported. The trend calculated by DFT results agrees with the ^{13}C NMR data reported in Figure S33.

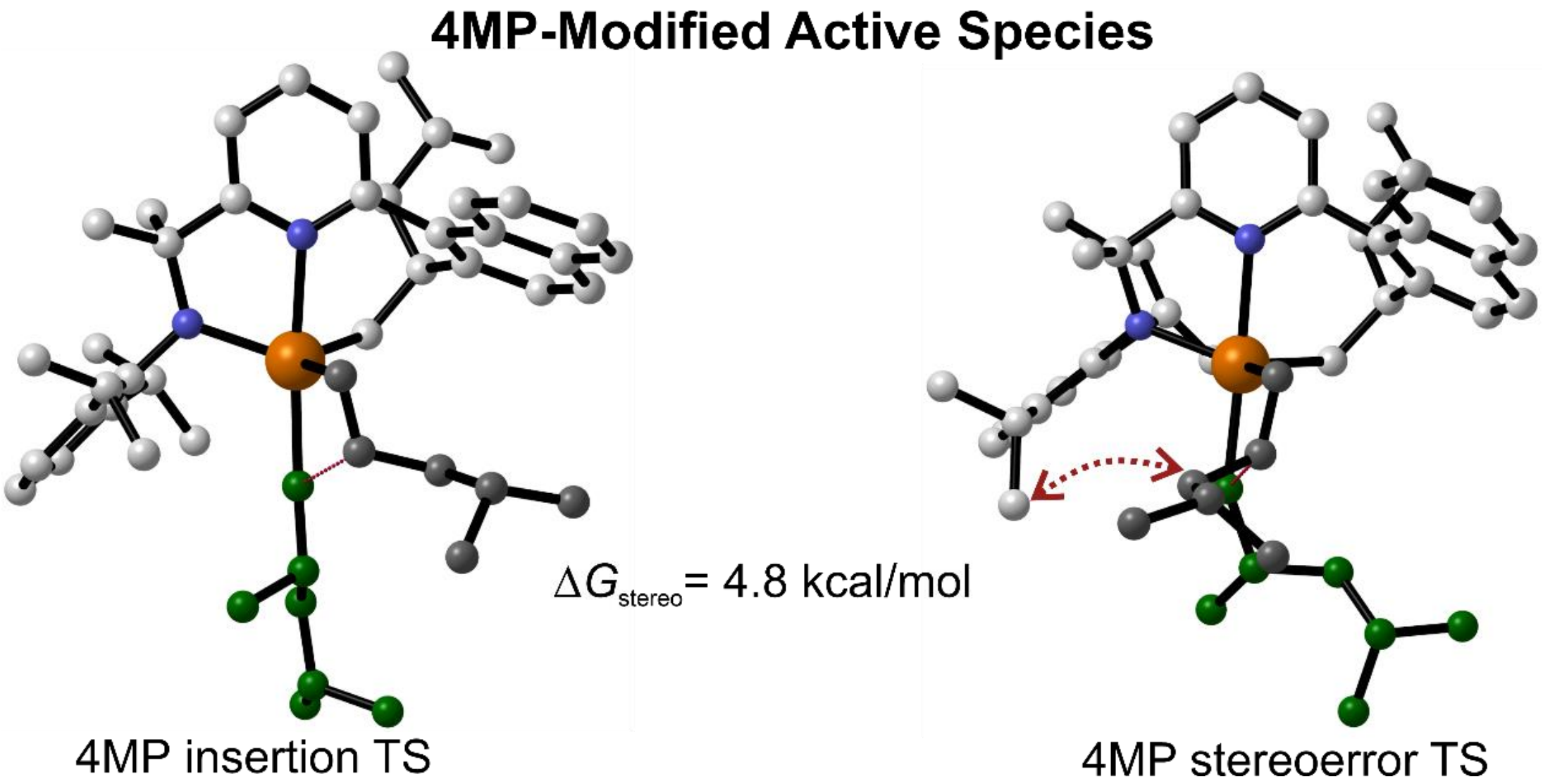

Figure S35. DFT optimized geometries for 4MP insertion and stereoerror TSs in the presence of 4MP-modified active species. Free energies of stereoselectivity ($\Delta G_{\mathrm{stereo}}$) are reported.

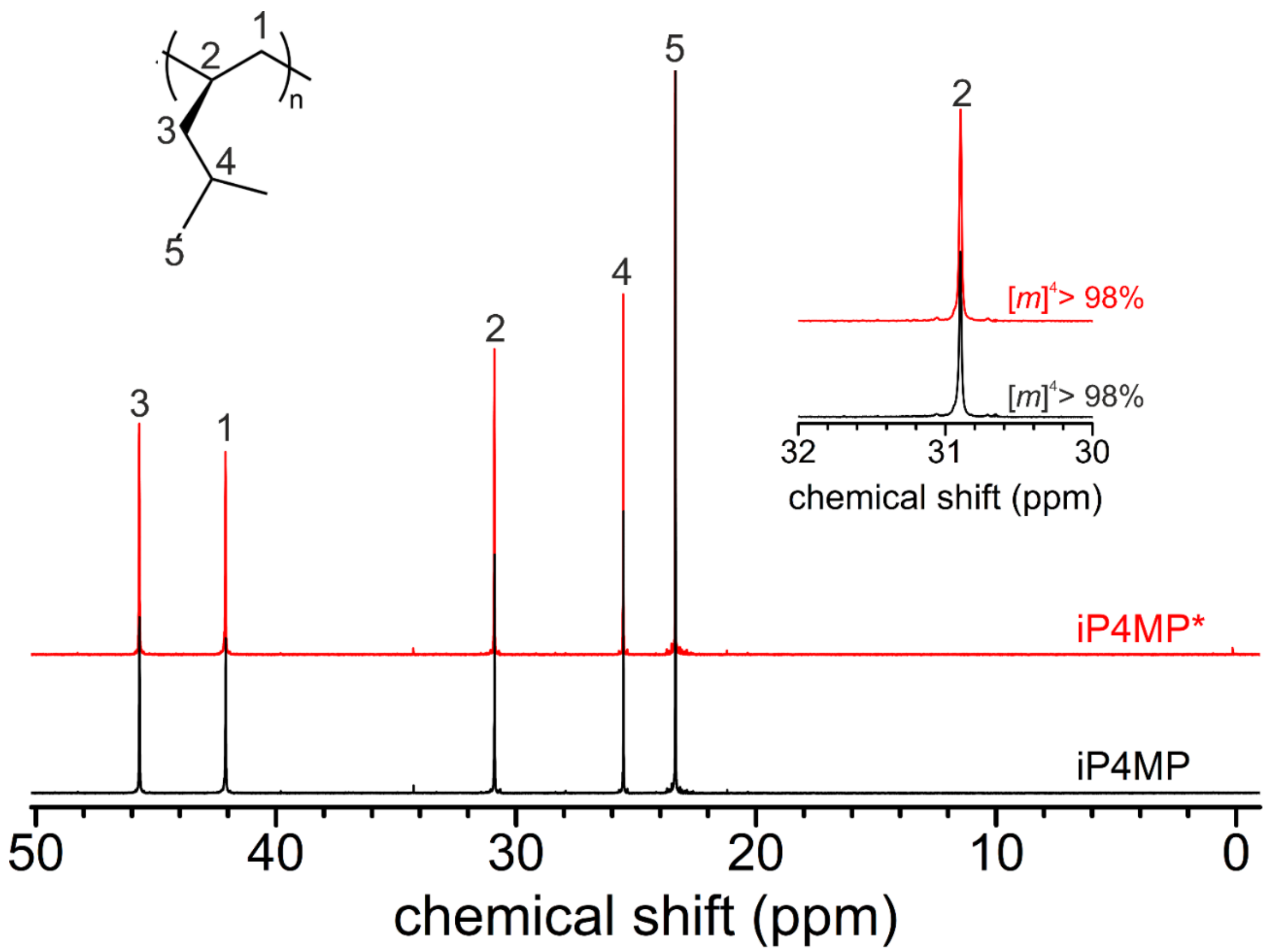

Figure S36. Comparison between ^{13}C NMR spectra of samples iP4MP (black line) and iP4MP* (red line). In both cases the calculated $[m]^4$ is equal to 98%.

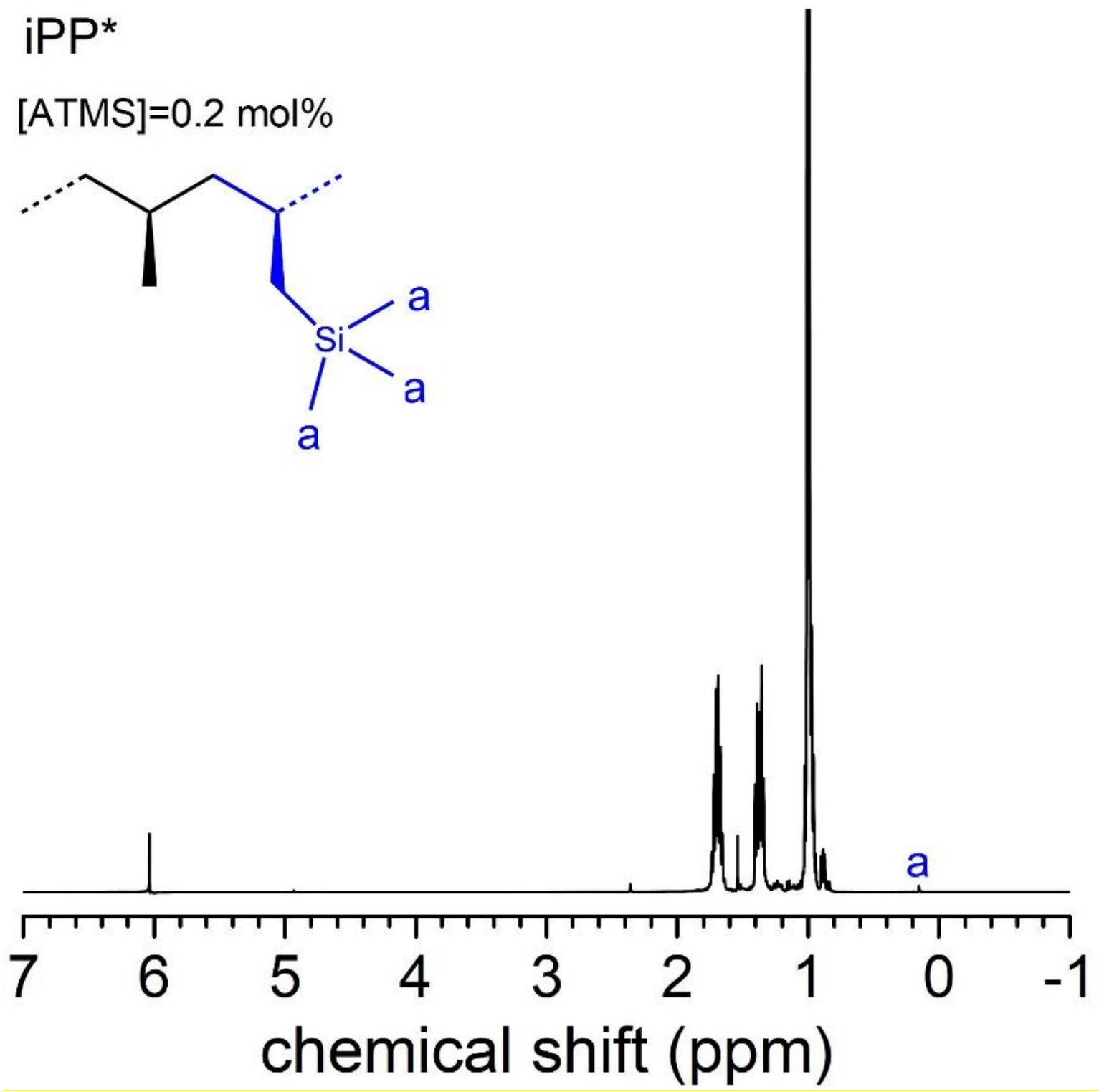

Figure S37. ^{1}H NMR spectrum of the iPP* sample obtained using catalyst **1a** and the ATMS pre-activation procedure (20 equiv. ATMS, 5 min pre-activation time). The resonance assigned to the protons of the methyl groups bonded to silicon (signal a) was used to determine an ATMS content of 0.2 mol%, according to the procedure described in the Experimental Section.[S1]

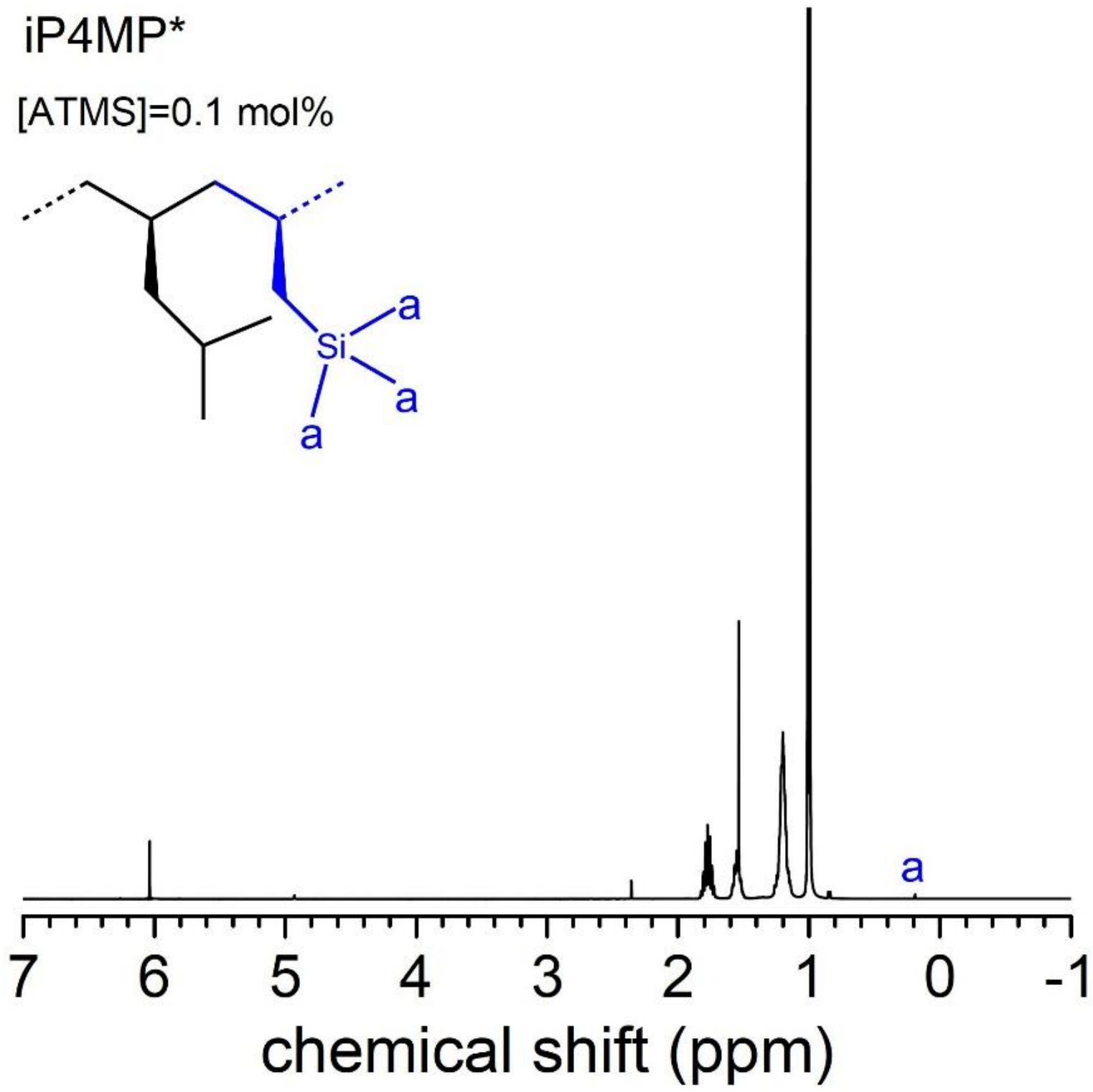

Figure S38. ^{1}H NMR spectrum of the iP4MP* sample obtained using catalyst **1a** and the ATMS pre-activation procedure (20 equiv. ATMS, 5 min pre-activation time). The resonance assigned to the protons of the methyl groups bonded to silicon (signal a) was used to determine an ATMS content of 0.1 mol%, according to the procedure described in the Experimental Section.[S1]

3) References

[S1] F. De Stefano, M. Scoti, A. Giordano, C. De Rosa, R. Di Girolamo, "Silane-functionalized isotactic polypropylene: A direct route to advanced polyolefin materials" *Materials Today Chemistry* **52**, (2026): 103361.

[S2] M. J. Frisch, G. W. Trucks, H. B. Schlegel, G. E. Scuseria, M. A. Robb, J. R. Cheeseman, G. Scalmani, V. Barone, G. A. Petersson, H. Nakatsuji, X. Li, M. Caricato, A. V. Marenich, J. Bloino, B. G. Janesko, R. Gomperts, B. Mennucci, H. P. Hratchian, J. V. Ortiz, A. F. Izmaylov, J. L. Sonnenberg, F. Williams, F. Ding, F. Lipparini, F. Egidi, J. Goings, B. Peng, A. Petrone, T. Henderson, D. Ranasinghe, V. G. Zakrzewski, J. Gao, N. Rega, G. Zheng, W. Liang, M. Hada, M. Ehara, K. Toyota, R. Fukuda, J. Hasegawa, M. Ishida, T. Nakajima, Y. Honda, O. Kitao, H. Nakai, T. Vreven, K. Throssell, J. A. Montgomery Jr., J. E. Peralta, F. Ogliaro, M. J. Bearpark, J. J. Heyd, E. N. Brothers, K. N. Kudin, V. N. Staroverov, T. A. Keith, R. Kobayashi, J. Normand, K. Raghavachari, A. P. Rendell, J. C. Burant, S. S. Iyengar, J. Tomasi, M. Cossi, J. M. Millam, M. Klene, C. Adamo, R. Cammi, J. W. Ochterski, R. L. Martin, K. Morokuma, O. Farkas, J. B. Foresman, D. J. Fox, *Gaussian 16 Revision C.01*, Gaussian Inc., Wallingford, CT, (2016).

[S3] A. D. Becke, "Density-functional exchange-energy approximation with correct asymptotic behavior", *Physical Review A* **38**, (1988): 3098-3100.

[S4] C. Lee, W. Yang, R. G. Parr, "Development of the Colle-Salvetti correlation-energy formula into a functional of the electron density", *Physical Review B* **37**, (1988): 785-789.

[S5] A. Schäfer, H. Horn, R. Ahlrichs, "Fully optimized contracted Gaussian basis sets for atoms Li to Kr", *Journal of Chemical Physics* **97**, (1992): 2571-2577.

[S6] P. J. Hay, W. R. Wadt, "Ab initio effective core potentials for molecular calculations. Potentials for potassium to gold including the outermost core orbitals", *Journal of Chemical Physics* **82**, (1985): 299-310.

[S7] F. Weigend, F. Furche, R. Ahlrichs, "Gaussian basis sets of quadruple zeta valence quality for atoms H–Kr", *Journal of Chemical Physics* **119**, (2003): 12753–12762.

[S8] W. R. Wadt, P. J. Hay, "Ab initio effective core potentials for molecular calculations. Potentials for main group elements sodium to bismuth", *Journal of Chemical Physics* **82**, (1985): 284–298.

[S9] S. Grimme, J. Antony, S. Ehrlich, H. Krieg, "A consistent and accurate ab initio parametrization of density functional dispersion correction (DFT-D) for the 94 elements H–Pu", *Journal of Chemical Physics* **132**, (2010): 154104.

[S10] V. Barone, M. Cossi, "Quantum calculation of molecular energies and energy gradients in solution by a conductor solvent model", *Journal of Physical Chemistry A* **102**, (1998): 1995-2001.

[S11] R. L. Martin, P. J. Hay, L. R. Pratt, "Hydrolysis of ferric ion in water and conformational equilibrium", *Journal of Physical Chemistry A* **102**, (1998): 3565-3573.

[S12] L. Falivene, V. Barone, G. Talarico, "Unraveling the role of entropy in tuning unimolecular vs. bimolecular reaction rates: The case of olefin polymerization catalyzed by transition metals", *Molecular Catalysis* **452**, (2018): 138-144.

[S13] O. D'Anania, E. Romano, V. Barone, G. Talarico, "Predicting the propene stereoselectivity on transition metal catalysts: A daunting task for density functional theory", *Journal of Computational Chemistry* **45**, (2024): 1483-1492.

[S14] O. D'Anania, F. De Stefano, C. De Rosa, G. Talarico, R. Di Girolamo, "Closing the Loop of Cyclopolymerization of Nonconjugated α,ω-Diolefin Diasteroselectivity and α-Olefin Polymerization Enantioselectivity", *ACS Catalysis* **14**, (2024): 16649-16662.

[S15] L. Falivene, L. Cavallo, G; Talarico, "Buried Volume Analysis for Propene Polymerization Catalysis Promoted by Group 4 Metals: A Tool for Molecular Mass Prediction" *ACS Catalysis* **5**, (2015)*:* 6815–6822.

[S16] L. Falivene, Z. Cao, A. Petta, L. Serra, A. Poater, R. Oliva, V. Scarano, L. Cavallo, "Towards the online computer-aided design of catalytic pockets" *Nature Chemistry* **11**, (2019): 872–879.

[S17] G. Natta, G. Mazzanti, P. Longi, F. Bernardini, in *Stereoregular Polymers and Stereospecific Polymerizations*, Elsevier, **1967**, pp. 424-426.

[S18] R. Zeigler, L. Resconi, G. Balbontin, G. Guerra, V. Venditto, C. De Rosa, "Allyltrimethylsilane polymers from metallocene catalysts: tacticity and structural characterization" *Polymer* **35**, (1994): 4648-4655.

[S19] W. Wang, M. Ren, L. Hou, S. Qu, X. Li, Z. Guo, "Polymerization of Allyltrimethylisilane and 4-Methyl-1-Pentene by Using Metallocene Catalysts" *Polymers* **15**, (2023): 2038.

[S20] K. Endo, T. Otsu, "Polymerization of vinylcyclohexane with ziegler–natta catalyst" *Journal of Polymer Science Part A: Polymer Chemistry* **30**, (1992): 679-683.

[S21] S. Segal, A. Yeori, M. Shuster, Y. Rosenberg, M. Kol, "Isospecific Polymerization of Vinylcyclohexane by Zirconium Complexes of Salan Ligands" *Macromolecules* **41**, (2008): 1612-1617.

[S22] G. Theurkauff, K. D. Dauw, O. Miserque, A. Vantomme, J. M. Brusson, J. F. Carpentier, E. Kirillov, "Polymerization of sterically hindered a-olefins with singlesite group 4 metal catalyst precursors" *Polyolefins Journal* **4**, (2017): 123-136.

[S23] A. Borriello, V. Busico, C. De Rosa, D. Schulze, "^{13}C CP/MAS NMR Analysis of Isotactic Poly (3-methyl-1-butene)" *Macromolecules* **28**, (1995): 5679-5680.

[S24] J. M. Eagan, J. Xu, R. Di Girolamo, C. M. Thurber, C. W. Macosko, A. M. LaPointe, F. S. Bates, G. W. Coates, "Combining polyethylene and polypropylene: Enhanced performance with PE/iPP multiblock polymers" *Science* **355**, (2017): 814-816.
